# Environmental DNA detects biodiversity and ecological features of phytoplankton communities in Mediterranean transitional waters

**DOI:** 10.1038/s41598-023-42389-3

**Published:** 2023-09-14

**Authors:** Valeria Specchia, Francesco Zangaro, Eftychia Tzafesta, Benedetta Saccomanno, Maria Rosaria Vadrucci, Maurizio Pinna

**Affiliations:** 1 Department of Biological and Environmental Sciences and Technologies, DiSTeBA, University of Salento, via Monteroni 165, 73100 Lecce, Italy; 2 NBFC, National Biodiversity Future Center, 90133 Palermo, Italy; 3 Regional Agency for the Environmental Prevention and Protection (ARPA Puglia), Corso Trieste 27, Bari, Italy; 4 Research Centre for Fisheries and Aquaculture of Acquatina di Frigole, DiSTeBA, University of Salento, 73100 Lecce, Italy

**Keywords:** Genetics, Ecology, Biodiversity, Community ecology, Ecological genetics, Molecular ecology

## Abstract

Climate changes and anthropogenic pressures are causing a biodiversity decline in terms of species number and genetic diversity, reducing the adaptability and evolvability of natural communities. Transitional water ecosystems are more sensitive to habitat reduction and degradation and, thus, are more exposed to biodiversity declines requiring biodiversity monitoring programs for their conservation. Environmental DNA (eDNA) metabarcoding represents a high-throughput tool for biodiversity assessment that is facilitating data collection for biodiversity monitoring. In this study, we applied, for the first time, eDNA metabarcoding in a Mediterranean coastal lagoon to assess the ecological features of eukaryotic phytoplankton communities. We sampled water in seven different lagoon sites and amplified the extracted DNA with primers targeting the variable region 4 (V4) of the 18S rRNA gene marker. The results demonstrated the validity of eDNA studies to provide insights into lagoon phytoplankton composition, establish the structure and spatial variation of phytoplankton communities, and evaluate its correlation to abiotic factors. Finally, the genetic distances analysis suggests that the different spatial distribution of OTUs, at least for the *Tetraselmis* genus, reflects the genetic background.

## Introduction

Climate changes and anthropogenic pressures are the main drivers of a biodiversity decline characterized by an unprecedented rate of species extinction^1,2^. In addition to a decrease in species composition, habitat reduction and degradation are also causing a loss of species’ genetic diversity^3,4^. The genetic diversity of natural populations and communities ensures adaptive potential and evolvability^5^, reducing the risk of extinction^6^. To address these problems, monitoring actions and conservation strategies are essential. The inter-governmental group on Earth Observations and Biodiversity Observation Network (GEO BON) has developed the Essential Biodiversity Variables (EBVs), which are a set of variables aiming to capture different levels of biodiversity and are divided into 6 classes: genetic composition, species populations, species traits, community composition, ecosystem function, ecosystem structure. Specifically, the class of genetic composition includes genetic diversity and genetic differentiation or genetic distance; the class of species populations includes species distribution and abundances; the class of community composition includes community abundance and taxonomic/phylogenetic diversity^7^.

The advent of environmental DNA (eDNA) metabarcoding as a high-throughput tool for biodiversity assessment is certainly facilitating data collection for biodiversity monitoring.

Environmental DNA (eDNA) refers to DNA from an environmental sample and it is possible to identify two main ways to obtain this genetic material^8,9^. The first method involves DNA to be extracted from a bulk sample of organisms. The bulk sampling method has been applied to small planktonic organisms, but also to larger and multicellular species^10^. The second method uses traces of DNA collected by filtering water or using soil or sediment samples^11^. The sources of this DNA include cells, faeces, mucous, gametes, degrading cells or tissues and fragments resulting from predation^9^.

eDNA metabarcoding as a biodiversity assessment tool has already been applied in a variety of environments indicating its capability to unveil the macro-guilds used as ecological indicators as well as ecological communities structure^12–14^. Among ecological indicators, phytoplankton has been subjected to monitoring studies using eDNA metabarcoding in lakes and rivers^15–18^.

Phytoplankton is a valuable ecological indicator due to its adaptability to environmental variations, the effect of its carbon sequestration ability on climate regulation, and its role in primary productivity^19–22^. Therefore, phytoplankton surveys are particularly important in the current context of critical environmental changes. However, despite the importance of phytoplankton in aquatic communities, accurate classification of phytoplankton species is notoriously difficult due to the occurrence of cryptic sibling species and cell dimensions, and eDNA metabarcoding can be considered an asset for rapid surveying of phytoplanktonic communities, including taxa hard to identify with traditional methods.

In this study, we applied eDNA metabarcoding to assess, for the first time, the biodiversity of eukaryotic phytoplankton communities in a Mediterranean coastal lagoon protected under the European Natura 2000 Network. This work suggests the validity of eDNA studies in establishing the structure and spatial variation of phytoplankton communities in transitional water ecosystems, as well as to correlate the phytoplankton communities structure to abiotic drivers, including also evidence of genetic diversity for some phytoplankton genera.

## Materials and methods

### Study area

**Figure 1:**
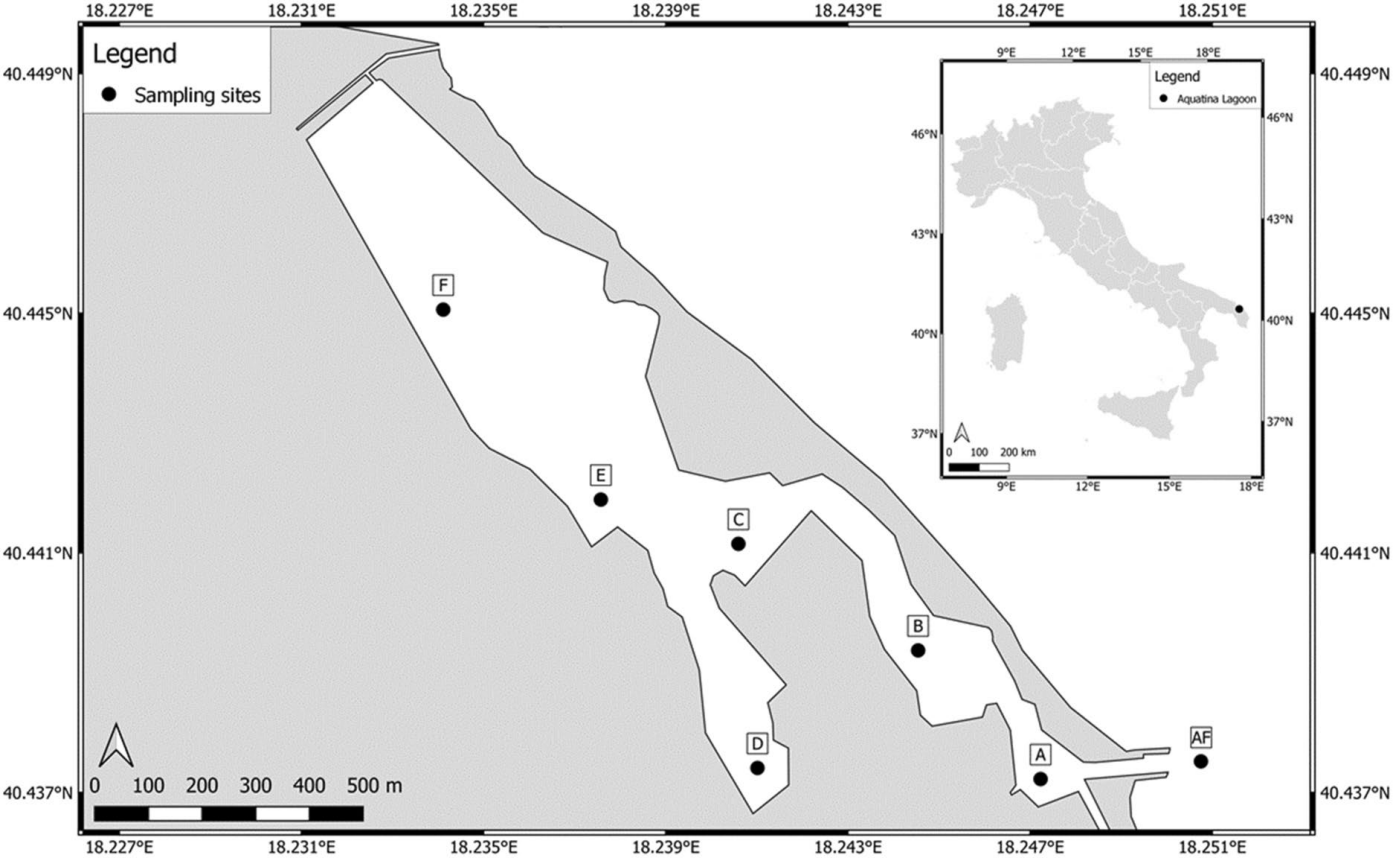
Map of the Aquatina Lagoon showing the location of the sampling sites. The map was generated using QGIS v3.28 (https://qgis.org).

The Aquatina Lagoon (40.442463°N–18.237675°E; Fig. 1) extends for 42 hectares, representing about 3% of the whole NATURA 2000 Site “Aquatina di Frigole”, in Southern Italy (IT9150003). The lagoon stretches for 2 km parallel to the dune cordon, with a maximum depth of approximately 1.5 m and a maximum tidal excursion, on an annual basis, of approximately 34 cm. It is subjected to saltwater input from the Adriatic Sea through a 15 m wide and 400 m long channel and a freshwater input through the lateral ramification of the Giammatteo canal on the northern boundary.

### Sampling and DNA extraction

On May 2021, we collected water samples in 7 different sites of the lagoon: (i) 3 outer sampling stations adjacent to the coastal dunes named A, B, and C; (ii) 3 inner sampling stations named D, E, and F; and (iii) 1 sampling station (named AF) at the end of the channel for the communication with the Adriatic Sea (Fig. 1). We collected 1 L of surface water in each station and transferred it to the Research Centre for Fisheries and Aquaculture of Aquatina di Frigole–University of Salento, Italy, located in the NATURA 2000 Site. To avoid filter clogging and reduce Polymerase Chain Reaction (PCR) inhibitors like particulate and humic substances, prefiltration was applied before filtration. Each water sample was first prefiltered in a 1.6 μm glass filter of 42.5 mm diameter (Whatman® glass microfiber filters, Grade GF/A), and then filtered in a 0.45 μm cellulose filter of 47 mm diameter (Advantec Mixed Cellulose Ester filters). The 0.45 μm filters were used to extract DNA through the DNeasy PowerWater Kit (QIAGEN, Germany) following the manufacturer’s protocol.

Abiotic parameters (Salinity, Temperature, pH, and Dissolved Oxygen) are routinely monitored in the Aquatina lagoon in correspondence with the 7 sampling sites using the multiparametric probe YSI ProQuatro® (xylem INC, USA).

### DNA amplification, NGS sequencing and bioinformatic analysis

The V4 region of 18S rRNA gene amplification was performed using the primers pair TAReuk454FWD1 and TAReukREV3^23^. The obtained amplicon is about 390 bp in length. The reaction was performed in a volume of 50 μL composed of 5 μL of 10× reaction buffer, 1 μL of MgCl2 (50 mM), 1 μL of dNTP mix (10 mM), 1 μL of each primer (10 mM), 10 ng DNA, 0.2 μL of Platinum Taq (5 U/μL) (Life Technologies, USA) and sterile water to reach a volume of 50 μL. The amplification program included the following phases: initial denaturation at 95 °C for 5 min, 30 cycles of denaturation (95 °C for 30″), annealing (50 °C for 30″), extension (72 °C for 30″) and a final extension at 72 °C for 10′. All PCR products were purified with a PureLink PCR purification kit (Invitrogen, Carlsbad, CA, USA). Celero™ DNA-Seq Library Preparation Kit cod. 0360A (Tecan, Männedorf, CH) was used for library preparation following the manufacturer’s instructions, without fragmenting the amplicons. Both input and final libraries were quantified by Qubit 2.0 Fluorometer (Invitrogen, Carlsbad, CA) and quality tested by Agilent 2100 Bioanalyzer High Sensitivity DNA assay (Agilent Technologies, Santa Clara, CA). Libraries were sequenced through the MiSeq instrument (Illumina, San Diego, CA) using 300 bp paired-end mode by IGATech, Udine, Italy. The generated FastQ files are deposited in GenBank (SRA) of NCBI with identification number PRJNA880489.

QIIME pipelines and the USEARCH algorithm (version 8.1.1756, 32-bit) were used for (i) chimaera filtering; (ii) grouping of replicate sequences, and (iv) OTU identification. The RDP classifier and the reference database SILVA 138 were used for taxonomic annotation. The resulting OTU list was filtered to contain only phytoplankton annotated OTUs.

### Statistical OTU diversity estimation

OTU richness has been calculated as the total number of OTUs per sampling site. OTU occurrence has been calculated as the percentage of OTUs occurring in the same number of sampling sites. All calculations have been done through the software Microsoft Excel. Bray–Curtis distance-based Redundancy Analysis (db-RDA) was performed by using the R Package Vegan. Response variables are represented by the occurrence of OTUs in each sampling site, while explanatory variables are represented by the mean abiotic parameter values registered in the Aquatina lagoon during routine surveys, specifically during the sampling period (Salinity, Temperature, Dissolved Oxygen, and pH). Two-way ANOVA was performed for significance values. The results are visualised as an RDA plot obtained using the R Package ggplot2.

### Neighbour-joining phylogenetic analysis

OTUs consensus sequences were multiple aligned using Clustal Omega 1.2.2 through the Geneious Prime alignment options, with settings automatically adjusted based on the number of sequences. The obtained multiple sequence alignment was used to construct the Neighbour-Joining tree. For the tree construction, Resampling Method was set on Bootstrap with 674,476 random seeds and 1000 replicates. The genetic distance model was set on Jukes-Cantor, with no outgroup. Tree Build Method was set on Neighbour-Joining.

## Results

### eDNA detects phytoplankton biodiversity in the lagoon

To analyse the biodiversity of phytoplankton communities characterizing the study area, we identified seven sampling sites along the entire lagoon. DNA was extracted from water samples of the selected sites, and a region of the 18S rRNA gene marker was amplified with primers spanning the V4 region of the gene. Illumina sequencing generated a total of about 3,000,000 raw 18S rRNA reads with an average of about 420,000 sequences per sample, of which 41% were retained after bioinformatic processing (Table S1). The Good’s coverage and Shannon Index rarefaction curve of filtered sequences indicated that the samples reached a plateau (Figs. S1, S2), and therefore sequencing depth was appropriate in all the samples.

**Figure 2:**
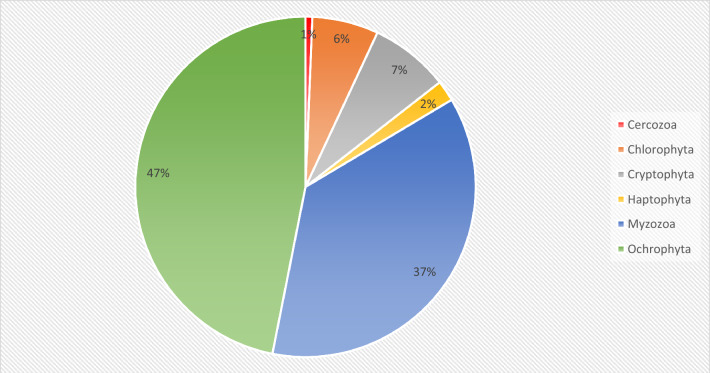
Total percentage of Phytoplankton OTUs at phyla level.

Following taxonomic assignment, only the OTUs annotated as phytoplankton taxa and classified beyond the D2 level of the SILVA 138 database were retained, resulting in a total of 2293 phytoplankton OTUs. This eDNA metabarcoding experiment detected a large fraction of phytoplankton groups (Table S2), of which 48.8% were assigned up to genus level and 9% up to species level. Analysing the biodiversity of phytoplankton OTUs, 47% of OTUs belong to the Ochrophyta phylum, 37% of OTUs belong to the Myzozoa phylum, 7% and 6% of OTUs belong to Cryptophyta and Chlorophyta phyla, respectively (Fig. 2). eDNA analysis detected OTUs annotated as nano- and pico-phytoplanktonic genera such as *Ostreococcus*, *Micromonas* and *Bolidomonas*.

**Figure 3:**
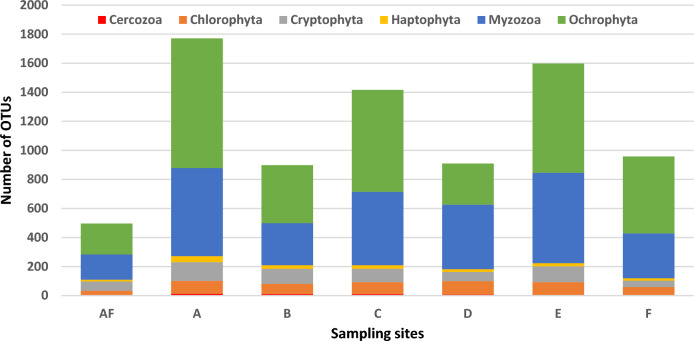
Number of OTUs at phyla level in each of the seven sampling sites.

Interestingly, eDNA from water samples, in our experimental conditions, was able to detect a wide fraction of phytoplankton taxa ranging over different phytoplankton size classes. In addition, both the total and relative numbers of OTUs belonging to the identified phyla present a specific and different distribution in the seven selected sites of the lagoon (Fig. 3).

### Phytoplankton OTU richness and occurrence across lagoon sites

To quantify the differences in phytoplankton OTU richness and composition, we compiled the OTU presence/absence matrix (Table S3). Based on this matrix, OTU richness and OTU occurrence were calculated.

OTU richness represents the number of phytoplankton OTUs observed per sampling site, revealing that sites A (n. of OTUs 1771), E (n. of OTUs 1599), and C (n. of OTUs 1417) host most of the OTU richness, site AF (n. of OTUs 496) appears to be the poorest, and sites B (n. of OTUs 899), D (n. of OTUs 909), and F (n. of OTUs 959) present intermediate values.

**Figure 4:**
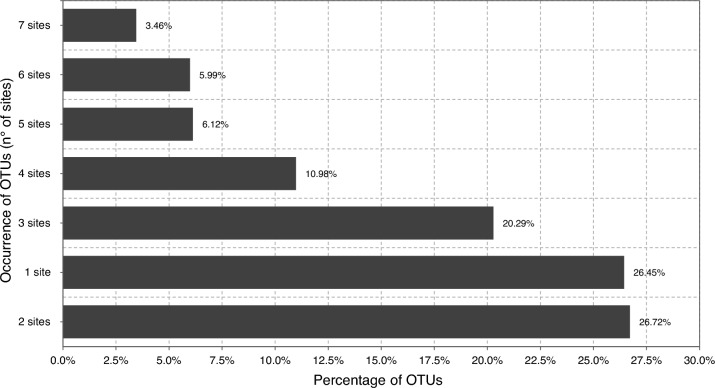
OTUs occurrence analysis calculated as the percentage of OTUs occurring in the same number of sampling sites.

The OTU occurrence has been calculated as the percentage of phytoplankton OTUs occurring in the same number of sampling sites and visualised as a bar plot (Fig. 4). The majority of OTUs (26.72%) occur in two sampling sites, while the minority of OTUs (3.46%) occur in all seven sampling sites.

### Abiotic parameters explain occurrence heterogeneity among lagoon sites

**Figure 5:**
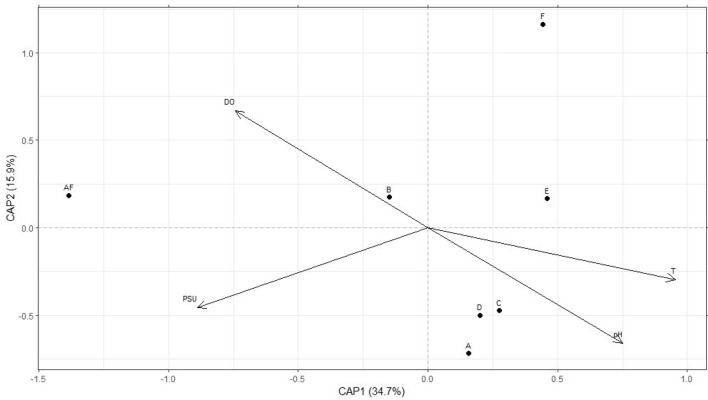
Bray–Curtis distance-based Redundancy Analysis (db-RDA) calculated to explain the differences in the phytoplankton OTUs distribution across the Aquatina di Frigole lagoon. PSU = Salinity (PSU); T = Temperature (°C); DO = Dissolved oxygen (mg/L). Black dots and letters indicate the seven sampling sites.

To infer the dissimilarity between sampling sites and correlate it to the main abiotic drivers that characterize the environment under study through a constrained ordination, we performed a Bray–Curtis distance-based Redundancy Analysis (db-RDA). All loading and score values are summarised in Table S4. The most commonly routinely surveyed environmental parameters have been used as explanatory variables (independent variables): Temperature (T), Salinity (PSU), Dissolved Oxygen (DO) and pH (Fig. 5). Specifically, these environmental parameters are particularly relevant for transitional water ecosystems, in which they describe their heterogeneity. The explained variables (dependent variables) are represented by the OTUs composition in each sampling site. According to the *p*-values calculated for each of the abiotic parameters, it results that salinity (*p* < 0.01) and temperature (*p* < 0.01) are the main explanatory variables for the phytoplankton OTUs distribution across the sampling sites, while pH (*p* < 0.05) and especially DO (*p* < 0.1) do not seem to affect it.

The biplot of this analysis displays sites A, C, and D grouped, while sites AF, B, E, and F do not show any grouping pattern. This result reflects the similar environmental conditions occurring in sites A, C, and D, where comparable levels of the considered environmental variables have been registered. The proximity of these three sites in the biplot also indicates similarities in the phytoplankton OTUs composition across these sites. At the same time, the four sites far apart in the biplot represent areas with distinct environmental differences, influencing the phytoplankton OTUs distribution in a way that separates them apart and from the other three sites.

### Exploring genetic diversity across the lagoon: the case of *Tetraselmis* genus

**Figure 6:**
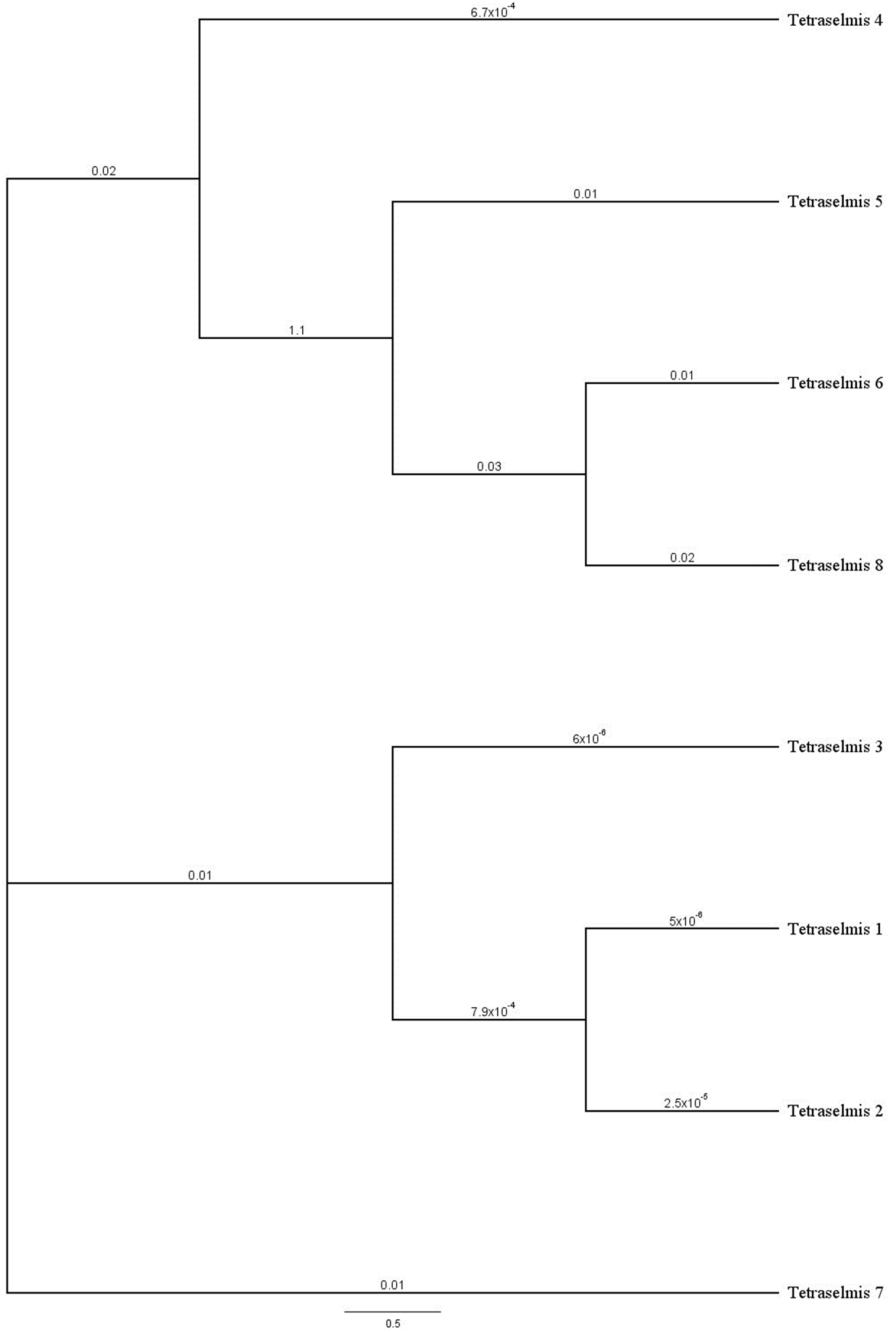
Neighbour-joining phylogenetic tree (Jukes-Cantor genetic distance model with 674,476 random seed and 1000 bootstrap replicates) of OTUs of *Tetraselmis* genus for V4 region of 18S marker. The Clustal omega multiple sequence alignment consists of about 390 bp region. Branch labels indicate substitutions per site.

The analysis of the presence/absence matrix of OTUs highlighted that different OTUs assigned to the same taxon exhibited a different distribution across sampling sites. Specifically, we focused on multiple OTUs assigned to the *Tetraselmis* genus because the relative and different OTUs presented a clearly different distribution among sampling sites. The bioinformatics analysis recovered 8 OTUs assigned to the *Tetraselmis* genus highly differently occurring across the sampling sites (Table S5). 2 OTUs occurred only in the marine site AF, 4 OTUs presented wide occurrence across sampling sites, and 2 OTUs presented a peculiar occurrence in two sampling sites. We analysed the genetic distance, calculated as nucleotide substitutions in a phylogenetic tree, to inspect if the differences in the spatial distribution reflect genetic variations (Fig. 6).

Interestingly, the OTUs present only in the marine site AF (*Tetraselmis* 6 and 8) clustered and separated from OTUs widely distributed across the sampling sites, especially *Tetraselmis* 1, 2, 3, but significantly also from Tetraselmis 5.

This analysis suggests that the different spatial distribution of OTUs, at least for the *Tetraselmis* genus, presents a relation with the genetic background.

## Discussion

Coastal lagoons provide valuable ecosystem services and are suitable areas for biodiversity conservation^24^. However, they are sensitive to climatic and anthropogenic pressures, which are compromising their ecological integrity^25, 26^. Among the ecological indicators, eukaryotic phytoplankton is known to exhibit remarkable species richness in transitional waters^27^. However, molecular phytoplankton surveys are still in their infancy in these environments, where they are only limited to the detection of harmful algal blooms^28^. In this research, we conducted eDNA metabarcoding with the 18S rRNA gene marker in a protected Mediterranean coastal lagoon. We selected 18S as a gene marker because it is a largely represented barcode gene for phytoplankton in Mediterranean ecosystems^29^. We assessed an experimental plan aimed to overcome specific issues related to lagoon ecosystems such as water turbidity and abundance of humic substances. We extracted the DNA present in the water column, recovering phytoplankton biodiversity ranging over all the phytoplankton size classes. In general, for planktonic organisms has been applied the bulk sample approach. We used the eDNA metabarcoding method and the results highlight the identification of a wide fraction of nanophytoplankton and microphytoplankton, representing about 80% of OTUs. The high percentage of OTUs belonging to these phytoplanktonic size classes could reflect the different coverage of phytoplankton species in the DNA barcode reference databases.

In this study, eDNA also detected significant correlations between phytoplankton OTUs occurrence and lagoon abiotic components. Specifically, the significant environmental explanatory variables were salinity and temperature (*p*-values < 0.01), displaying a high difference between site AF, located at the interface between the sea and the lagoon, characterized by high salinity and low temperature, site F, located in proximity to the freshwater input, characterized by low salinity and higher temperature, and the other sites, characterized by a greater intermediate homogeneity. The specific pattern observed in the db-RDA biplot shows that similar environmental conditions correspond to similarities in the phytoplankton community composition, while distinct environmental differences reflect in separated communities. These results confirm that environmental heterogeneity can represent the spatial factor playing a key role in influencing the phytoplankton community structure in transitional water ecosystems^30^.

Different OTUs were taxonomically assigned to the *Tetraselmis* genus. The absence of species assignment is probably related to the absence of 18S DNA barcodes for Mediterranean *Tetraselmis* species in the reference databases. OTUs belonging to the *Tetraselmis* genus and highly differently distributed across the sampling sites were analysed for the genetic distances. The results outlined that genetic backgrounds correlate with specific spatial distribution. Although the analysis is limited to a short fragment of a conserved gene, the results suggest that sequencing data from eDNA amplicons could be useful to inspect directional genetic variations.

A high genetic variation is important for the ability of phytoplanktonic assemblages to be extremely viable in highly heterogeneous environments such as coastal lagoons, which are subjected to strong variations driven by extrinsic factors such as climate and tides, other than by intrinsic variability given by abiotic heterogeneity.

Overall, this work demonstrates that eDNA metabarcoding is a valuable tool for rapidly surveying the phytoplanktonic communities and describing their spatial distribution in transitional water ecosystems.

### Supplementary Information

Supplementary Information.

Supplementary Table S2.

Supplementary Table S3.

Supplementary Table S4.

Supplementary Table S5.

## Data Availability

The FASTQ files are deposited in GenBank (SRA) of NCBI with identification number PRJNA880489.
