# Supplementary material for: Environmental DNA detects biodiversity and ecological features of phytoplankton communities in Mediterranean transitional waters
Source: Sci Rep. 2023 Sep 14;13:15192. doi: 10.1038/s41598-023-42389-3 (PMC10502138; doi:10.1038/s41598-023-42389-3)
Supplement: Supplementary file 1 — Supplementary Information. [file 41598_2023_42389_MOESM1_ESM.docx]

**
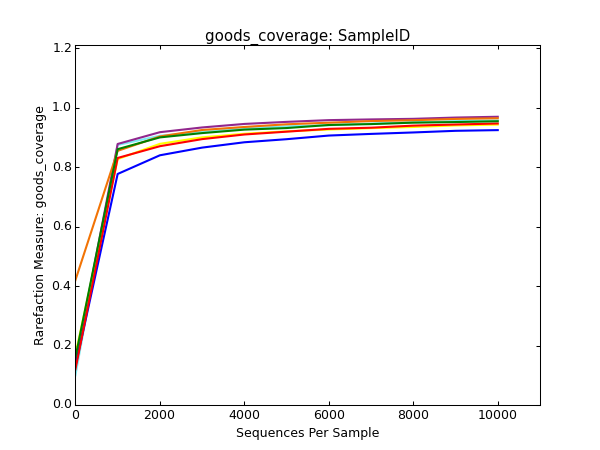
**

Figure S1. Good’s coverage rarefaction curve in the seven samples. Red= site AF, Blue=Site A, Orange= site B, Green = site C, Purple= site D, yellow= site E, light blue= site F

**
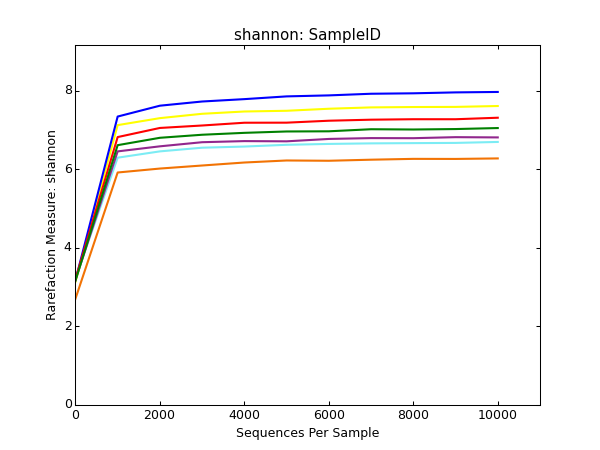
**

Figure S2. Shannon rarefaction curve in the seven samples. Red= site AF, Blue=Site A, Orange= site B, Green = site C, Purple= site D, yellow= site E, light blue= site F

| **Numbers** | **AF** | **A** | **B** | **C** | **D** | **E** | **F** |
| --- | --- | --- | --- | --- | --- | --- | --- |
| raw reads | 335026 | 430288 | 449824 | 427260 | 396706 | 503036 | 422956 |
| reads after bioinformatic processing | 133422 | 164371 | 194741 | 178019 | 167200 | 205578 | 176453 |
| annotated eukaryotes reads | 123546 | 161488 | 194308 | 177777 | 165950 | 204827 | 174746 |
| annotated eukaryotes beyond D2 level | 107749 | 157442 | 192053 | 176733 | 163428 | 202082 | 170405 |
| Phytoplankton reads | 37650 | 65418 | 64769 | 77565 | 103130 | 93936 | 78730 |
| Phytoplankton OTUs | 496 | 1771 | 898 | 1416 | 909 | 1598 | 958 |

Table S1. Total number of reads for each sample
