# Supplementary material for: Environmental DNA detects biodiversity and ecological features of phytoplankton communities in Mediterranean transitional waters
Source: Sci Rep. 2023 Sep 14;13:15192. doi: 10.1038/s41598-023-42389-3 (PMC10502138; doi:10.1038/s41598-023-42389-3)
Supplement: Supplementary file 2 — Supplementary Table S2. [file 41598_2023_42389_MOESM2_ESM.docx]

| **Phylum** | **Infraphylum** | **Class** | **Order** | **Family** | **Genus** | **Species** |
| --- | --- | --- | --- | --- | --- | --- |
| Cercozoa |  | Chlorarachniophyceae | Chlorachniida | Chlorarachniaceae | *Chlorarachnion* |  |
| Cercozoa |  | Chlorarachniophyceae | Chlorachniida | Chlorarachniaceae | *Chlorarachnion* | *Chlorarachnion reptans* |
| Cercozoa |  | Chlorarachniophyceae | Chlorachniida | Chlorarachniaceae | *Amorphochlora* |  |
| Cercozoa |  | Chlorarachniophyceae | Chlorachniida | Chlorarachniaceae | *Bigelowiella* |  |
| Cercozoa |  | Imbricatea | Euglyphida | Paulinellidae | *Paulinella* |  |
| Cercozoa |  | Imbricatea | Spongomonadida | Spongomonadidae | *Spongomonas* |  |
| Chlorophyta |  | Mamiellophyceae | Mamiellales | Bathycoccaceae | *Bathycoccus* |  |
| Chlorophyta |  | Mamiellophyceae | Mamiellales | Bathycoccaceae | *Ostreococcus* |  |
| Chlorophyta |  | Chlorodendrophyceae | Chlorodendrales | Chlorodendraceae | *Tetraselmis* |  |
| Chlorophyta |  | Mamiellophyceae | Dolichomastigales | Dolichomastigaceae | *Dolichomastix* | *Dolichomastix tenuilepis* |
| Chlorophyta |  | Mamiellophyceae | Dolichomastigales | Dolichomastigaceae | *Crustomastix* |  |
| Chlorophyta |  | Mamiellophyceae | Mamiellales | Mamiellaceae | *Micromonas* | *Micromonas pusilla* |
| Chlorophyta |  | Mamiellophyceae | Mamiellales | Mamiellaceae | *Micromonas* |  |
| Chlorophyta |  | Ulvophyceae | Ulvales | Phaeophilaceae | *Phaeophila* | *Phaeophila dendroides* |
| Chlorophyta |  | Pyramimonadophyceae | Prasinococcales | Prasinococcaceae | *Prasinoderma* |  |
| Chlorophyta |  | Mamiellophyceae | Mamiellales | Prasinophyceae | *Mamiella* |  |
| Chlorophyta |  | Ulvophyceae | Ulvales | Ulvaceae | *Ochlochaete* | *Ochlochaete hystrix* |
| Chlorophyta |  | Chlorodendrophyceae | Chlorodendrales |  |  |  |
| Chlorophyta |  | Mamiellophyceae |  |  |  |  |
| Chlorophyta |  | Pyramimonadophyceae | Pyramimonadales |  |  |  |
| Chlorophyta |  | Trebouxiophyceae |  |  |  |  |
| Cryptophyta |  | Cryptophyceae | Cryptomonadales | Chroomonadaceae | *Hemiselmis* | *Hemiselmis viriscens* |
| Cryptophyta |  | Cryptophyceae | Cryptomonadales | Chroomonadaceae | *Hemiselmis* |  |
| Cryptophyta |  | Cryptophyceae | Cryptomonadales | Geminigeraceae | *Proteomonas* |  |
| Cryptophyta |  | Cryptophyceae | Cryptomonadales | Geminigeraceae | *Proteomonas* | *Proteomonas sulcata* |
| Cryptophyta |  | Cryptophyceae | Cryptomonadales | Geminigeraceae | *Teleaulax* |  |
| Cryptophyta |  | Cryptophyceae | Cryptomonadales | Hemiselmidaceae | *Chroomonas* |  |
| Cryptophyta |  | Cryptophyceae | Cryptomonadales | Pyrenomonadaceae | *Rhinomonas* | *Rhinomonas nottbecki* |
| Cryptophyta |  | Telonemea | Telonemida |  | *Telonema* |  |
| Cryptophyta |  | Cryptophyceae | Cryptomonadales |  |  |  |
| Haptophyta |  | Pavlovophyceae | Pavlovales | Pavlovaceae | *Pavlova* |  |
| Haptophyta |  | Prymnesiophyceae | Prymnesiales | Prymnesiaceae | *Chrysocampanula/Chrisocromulina* |  |
| Haptophyta |  | Prymnesiophyceae | Prymnesiales | Prymnesiaceae |  |  |
| Haptophyta |  | Pavlovophyceae | Pavlovales |  |  |  |
| Haptophyta |  | Prymnesiophyceae | Coccolithales |  |  |  |
| Haptophyta |  | Prymnesiophyceae | Phaeocystis |  |  |  |
| Haptophyta |  | Prymnesiophyceae | Prymnesiales |  |  |  |
| Haptophyta |  | Prymnesiophyceae |  |  |  |  |
| Myzozoa | Dinoflagellata | Dinophyceae | Syndiniales | Amoebophryaceae | *Amoebophrya* |  |
| Myzozoa | Dinoflagellata | Dynophyceae | Gonyaulacales | Amphidomataceae | *Azadinium* |  |
| Myzozoa | Dinoflagellata | Dynophyceae | Suessiales | Biecheleriaceae | *Biecheleria* |  |
| Myzozoa | Sporozoa | Dynophyceae | Eucoccidiorida | Eimeriidae | *Margolisiella* |  |
| Myzozoa | Dinoflagellata | Dynophyceae | Gonyaulacales | Gonyaulacaceae | *Gonyaulax* | *Gonyaulax fragilis* |
| Myzozoa | Dinoflagellata | Dynophyceae | Gonyaulacales | Gonyaulacaceae | *Gonyaulax* | *Gonyaulax spinifera* |
| Myzozoa | Dinoflagellata | Dynophyceae | Gonyaulacales | Gonyaulacaceae | *Gonyaulax* |  |
| Myzozoa | Dinoflagellata | Dynophyceae | Gymnodiniales | Gymnodiniaceae | *Akashiwo* |  |
| Myzozoa | Dinoflagellata | Dynophyceae | Gymnodiniales | Gymnodiniaceae | *Cochlodinium* |  |
| Myzozoa | Dinoflagellata | Dynophyceae | Gymnodiniales | Gymnodiniaceae | *Paragymnodinium* |  |
| Myzozoa | Dinoflagellata | Dynophyceae | Gymnodiniales | Gymnodiniaceae | *Polykrikos* | *Polykrikos kofoidii* |
| Myzozoa | Dinoflagellata | Dynophyceae | Gymnodiniales | Gymnodiniaceae | *Polykrikos* |  |
| Myzozoa | Dinoflagellata | Dynophyceae | Gymnodiniales | Gymnodiniaceae | *Gymnodinium* |  |
| Myzozoa | Dinoflagellata | Dynophyceae | Amphidiniales | Amphidiniaceae | *Amphidinium* | *Amphidinium steinii* |
| Myzozoa | Dinoflagellata | Dynophyceae | Peridiniales | Peridiniaceae | *Bysmatrum* | *Bysmatrum gregarium* |
| Myzozoa | Dinoflagellata | Dynophyceae | Amphidiniales | Amphidiniaceae | *Amphidinium* |  |
| Myzozoa | Dinoflagellata | Dynophyceae | Gymnodiniales | Gymnodiniaceae | *Gyrodinium* |  |
| Myzozoa | Dinoflagellata | Gymnodiniphycidae |  |  |  |  |
| Myzozoa | Dinoflagellata | Dinophyceae | Dinophyceae incertae sedis |  | *Psammosa* | *Psammosa atlantica* |
| Myzozoa | Dinoflagellata | Dynophyceae | Peridiniales | Kryptoperidiniaceae | *Durinskia* |  |
| Myzozoa | Dinoflagellata | Dynophyceae | Peridiniales | Lessardiaceae | *Lessardia* |  |
| Myzozoa | Dinoflagellata | Dynophyceae | Gonyaulacales | Ostreopsidaceae | *Alexandrium* | *Alexandrium andersonii* |
| Myzozoa | Dinoflagellata | Dynophyceae | Gonyaulacales | Ostreopsidaceae | *Alexandrium* | *Alexandrium margalefii* |
| Myzozoa | Dinoflagellata | Dynophyceae | Gonyaulacales | Ostreopsidaceae | *Alexandrium* |  |
| Myzozoa | Dinoflagellata | Dinophyceae | Oxyrrhinales | Oxyrrhinaceae | *Oxyrrhis* |  |
| Myzozoa | Dinoflagellata | Dynophyceae | Peridiniales | Peridiniaceae | *Peridinium* |  |
| Myzozoa | Dinoflagellata | Dynophyceae | Peridiniales | Peridiniaceae | *Scrippsiella* |  |
| Myzozoa | Dinoflagellata | Dinophyceae | Perkinsida | Perkinsidae |  |  |
| Myzozoa | Dinoflagellata | Dinophyceae | Perkinsida | Perkinsidae |  |  |
| Myzozoa |  | Perkinsea | Perkinsida | Parviluciferaceae | *Snorkelia* | *Snorkelia prorocentri* |
| Myzozoa | Dinoflagellata | Dinophyceae | Perkinsida | Perkinsidae | *Parvilucifera* |  |
| Myzozoa | Dinoflagellata | Dynophyceae | Prorocentrales | Prorocentraceae | *Prorocentrum* |  |
| Myzozoa | Dinoflagellata | Dynophyceae | Eugregarinorida | Selenidiidae | *Selenidium* |  |
| Myzozoa | Dinoflagellata | Dinophyceae | Suessiales | Suessiaceae |  |  |
| Myzozoa | Dinoflagellata | Dynophyceae | Suessiales | Symbiodiniaceae | *Symbiodinium* |  |
| Myzozoa | Dinoflagellata | Dinophyceae | Thoracosphaerales | Thoracosphaeraceae |  |  |
| Myzozoa | Dinoflagellata | Dinophyceae | Colpodellida |  |  |  |
| Myzozoa | Dinoflagellata | Dinophyceae | Dinophyceae |  |  |  |
| Myzozoa | Dinoflagellata | Dinophyceae | Gonyaulacales |  |  |  |
| Myzozoa | Dinoflagellata | Dinophyceae | Peridiniales |  |  |  |
| Myzozoa | Dinoflagellata | Dinophyceae | Syndiniales |  |  |  |
| Myzozoa | Dinoflagellata | Dinophyceae |  |  |  |  |
| Myzozoa | Protalveolata |  |  |  |  |  |
| Myzozoa | Dinoflagellata | Dynophyceae | Syndiniales Group II |  |  |  |
| Ochrophyta |  | Bacillariophyceae | Achnanthales | Achnanthidiaceae | *Planothidium* | *Planothidium lanceolatum* |
| Ochrophyta |  | Bacillariophyceae | Bacillariales | Bacillariaceae | *Cylindrotheca* | *Cylindrotheca closterium* |
| Ochrophyta |  | Bacillariophyceae | Bacillariales | Bacillariaceae | *Fragilariopsis* |  |
| Ochrophyta |  | Bacillariophyceae | Bacillariales | Bacillariaceae | *Nitzschia* |  |
| Ochrophyta |  | Bacillariophyceae | Bacillariales | Bacillariaceae | *Pseudo-nitzschia* |  |
| Ochrophyta |  | Bacillariophyceae | Naviculales | Berkeleyaceae | *Berkeleya* | *Berkeleya hyalina* |
| Ochrophyta |  | Bolidophyceae | Bolidomonadales | Bolidomonadaceae | *Bolidomonas* |  |
| Ochrophyta |  | Bacillariophyceae | Thalassiophysales | Catenulaceae | *Undatella* |  |
| Ochrophyta |  | Bacillariophyceae | Achnanthales | Cocconeis | *Cocconeis* |  |
| Ochrophyta |  | Chrysophyceae | Ochromonadales | Dinobryaceae | *Epipyxis* |  |
| Ochrophyta |  | Bacillariophyceae | Fragilariales | Fragilariaceae | *Synedra* | *Synedra bacillaris* |
| Ochrophyta |  | Bacillariophyceae | Fragilariales | Fragilariaceae | *Synedra* |  |
| Ochrophyta |  | Bacillariophyceae | Fragilariales | Grammatophoraceae | *Hyalosira* | *Hyalosira delicatula* |
| Ochrophyta |  | Bacillariophyceae | Fragilariales | Licmophoraceae | *Licmophora* |  |
| Ochrophyta |  | Bacillariophyceae | Chaetocerotanae incertae sedis | Mediophyceae | *Chaetoceros* |  |
| Ochrophyta |  | Bacillariophyceae | Melosirales | Melosiraceae | *Melosira* |  |
| Ochrophyta |  | Bacillariophyceae | Naviculales | Naviculaceae | *Fistulifera* |  |
| Ochrophyta |  | Bacillariophyceae | Naviculales | Naviculaceae | *Navicula* |  |
| Ochrophyta |  | Chrysophyceae | Ochromonadales | Ochromonadaceae | *Ochromonas* |  |
| Ochrophyta |  | Chrysophyceae |  | P34.48 |  |  |
| Ochrophyta |  | Chrysophyceae | Ochromonadales | Paraphysomonadaceae | *Paraphysomonas* |  |
| Ochrophyta |  | Dictyochophyceae | Pedinellales | Pedinellaceae | *Ciliophrys* | *Ciliophrys infusionum* |
| Ochrophyta |  | Dictyochophyceae | Pedinellales | Pedinellaceae | *Pseudopedinella* |  |
| Ochrophyta |  | Dictyochophyceae | Pedinellales | Pedinellaceae | *Pteridomonas* |  |
| Ochrophyta |  | Bacillariophyceae | Bacillariophyta incertae sedis | Phaeodactylaceae | *Phaeodactylum* |  |
| Ochrophyta |  | Picophagophyceae | Picophagales | Picophagaceae | *Picophagus* | *Picophagus flagellatus* |
| Ochrophyta |  | Bacillariophyceae | Rhaponeidales | Rhaphoneidaceae | *Rhaphoneis* |  |
| Ochrophyta |  | Dictyochophyceae | Rhizochromulinales | Rhizochromulinaceae | *Rhizochromulina* |  |
| Ochrophyta |  | Bacillariophyceae | Cymbellales | Rhoicospheniaceae | *Rhoicosphenia* |  |
| Ochrophyta |  | Bacillariophyceae | Naviculales | Sellaphoraceae | *Sellaphora* |  |
| Ochrophyta |  | Bacillariophyceae | Naviculales | Stauroneidaceae | *Stauroneis* | *Stauroneis acuta* |
| Ochrophyta |  | Bacillariophyceae | Naviculales | Stauroneidaceae | *Stauroneis* |  |
| Ochrophyta |  | Bacillariophyceae | Fragilariales | Staurosiraceae | *Opephora* |  |
| Ochrophyta |  | Bacillariophyceae | Striatellales | Striatellaceae | *Striatella* | *Striatella unipunctata* |
| Ochrophyta |  | Chrysophyceae | Hibberdiales | Stylococcaceae | *Chrysopodocystis* | *Chrysopodocystis socialis* |
| Ochrophyta |  | Bacillariophyceae | Surirellales | Surirellaceae | *Campylodiscus* |  |
| Ochrophyta |  | Synchromophyceae | Synchromales | Synchromaceae | *Synchroma* | *Synchroma pusillum* |
| Ochrophyta |  | Bacillariophyceae | Thalassiophysales | Thalassiophysales | *Amphora* | *Amphora eunotia* |
| Ochrophyta |  | Bacillariophyceae | Thalassiophysales | Thalassiophysales | *Amphora* | *Amphora montana* |
| Ochrophyta |  | Bacillariophyceae | Thalassiophysales | Thalassiophysales | *Amphora* |  |
| Ochrophyta |  | Bacillariophyceae | Thalassiosirales | Thalassiosiraceae | *Thalassiosira* |  |
| Ochrophyta |  | Bacillariophyceae | Toxariales | Toxariaceae | *Toxarium* |  |
| Ochrophyta |  | Bacillariophyceae | Fragilariales | Ulnariaceae | *Hyalosynedra* |  |
| Ochrophyta |  | Bacillariophyceae | Fragilariales |  |  |  |
| Ochrophyta |  | Bacillariophyceae |  |  |  |  |
| Ochrophyta |  | Chrysophyceae | Ochromonadales |  |  |  |
| Ochrophyta |  | Chrysophyceae |  |  |  |  |
| Ochrophyta |  | Dictyochophyceae | Pedinellales |  |  |  |
| Ochrophyta |  | Dictyochophyceae | Pinguiochrysidales |  |  |  |
| Ochrophyta |  | Dictyochophyceae |  |  |  |  |

**TABLE S1**. List of phytoplankton taxonomic assignment of OTUs
