# Supplementary material for: Environmental DNA detects biodiversity and ecological features of phytoplankton communities in Mediterranean transitional waters
Source: Sci Rep. 2023 Sep 14;13:15192. doi: 10.1038/s41598-023-42389-3 (PMC10502138; doi:10.1038/s41598-023-42389-3)
Supplement: Supplementary file 3 — Supplementary Table S3. [file 41598_2023_42389_MOESM3_ESM.docx]

**Table S3**. Presence-Absence phytoplankton OTUs matrix in the seven sampling sites. Green stands for presence, while red stands for absence.

|  | AF | A | B | C | D | E | F |
| --- | --- | --- | --- | --- | --- | --- | --- |
| U40920.1.1796 | 0 | 1 | 1 | 1 | 1 | 1 | 1 |
| New.CleanUp.ReferenceOTU10048 | 0 | 1 | 1 | 1 | 1 | 1 | 1 |
| New.CleanUp.ReferenceOTU16993 | 0 | 1 | 0 | 0 | 0 | 0 | 0 |
| New.CleanUp.ReferenceOTU17816 | 0 | 1 | 0 | 0 | 1 | 0 | 1 |
| New.CleanUp.ReferenceOTU47679 | 0 | 1 | 0 | 0 | 0 | 0 | 0 |
| New.CleanUp.ReferenceOTU51793 | 0 | 1 | 1 | 1 | 1 | 1 | 1 |
| New.CleanUp.ReferenceOTU59483 | 0 | 1 | 1 | 1 | 1 | 1 | 1 |
| AY425321.1.1750 | 0 | 0 | 0 | 0 | 1 | 0 | 1 |
| New.CleanUp.ReferenceOTU2446 | 0 | 0 | 0 | 0 | 1 | 0 | 1 |
| New.CleanUp.ReferenceOTU69262 | 0 | 1 | 1 | 1 | 0 | 1 | 0 |
| New.CleanUp.ReferenceOTU72897 | 0 | 1 | 1 | 1 | 0 | 1 | 0 |
| New.CleanUp.ReferenceOTU59536 | 0 | 0 | 0 | 0 | 0 | 1 | 0 |
| New.CleanUp.ReferenceOTU69272 | 0 | 0 | 0 | 0 | 0 | 1 | 0 |
| KJ762954.1.1780 | 0 | 1 | 1 | 1 | 1 | 1 | 0 |
| New.CleanUp.ReferenceOTU5842 | 0 | 1 | 0 | 0 | 0 | 1 | 0 |
| New.CleanUp.ReferenceOTU8427 | 0 | 0 | 0 | 0 | 0 | 1 | 0 |
| New.CleanUp.ReferenceOTU11274 | 0 | 0 | 0 | 0 | 0 | 1 | 0 |
| New.CleanUp.ReferenceOTU13566 | 0 | 1 | 0 | 0 | 0 | 1 | 0 |
| New.CleanUp.ReferenceOTU32131 | 0 | 1 | 1 | 1 | 1 | 1 | 0 |
| New.CleanUp.ReferenceOTU50465 | 0 | 1 | 1 | 0 | 1 | 1 | 0 |
| New.CleanUp.ReferenceOTU52132 | 0 | 0 | 0 | 0 | 0 | 1 | 0 |
| New.CleanUp.ReferenceOTU63371 | 0 | 0 | 0 | 1 | 1 | 1 | 0 |
| New.CleanUp.ReferenceOTU74596 | 0 | 0 | 0 | 0 | 1 | 1 | 0 |
| EF527029.1.1419 | 0 | 1 | 1 | 1 | 1 | 1 | 0 |
| New.CleanUp.ReferenceOTU64522 | 0 | 1 | 1 | 1 | 1 | 1 | 0 |
| KJ763648.1.1765 | 0 | 1 | 1 | 1 | 1 | 1 | 1 |
| New.CleanUp.ReferenceOTU79662 | 0 | 0 | 1 | 1 | 1 | 0 | 0 |
| New.CleanUp.ReferenceOTU22452 | 0 | 0 | 0 | 0 | 1 | 1 | 0 |
| New.CleanUp.ReferenceOTU33601 | 0 | 1 | 0 | 0 | 0 | 0 | 0 |
| New.CleanUp.ReferenceOTU59825 | 0 | 1 | 0 | 0 | 0 | 0 | 0 |
| New.CleanUp.ReferenceOTU61795 | 0 | 0 | 0 | 0 | 1 | 1 | 0 |
| New.CleanUp.ReferenceOTU72063 | 0 | 1 | 0 | 0 | 0 | 0 | 0 |
| AF145226.1.1782 | 0 | 0 | 1 | 1 | 0 | 1 | 1 |
| New.ReferenceOTU316 | 1 | 1 | 1 | 1 | 1 | 1 | 1 |
| New.CleanUp.ReferenceOTU2102 | 0 | 0 | 0 | 0 | 0 | 1 | 0 |
| New.CleanUp.ReferenceOTU35576 | 1 | 0 | 0 | 0 | 0 | 0 | 0 |
| New.CleanUp.ReferenceOTU35864 | 1 | 1 | 1 | 1 | 1 | 1 | 1 |
| New.CleanUp.ReferenceOTU40992 | 0 | 0 | 0 | 0 | 0 | 1 | 0 |
| New.CleanUp.ReferenceOTU41048 | 1 | 0 | 0 | 0 | 0 | 0 | 0 |
| New.CleanUp.ReferenceOTU45850 | 1 | 1 | 1 | 1 | 1 | 1 | 1 |
| KU743546.1.2212 | 0 | 1 | 1 | 0 | 0 | 1 | 0 |
| New.CleanUp.ReferenceOTU40755 | 0 | 1 | 1 | 0 | 0 | 1 | 0 |
| KJ763039.1.1793 | 0 | 0 | 1 | 0 | 0 | 1 | 0 |
| New.CleanUp.ReferenceOTU26300 | 0 | 1 | 0 | 1 | 0 | 0 | 0 |
| KJ763000.1.1766 | 0 | 0 | 1 | 0 | 0 | 0 | 0 |
| New.CleanUp.ReferenceOTU53528 | 0 | 0 | 0 | 0 | 1 | 0 | 0 |
| New.CleanUp.ReferenceOTU72874 | 0 | 0 | 1 | 0 | 0 | 0 | 0 |
| New.CleanUp.ReferenceOTU75518 | 0 | 0 | 0 | 0 | 1 | 0 | 0 |
| JF791090.1.1777 | 0 | 1 | 1 | 1 | 0 | 0 | 0 |
| New.CleanUp.ReferenceOTU16860 | 0 | 0 | 1 | 0 | 0 | 0 | 0 |
| New.CleanUp.ReferenceOTU48814 | 0 | 1 | 1 | 1 | 0 | 0 | 0 |
| JF791091.1.1777 | 0 | 0 | 1 | 0 | 0 | 0 | 0 |
| KJ763409.1.1791 | 0 | 1 | 1 | 1 | 0 | 1 | 1 |
| New.CleanUp.ReferenceOTU429 | 0 | 1 | 1 | 1 | 0 | 1 | 1 |
| New.CleanUp.ReferenceOTU4793 | 0 | 1 | 0 | 0 | 0 | 0 | 0 |
| HM749950.1.1540 | 1 | 0 | 1 | 1 | 1 | 1 | 1 |
| EF527097.1.1338 | 1 | 1 | 1 | 1 | 1 | 1 | 1 |
| New.ReferenceOTU340 | 1 | 1 | 1 | 1 | 1 | 1 | 1 |
| New.ReferenceOTU59 | 1 | 1 | 1 | 1 | 1 | 1 | 1 |
| New.CleanUp.ReferenceOTU77928 | 1 | 1 | 1 | 1 | 1 | 1 | 1 |
| KJ759496.1.1802 | 0 | 1 | 1 | 1 | 0 | 1 | 1 |
| KJ759752.1.1800 | 1 | 1 | 1 | 1 | 1 | 1 | 1 |
| New.CleanUp.ReferenceOTU3129 | 0 | 0 | 1 | 1 | 0 | 1 | 0 |
| New.CleanUp.ReferenceOTU3516 | 1 | 0 | 0 | 1 | 0 | 0 | 1 |
| New.CleanUp.ReferenceOTU10127 | 0 | 0 | 0 | 0 | 0 | 0 | 1 |
| New.CleanUp.ReferenceOTU10803 | 1 | 1 | 1 | 1 | 1 | 1 | 1 |
| New.CleanUp.ReferenceOTU13392 | 0 | 1 | 0 | 0 | 0 | 1 | 0 |
| New.CleanUp.ReferenceOTU14920 | 1 | 1 | 1 | 1 | 1 | 1 | 1 |
| New.CleanUp.ReferenceOTU30554 | 1 | 1 | 1 | 1 | 1 | 1 | 1 |
| New.CleanUp.ReferenceOTU40200 | 0 | 1 | 1 | 1 | 0 | 1 | 1 |
| New.CleanUp.ReferenceOTU51523 | 0 | 0 | 1 | 1 | 1 | 0 | 0 |
| New.CleanUp.ReferenceOTU57770 | 0 | 0 | 0 | 0 | 0 | 0 | 1 |
| New.CleanUp.ReferenceOTU57973 | 1 | 1 | 1 | 1 | 1 | 1 | 1 |
| New.CleanUp.ReferenceOTU59958 | 1 | 0 | 0 | 0 | 1 | 0 | 0 |
| New.CleanUp.ReferenceOTU60469 | 0 | 1 | 0 | 1 | 0 | 1 | 0 |
| New.CleanUp.ReferenceOTU66093 | 0 | 1 | 0 | 1 | 0 | 0 | 0 |
| New.CleanUp.ReferenceOTU68700 | 1 | 0 | 1 | 1 | 1 | 0 | 0 |
| New.CleanUp.ReferenceOTU20824 | 0 | 0 | 0 | 0 | 0 | 1 | 0 |
| New.CleanUp.ReferenceOTU63135 | 0 | 0 | 0 | 1 | 0 | 1 | 1 |
| New.CleanUp.ReferenceOTU71784 | 0 | 1 | 0 | 1 | 0 | 0 | 1 |
| New.CleanUp.ReferenceOTU48338 | 0 | 1 | 1 | 0 | 1 | 0 | 0 |
| X70809.1.1793 | 1 | 1 | 1 | 0 | 1 | 0 | 0 |
| X70808.1.1867 | 0 | 1 | 1 | 0 | 1 | 0 | 0 |
| New.CleanUp.ReferenceOTU29427 | 1 | 1 | 1 | 0 | 1 | 0 | 0 |
| New.CleanUp.ReferenceOTU41035 | 0 | 0 | 1 | 0 | 1 | 0 | 0 |
| New.ReferenceOTU321 | 0 | 1 | 1 | 1 | 0 | 0 | 0 |
| New.CleanUp.ReferenceOTU979 | 1 | 1 | 0 | 1 | 0 | 1 | 0 |
| New.CleanUp.ReferenceOTU21302 | 0 | 1 | 0 | 1 | 0 | 0 | 0 |
| New.CleanUp.ReferenceOTU66179 | 0 | 1 | 0 | 1 | 0 | 0 | 0 |
| New.CleanUp.ReferenceOTU78385 | 0 | 1 | 1 | 0 | 0 | 0 | 0 |
| AF076172.1.1708 | 1 | 0 | 1 | 1 | 1 | 1 | 0 |
| New.CleanUp.ReferenceOTU21819 | 1 | 0 | 1 | 1 | 1 | 1 | 0 |
| New.CleanUp.ReferenceOTU17349 | 0 | 1 | 0 | 1 | 0 | 0 | 1 |
| New.CleanUp.ReferenceOTU48328 | 0 | 0 | 0 | 1 | 0 | 1 | 1 |
| New.CleanUp.ReferenceOTU7591 | 0 | 1 | 0 | 1 | 0 | 0 | 0 |
| New.CleanUp.ReferenceOTU10182 | 0 | 0 | 1 | 0 | 0 | 0 | 0 |
| New.CleanUp.ReferenceOTU21795 | 0 | 0 | 1 | 0 | 0 | 0 | 0 |
| New.CleanUp.ReferenceOTU68556 | 0 | 1 | 0 | 1 | 0 | 0 | 0 |
| New.CleanUp.ReferenceOTU48359 | 0 | 1 | 0 | 0 | 0 | 0 | 0 |
| AF166380.1.1793 | 1 | 1 | 1 | 1 | 1 | 1 | 1 |
| U05039.1.1805 | 1 | 1 | 1 | 1 | 1 | 1 | 0 |
| KU561107.1.1719 | 1 | 0 | 1 | 0 | 0 | 0 | 0 |
| New.ReferenceOTU149 | 1 | 1 | 1 | 1 | 1 | 1 | 0 |
| New.CleanUp.ReferenceOTU23592 | 1 | 0 | 0 | 0 | 0 | 0 | 0 |
| New.CleanUp.ReferenceOTU26017 | 1 | 0 | 0 | 0 | 1 | 0 | 0 |
| New.CleanUp.ReferenceOTU78860 | 1 | 0 | 0 | 0 | 0 | 0 | 0 |
| EF527133.1.1840 | 1 | 1 | 1 | 1 | 1 | 1 | 0 |
| FN562449.1.1771 | 0 | 0 | 0 | 1 | 1 | 1 | 0 |
| KF615766.1.1681 | 0 | 1 | 0 | 1 | 0 | 0 | 0 |
| New.ReferenceOTU180 | 1 | 1 | 1 | 1 | 1 | 1 | 1 |
| New.ReferenceOTU265 | 1 | 1 | 1 | 1 | 1 | 1 | 1 |
| New.ReferenceOTU219 | 1 | 1 | 1 | 1 | 1 | 1 | 1 |
| New.CleanUp.ReferenceOTU4778 | 0 | 0 | 1 | 1 | 1 | 1 | 1 |
| New.CleanUp.ReferenceOTU5151 | 0 | 0 | 0 | 1 | 1 | 1 | 0 |
| New.CleanUp.ReferenceOTU21156 | 0 | 1 | 0 | 1 | 0 | 0 | 0 |
| New.CleanUp.ReferenceOTU22262 | 0 | 1 | 1 | 1 | 1 | 1 | 1 |
| New.CleanUp.ReferenceOTU22973 | 0 | 0 | 0 | 0 | 1 | 0 | 0 |
| New.CleanUp.ReferenceOTU27904 | 0 | 0 | 0 | 0 | 0 | 1 | 1 |
| New.CleanUp.ReferenceOTU28601 | 0 | 0 | 0 | 0 | 1 | 0 | 0 |
| New.CleanUp.ReferenceOTU35840 | 0 | 1 | 1 | 1 | 1 | 1 | 1 |
| New.CleanUp.ReferenceOTU37348 | 0 | 0 | 0 | 1 | 1 | 1 | 0 |
| New.CleanUp.ReferenceOTU53235 | 0 | 1 | 0 | 1 | 1 | 1 | 0 |
| New.CleanUp.ReferenceOTU59503 | 0 | 0 | 1 | 1 | 1 | 0 | 0 |
| New.CleanUp.ReferenceOTU59623 | 0 | 1 | 1 | 0 | 0 | 0 | 0 |
| New.CleanUp.ReferenceOTU66492 | 0 | 0 | 0 | 0 | 1 | 0 | 0 |
| New.CleanUp.ReferenceOTU67518 | 0 | 0 | 0 | 0 | 0 | 1 | 0 |
| New.CleanUp.ReferenceOTU68292 | 1 | 0 | 1 | 0 | 1 | 0 | 0 |
| New.CleanUp.ReferenceOTU69142 | 0 | 1 | 0 | 1 | 1 | 1 | 0 |
| New.CleanUp.ReferenceOTU69717 | 0 | 1 | 1 | 0 | 0 | 0 | 0 |
| New.CleanUp.ReferenceOTU69965 | 1 | 0 | 1 | 1 | 1 | 0 | 1 |
| New.CleanUp.ReferenceOTU71842 | 0 | 1 | 1 | 0 | 1 | 1 | 0 |
| New.CleanUp.ReferenceOTU79494 | 1 | 1 | 1 | 1 | 1 | 1 | 1 |
| New.CleanUp.ReferenceOTU9521 | 0 | 0 | 1 | 1 | 0 | 1 | 1 |
| New.CleanUp.ReferenceOTU52142 | 0 | 1 | 0 | 1 | 1 | 1 | 1 |
| New.ReferenceOTU158 | 1 | 1 | 1 | 1 | 1 | 1 | 1 |
| New.CleanUp.ReferenceOTU8177 | 0 | 0 | 1 | 1 | 1 | 1 | 1 |
| New.CleanUp.ReferenceOTU12510 | 0 | 0 | 0 | 0 | 0 | 1 | 1 |
| New.CleanUp.ReferenceOTU16516 | 0 | 0 | 0 | 1 | 1 | 1 | 1 |
| New.CleanUp.ReferenceOTU18615 | 0 | 0 | 0 | 0 | 1 | 1 | 0 |
| New.CleanUp.ReferenceOTU22807 | 0 | 1 | 0 | 1 | 1 | 1 | 1 |
| New.CleanUp.ReferenceOTU24774 | 0 | 1 | 0 | 0 | 0 | 0 | 0 |
| New.CleanUp.ReferenceOTU26079 | 0 | 1 | 1 | 1 | 0 | 1 | 1 |
| New.CleanUp.ReferenceOTU27363 | 0 | 0 | 1 | 1 | 1 | 1 | 0 |
| New.CleanUp.ReferenceOTU28037 | 0 | 1 | 1 | 1 | 1 | 1 | 1 |
| New.CleanUp.ReferenceOTU28261 | 0 | 0 | 0 | 1 | 1 | 0 | 1 |
| New.CleanUp.ReferenceOTU30511 | 0 | 1 | 1 | 0 | 1 | 1 | 1 |
| New.CleanUp.ReferenceOTU32912 | 0 | 0 | 0 | 1 | 0 | 1 | 0 |
| New.CleanUp.ReferenceOTU33713 | 0 | 0 | 0 | 0 | 1 | 0 | 0 |
| New.CleanUp.ReferenceOTU40312 | 0 | 0 | 0 | 0 | 1 | 0 | 1 |
| New.CleanUp.ReferenceOTU41466 | 0 | 1 | 0 | 1 | 1 | 1 | 1 |
| New.CleanUp.ReferenceOTU41691 | 0 | 1 | 1 | 0 | 0 | 1 | 0 |
| New.CleanUp.ReferenceOTU42241 | 0 | 1 | 0 | 0 | 0 | 1 | 1 |
| New.CleanUp.ReferenceOTU45399 | 0 | 0 | 1 | 0 | 1 | 0 | 0 |
| New.CleanUp.ReferenceOTU50531 | 1 | 1 | 1 | 1 | 1 | 1 | 1 |
| New.CleanUp.ReferenceOTU50929 | 0 | 0 | 1 | 0 | 1 | 1 | 1 |
| New.CleanUp.ReferenceOTU51514 | 0 | 1 | 0 | 0 | 0 | 1 | 1 |
| New.CleanUp.ReferenceOTU54298 | 0 | 0 | 0 | 0 | 0 | 1 | 0 |
| New.CleanUp.ReferenceOTU54905 | 0 | 0 | 0 | 0 | 1 | 1 | 0 |
| New.CleanUp.ReferenceOTU55974 | 0 | 0 | 0 | 1 | 1 | 0 | 0 |
| New.CleanUp.ReferenceOTU56137 | 0 | 0 | 0 | 0 | 0 | 1 | 0 |
| New.CleanUp.ReferenceOTU56745 | 0 | 0 | 0 | 1 | 0 | 1 | 0 |
| New.CleanUp.ReferenceOTU57618 | 0 | 1 | 1 | 0 | 0 | 1 | 1 |
| New.CleanUp.ReferenceOTU59351 | 0 | 1 | 1 | 1 | 1 | 1 | 1 |
| New.CleanUp.ReferenceOTU59597 | 0 | 0 | 0 | 0 | 0 | 0 | 1 |
| New.CleanUp.ReferenceOTU60552 | 0 | 0 | 1 | 0 | 0 | 1 | 1 |
| New.CleanUp.ReferenceOTU61487 | 0 | 1 | 0 | 0 | 0 | 1 | 0 |
| New.CleanUp.ReferenceOTU61567 | 0 | 0 | 1 | 1 | 1 | 1 | 0 |
| New.CleanUp.ReferenceOTU62182 | 0 | 1 | 1 | 1 | 1 | 1 | 1 |
| New.CleanUp.ReferenceOTU62754 | 1 | 1 | 1 | 1 | 1 | 1 | 1 |
| New.CleanUp.ReferenceOTU63862 | 0 | 0 | 0 | 1 | 0 | 0 | 1 |
| New.CleanUp.ReferenceOTU64188 | 0 | 1 | 0 | 1 | 1 | 1 | 0 |
| New.CleanUp.ReferenceOTU67222 | 0 | 0 | 0 | 0 | 1 | 1 | 0 |
| New.CleanUp.ReferenceOTU69220 | 0 | 1 | 0 | 0 | 1 | 0 | 1 |
| New.CleanUp.ReferenceOTU70117 | 1 | 0 | 1 | 0 | 1 | 0 | 0 |
| New.CleanUp.ReferenceOTU70494 | 0 | 1 | 1 | 1 | 1 | 1 | 1 |
| New.CleanUp.ReferenceOTU70954 | 1 | 0 | 0 | 0 | 0 | 1 | 1 |
| New.CleanUp.ReferenceOTU72214 | 0 | 0 | 1 | 0 | 1 | 1 | 1 |
| New.CleanUp.ReferenceOTU74843 | 0 | 1 | 0 | 0 | 0 | 1 | 1 |
| New.CleanUp.ReferenceOTU74979 | 0 | 1 | 1 | 0 | 1 | 0 | 0 |
| AB183628.1.1747 | 1 | 1 | 1 | 1 | 1 | 1 | 1 |
| AB771819.1.1673 | 0 | 0 | 0 | 0 | 1 | 1 | 1 |
| New.CleanUp.ReferenceOTU974 | 0 | 1 | 0 | 1 | 1 | 0 | 0 |
| New.CleanUp.ReferenceOTU34048 | 0 | 0 | 1 | 1 | 0 | 0 | 1 |
| New.CleanUp.ReferenceOTU39689 | 0 | 0 | 0 | 1 | 0 | 1 | 0 |
| New.CleanUp.ReferenceOTU43943 | 0 | 0 | 0 | 1 | 1 | 0 | 0 |
| New.CleanUp.ReferenceOTU78278 | 1 | 0 | 0 | 0 | 0 | 1 | 1 |
| New.CleanUp.ReferenceOTU76486 | 1 | 1 | 1 | 0 | 0 | 1 | 1 |
| AY454430.1.1748 | 1 | 1 | 1 | 0 | 0 | 1 | 1 |
| AY454428.1.1750 | 0 | 0 | 0 | 1 | 0 | 0 | 0 |
| New.CleanUp.ReferenceOTU5657 | 0 | 0 | 0 | 1 | 0 | 0 | 0 |
| JX457431.1.1525 | 1 | 1 | 1 | 1 | 1 | 1 | 1 |
| AB971070.1.1246 | 0 | 1 | 1 | 1 | 1 | 0 | 1 |
| New.ReferenceOTU326 | 1 | 1 | 1 | 1 | 1 | 1 | 1 |
| New.CleanUp.ReferenceOTU2110 | 0 | 1 | 0 | 1 | 1 | 1 | 0 |
| New.CleanUp.ReferenceOTU2645 | 0 | 1 | 0 | 1 | 0 | 0 | 0 |
| New.CleanUp.ReferenceOTU12369 | 0 | 1 | 0 | 1 | 0 | 0 | 0 |
| New.CleanUp.ReferenceOTU16667 | 1 | 0 | 0 | 1 | 1 | 1 | 0 |
| New.CleanUp.ReferenceOTU19973 | 0 | 0 | 1 | 0 | 1 | 1 | 0 |
| New.CleanUp.ReferenceOTU21169 | 0 | 1 | 0 | 1 | 0 | 0 | 0 |
| New.CleanUp.ReferenceOTU22556 | 0 | 1 | 0 | 0 | 0 | 1 | 0 |
| New.CleanUp.ReferenceOTU24981 | 0 | 1 | 0 | 1 | 0 | 1 | 0 |
| New.CleanUp.ReferenceOTU25900 | 0 | 1 | 0 | 1 | 1 | 0 | 0 |
| New.CleanUp.ReferenceOTU27407 | 0 | 1 | 0 | 0 | 1 | 0 | 0 |
| New.CleanUp.ReferenceOTU28437 | 0 | 1 | 1 | 1 | 1 | 1 | 0 |
| New.CleanUp.ReferenceOTU29422 | 0 | 0 | 0 | 0 | 1 | 0 | 0 |
| New.CleanUp.ReferenceOTU30729 | 0 | 0 | 0 | 1 | 1 | 0 | 0 |
| New.CleanUp.ReferenceOTU33227 | 0 | 0 | 1 | 1 | 0 | 0 | 0 |
| New.CleanUp.ReferenceOTU35574 | 0 | 1 | 0 | 1 | 1 | 1 | 0 |
| New.CleanUp.ReferenceOTU41214 | 0 | 1 | 1 | 1 | 0 | 0 | 0 |
| New.CleanUp.ReferenceOTU43423 | 0 | 1 | 0 | 1 | 1 | 0 | 0 |
| New.CleanUp.ReferenceOTU43976 | 0 | 0 | 0 | 1 | 1 | 0 | 0 |
| New.CleanUp.ReferenceOTU48503 | 0 | 0 | 0 | 1 | 1 | 0 | 0 |
| New.CleanUp.ReferenceOTU48542 | 0 | 1 | 1 | 1 | 1 | 1 | 0 |
| New.CleanUp.ReferenceOTU51286 | 0 | 1 | 0 | 0 | 1 | 0 | 0 |
| New.CleanUp.ReferenceOTU52401 | 0 | 1 | 0 | 1 | 1 | 0 | 0 |
| New.CleanUp.ReferenceOTU58051 | 0 | 1 | 0 | 0 | 1 | 1 | 0 |
| New.CleanUp.ReferenceOTU62323 | 1 | 1 | 0 | 1 | 0 | 0 | 0 |
| New.CleanUp.ReferenceOTU62771 | 1 | 1 | 1 | 1 | 1 | 1 | 0 |
| New.CleanUp.ReferenceOTU66632 | 1 | 1 | 0 | 0 | 1 | 0 | 0 |
| New.CleanUp.ReferenceOTU67240 | 0 | 0 | 1 | 0 | 1 | 0 | 0 |
| New.CleanUp.ReferenceOTU72172 | 0 | 1 | 0 | 0 | 1 | 0 | 0 |
| New.CleanUp.ReferenceOTU75163 | 0 | 1 | 0 | 1 | 1 | 1 | 0 |
| New.CleanUp.ReferenceOTU77397 | 0 | 0 | 0 | 0 | 1 | 0 | 0 |
| New.CleanUp.ReferenceOTU77884 | 0 | 1 | 0 | 0 | 0 | 0 | 0 |
| New.CleanUp.ReferenceOTU33545 | 0 | 0 | 0 | 0 | 1 | 0 | 0 |
| New.CleanUp.ReferenceOTU38415 | 0 | 0 | 0 | 0 | 1 | 0 | 0 |
| JQ420120.1.1784 | 0 | 0 | 0 | 0 | 0 | 1 | 0 |
| New.CleanUp.ReferenceOTU35971 | 0 | 0 | 0 | 0 | 0 | 1 | 0 |
| Z21553.1.1796 | 0 | 1 | 0 | 0 | 0 | 0 | 0 |
| New.CleanUp.ReferenceOTU71095 | 0 | 1 | 0 | 0 | 0 | 0 | 0 |
| FM205882.1.2379 | 0 | 1 | 0 | 0 | 0 | 0 | 0 |
| New.CleanUp.ReferenceOTU64838 | 0 | 1 | 0 | 0 | 0 | 0 | 0 |
| JF791076.1.1777 | 0 | 1 | 1 | 1 | 1 | 1 | 1 |
| New.CleanUp.ReferenceOTU39482 | 0 | 1 | 0 | 1 | 0 | 0 | 0 |
| New.CleanUp.ReferenceOTU45930 | 0 | 1 | 1 | 0 | 0 | 0 | 0 |
| New.CleanUp.ReferenceOTU8557 | 0 | 0 | 0 | 0 | 0 | 1 | 0 |
| New.CleanUp.ReferenceOTU40061 | 0 | 0 | 0 | 0 | 0 | 1 | 0 |
| DQ310223.1.1390 | 1 | 1 | 0 | 1 | 0 | 1 | 0 |
| New.CleanUp.ReferenceOTU4003 | 1 | 1 | 1 | 1 | 0 | 1 | 0 |
| New.CleanUp.ReferenceOTU6410 | 1 | 0 | 0 | 0 | 0 | 0 | 0 |
| New.CleanUp.ReferenceOTU16283 | 0 | 1 | 0 | 1 | 1 | 0 | 0 |
| New.CleanUp.ReferenceOTU24771 | 0 | 1 | 0 | 0 | 0 | 1 | 0 |
| New.CleanUp.ReferenceOTU30596 | 0 | 1 | 0 | 1 | 0 | 1 | 0 |
| New.CleanUp.ReferenceOTU32345 | 0 | 1 | 0 | 0 | 0 | 0 | 0 |
| New.CleanUp.ReferenceOTU33068 | 1 | 1 | 1 | 1 | 1 | 0 | 0 |
| New.CleanUp.ReferenceOTU34579 | 1 | 0 | 1 | 0 | 0 | 0 | 0 |
| New.CleanUp.ReferenceOTU35258 | 1 | 0 | 0 | 0 | 0 | 0 | 0 |
| New.CleanUp.ReferenceOTU41469 | 0 | 1 | 1 | 1 | 0 | 0 | 0 |
| New.CleanUp.ReferenceOTU42442 | 0 | 1 | 0 | 1 | 0 | 1 | 0 |
| New.CleanUp.ReferenceOTU44187 | 0 | 0 | 1 | 0 | 0 | 0 | 0 |
| New.CleanUp.ReferenceOTU50193 | 0 | 1 | 1 | 1 | 0 | 0 | 0 |
| New.CleanUp.ReferenceOTU53879 | 0 | 1 | 1 | 0 | 0 | 0 | 0 |
| New.CleanUp.ReferenceOTU61927 | 0 | 1 | 0 | 1 | 0 | 1 | 0 |
| New.CleanUp.ReferenceOTU62281 | 1 | 1 | 0 | 0 | 0 | 1 | 0 |
| New.CleanUp.ReferenceOTU64944 | 0 | 1 | 1 | 1 | 0 | 0 | 0 |
| New.CleanUp.ReferenceOTU65136 | 1 | 1 | 1 | 1 | 0 | 1 | 1 |
| New.CleanUp.ReferenceOTU65144 | 0 | 1 | 1 | 0 | 0 | 0 | 0 |
| New.CleanUp.ReferenceOTU67425 | 1 | 1 | 1 | 0 | 0 | 1 | 0 |
| New.CleanUp.ReferenceOTU69373 | 1 | 0 | 0 | 0 | 0 | 0 | 0 |
| New.CleanUp.ReferenceOTU70342 | 1 | 1 | 1 | 1 | 1 | 1 | 1 |
| New.CleanUp.ReferenceOTU73544 | 0 | 1 | 1 | 1 | 0 | 0 | 1 |
| New.CleanUp.ReferenceOTU80876 | 0 | 1 | 0 | 0 | 1 | 1 | 1 |
| New.CleanUp.ReferenceOTU81144 | 0 | 1 | 1 | 1 | 0 | 0 | 0 |
| JF791073.1.1777 | 0 | 1 | 1 | 1 | 1 | 1 | 0 |
| New.CleanUp.ReferenceOTU34330 | 1 | 1 | 1 | 1 | 1 | 1 | 1 |
| New.CleanUp.ReferenceOTU43133 | 1 | 1 | 1 | 1 | 1 | 1 | 1 |
| New.CleanUp.ReferenceOTU65083 | 0 | 1 | 1 | 1 | 1 | 1 | 0 |
| New.CleanUp.ReferenceOTU2909 | 0 | 0 | 1 | 1 | 1 | 1 | 0 |
| New.CleanUp.ReferenceOTU10075 | 1 | 0 | 0 | 0 | 0 | 0 | 0 |
| New.CleanUp.ReferenceOTU20453 | 0 | 1 | 1 | 0 | 0 | 1 | 0 |
| New.CleanUp.ReferenceOTU21944 | 1 | 1 | 0 | 1 | 0 | 0 | 1 |
| New.CleanUp.ReferenceOTU23316 | 1 | 0 | 0 | 0 | 1 | 0 | 0 |
| New.CleanUp.ReferenceOTU32108 | 0 | 1 | 1 | 0 | 0 | 0 | 0 |
| New.CleanUp.ReferenceOTU33164 | 0 | 1 | 0 | 1 | 0 | 0 | 0 |
| New.CleanUp.ReferenceOTU45807 | 0 | 0 | 0 | 0 | 0 | 1 | 0 |
| New.CleanUp.ReferenceOTU47761 | 0 | 0 | 1 | 0 | 0 | 0 | 0 |
| New.CleanUp.ReferenceOTU51398 | 1 | 1 | 1 | 0 | 1 | 0 | 0 |
| New.CleanUp.ReferenceOTU54604 | 1 | 0 | 0 | 0 | 0 | 0 | 0 |
| New.CleanUp.ReferenceOTU58724 | 0 | 0 | 1 | 1 | 0 | 1 | 0 |
| New.CleanUp.ReferenceOTU67062 | 0 | 1 | 0 | 0 | 0 | 0 | 0 |
| New.CleanUp.ReferenceOTU75004 | 0 | 1 | 0 | 0 | 0 | 0 | 0 |
| KU743815.1.1774 | 1 | 1 | 1 | 1 | 1 | 0 | 1 |
| New.ReferenceOTU31 | 1 | 1 | 1 | 1 | 1 | 0 | 1 |
| New.CleanUp.ReferenceOTU649 | 0 | 0 | 1 | 1 | 0 | 0 | 1 |
| New.CleanUp.ReferenceOTU7713 | 0 | 0 | 1 | 1 | 0 | 0 | 0 |
| New.CleanUp.ReferenceOTU9383 | 0 | 1 | 1 | 0 | 0 | 0 | 0 |
| New.CleanUp.ReferenceOTU13744 | 0 | 0 | 0 | 1 | 0 | 0 | 0 |
| New.CleanUp.ReferenceOTU20329 | 0 | 1 | 0 | 1 | 1 | 0 | 0 |
| New.CleanUp.ReferenceOTU21649 | 0 | 1 | 0 | 1 | 0 | 0 | 0 |
| New.CleanUp.ReferenceOTU23408 | 0 | 0 | 1 | 1 | 0 | 0 | 1 |
| New.CleanUp.ReferenceOTU29281 | 0 | 0 | 0 | 1 | 0 | 0 | 1 |
| New.CleanUp.ReferenceOTU29928 | 0 | 0 | 1 | 1 | 0 | 0 | 0 |
| New.CleanUp.ReferenceOTU35313 | 0 | 0 | 1 | 0 | 0 | 0 | 0 |
| New.CleanUp.ReferenceOTU46853 | 0 | 1 | 1 | 1 | 0 | 0 | 0 |
| New.CleanUp.ReferenceOTU53948 | 0 | 1 | 1 | 1 | 0 | 0 | 0 |
| New.CleanUp.ReferenceOTU56633 | 0 | 1 | 0 | 1 | 0 | 0 | 0 |
| New.CleanUp.ReferenceOTU59562 | 0 | 1 | 1 | 1 | 0 | 0 | 0 |
| New.CleanUp.ReferenceOTU62861 | 0 | 1 | 1 | 0 | 0 | 0 | 0 |
| New.CleanUp.ReferenceOTU65310 | 0 | 0 | 1 | 1 | 0 | 0 | 0 |
| New.CleanUp.ReferenceOTU66623 | 1 | 1 | 1 | 0 | 0 | 0 | 1 |
| New.CleanUp.ReferenceOTU66891 | 0 | 1 | 0 | 1 | 0 | 0 | 1 |
| New.CleanUp.ReferenceOTU67254 | 0 | 0 | 0 | 1 | 0 | 0 | 0 |
| New.CleanUp.ReferenceOTU67698 | 1 | 1 | 1 | 1 | 1 | 0 | 1 |
| New.CleanUp.ReferenceOTU71068 | 0 | 0 | 1 | 0 | 0 | 0 | 0 |
| New.CleanUp.ReferenceOTU71860 | 0 | 1 | 1 | 0 | 0 | 0 | 0 |
| New.CleanUp.ReferenceOTU78120 | 0 | 1 | 0 | 1 | 0 | 0 | 0 |
| New.CleanUp.ReferenceOTU80838 | 0 | 0 | 0 | 1 | 0 | 0 | 0 |
| New.CleanUp.ReferenceOTU80904 | 0 | 1 | 1 | 1 | 0 | 0 | 0 |
| New.CleanUp.ReferenceOTU14296 | 0 | 1 | 1 | 1 | 0 | 0 | 0 |
| New.CleanUp.ReferenceOTU24733 | 0 | 1 | 1 | 1 | 0 | 0 | 0 |
| New.CleanUp.ReferenceOTU39500 | 0 | 1 | 1 | 1 | 0 | 0 | 0 |
| New.CleanUp.ReferenceOTU40869 | 0 | 1 | 1 | 1 | 0 | 0 | 0 |
| New.CleanUp.ReferenceOTU1301 | 0 | 0 | 0 | 0 | 0 | 1 | 0 |
| New.CleanUp.ReferenceOTU59745 | 0 | 0 | 0 | 0 | 0 | 1 | 0 |
| AJ007278.1.1744 | 0 | 0 | 1 | 0 | 0 | 1 | 0 |
| New.CleanUp.ReferenceOTU64135 | 0 | 0 | 1 | 0 | 0 | 1 | 0 |
| New.CleanUp.ReferenceOTU64713 | 0 | 1 | 0 | 0 | 1 | 0 | 0 |
| New.CleanUp.ReferenceOTU14051 | 0 | 0 | 0 | 0 | 0 | 1 | 0 |
| New.CleanUp.ReferenceOTU46057 | 0 | 0 | 0 | 0 | 0 | 1 | 0 |
| New.CleanUp.ReferenceOTU74926 | 0 | 0 | 0 | 0 | 0 | 1 | 0 |
| AJ420694.1.1835 | 1 | 1 | 0 | 0 | 0 | 0 | 0 |
| New.ReferenceOTU177 | 1 | 1 | 0 | 0 | 0 | 0 | 0 |
| New.CleanUp.ReferenceOTU5257 | 1 | 1 | 1 | 1 | 0 | 1 | 0 |
| New.CleanUp.ReferenceOTU19443 | 0 | 0 | 0 | 0 | 0 | 1 | 0 |
| New.CleanUp.ReferenceOTU27042 | 0 | 0 | 0 | 0 | 0 | 1 | 0 |
| New.CleanUp.ReferenceOTU36218 | 1 | 1 | 1 | 1 | 0 | 1 | 0 |
| New.CleanUp.ReferenceOTU43994 | 1 | 0 | 0 | 0 | 0 | 0 | 0 |
| New.CleanUp.ReferenceOTU51372 | 0 | 1 | 1 | 1 | 0 | 0 | 0 |
| New.CleanUp.ReferenceOTU75130 | 1 | 0 | 0 | 0 | 0 | 0 | 0 |
| X55032.1.1881 | 0 | 0 | 0 | 0 | 0 | 1 | 0 |
| New.CleanUp.ReferenceOTU8480 | 1 | 1 | 1 | 0 | 0 | 0 | 0 |
| New.CleanUp.ReferenceOTU16052 | 1 | 1 | 1 | 0 | 0 | 0 | 0 |
| New.CleanUp.ReferenceOTU41568 | 0 | 0 | 0 | 0 | 0 | 1 | 0 |
| New.CleanUp.ReferenceOTU54224 | 1 | 0 | 1 | 0 | 0 | 0 | 0 |
| New.CleanUp.ReferenceOTU55145 | 0 | 1 | 0 | 0 | 0 | 1 | 0 |
| New.CleanUp.ReferenceOTU66997 | 0 | 1 | 1 | 1 | 0 | 1 | 1 |
| New.CleanUp.ReferenceOTU75559 | 0 | 1 | 1 | 1 | 0 | 1 | 1 |
| New.CleanUp.ReferenceOTU78631 | 0 | 1 | 0 | 0 | 0 | 1 | 0 |
| New.CleanUp.ReferenceOTU8288 | 0 | 1 | 0 | 0 | 0 | 0 | 1 |
| New.CleanUp.ReferenceOTU18698 | 0 | 1 | 0 | 0 | 0 | 0 | 1 |
| New.CleanUp.ReferenceOTU23011 | 1 | 1 | 0 | 1 | 0 | 0 | 0 |
| New.CleanUp.ReferenceOTU36437 | 1 | 0 | 1 | 0 | 0 | 0 | 0 |
| New.CleanUp.ReferenceOTU45779 | 1 | 1 | 0 | 1 | 0 | 0 | 0 |
| New.CleanUp.ReferenceOTU58155 | 0 | 0 | 1 | 0 | 0 | 0 | 0 |
| New.CleanUp.ReferenceOTU67733 | 0 | 0 | 0 | 0 | 0 | 1 | 0 |
| New.CleanUp.ReferenceOTU77956 | 0 | 0 | 1 | 0 | 0 | 0 | 0 |
| New.CleanUp.ReferenceOTU18104 | 1 | 1 | 0 | 1 | 0 | 0 | 0 |
| New.ReferenceOTU209 | 1 | 1 | 1 | 1 | 1 | 1 | 1 |
| New.ReferenceOTU252 | 0 | 1 | 1 | 1 | 1 | 1 | 1 |
| New.ReferenceOTU14 | 0 | 1 | 1 | 1 | 1 | 1 | 1 |
| New.CleanUp.ReferenceOTU19408 | 0 | 0 | 0 | 0 | 0 | 1 | 0 |
| New.CleanUp.ReferenceOTU7321 | 0 | 0 | 0 | 0 | 1 | 1 | 0 |
| New.CleanUp.ReferenceOTU18291 | 0 | 0 | 0 | 0 | 0 | 1 | 0 |
| New.CleanUp.ReferenceOTU20723 | 0 | 1 | 1 | 0 | 1 | 0 | 1 |
| New.CleanUp.ReferenceOTU45081 | 0 | 1 | 0 | 0 | 0 | 1 | 1 |
| New.CleanUp.ReferenceOTU54441 | 0 | 0 | 0 | 0 | 1 | 1 | 0 |
| New.CleanUp.ReferenceOTU59226 | 0 | 0 | 0 | 0 | 1 | 1 | 0 |
| New.CleanUp.ReferenceOTU71732 | 0 | 1 | 0 | 0 | 0 | 1 | 0 |
| AB240962.1.1610 | 0 | 1 | 1 | 1 | 1 | 1 | 1 |
| FN690456.1.1738 | 0 | 0 | 1 | 1 | 1 | 1 | 0 |
| KJ763828.1.1774 | 0 | 1 | 1 | 0 | 0 | 1 | 0 |
| JF790984.1.1777 | 0 | 1 | 1 | 1 | 1 | 1 | 1 |
| New.ReferenceOTU197 | 1 | 1 | 1 | 1 | 1 | 1 | 1 |
| New.ReferenceOTU47 | 1 | 1 | 1 | 1 | 1 | 1 | 0 |
| New.CleanUp.ReferenceOTU11077 | 0 | 1 | 1 | 1 | 1 | 1 | 1 |
| New.CleanUp.ReferenceOTU20047 | 1 | 1 | 1 | 1 | 1 | 1 | 0 |
| New.CleanUp.ReferenceOTU39733 | 0 | 0 | 0 | 0 | 1 | 1 | 0 |
| New.CleanUp.ReferenceOTU42061 | 0 | 1 | 1 | 1 | 1 | 1 | 1 |
| New.CleanUp.ReferenceOTU72527 | 0 | 0 | 0 | 0 | 0 | 1 | 0 |
| JQ420121.1.1760 | 0 | 0 | 1 | 0 | 1 | 0 | 0 |
| U53132.1.1770 | 1 | 1 | 1 | 1 | 1 | 1 | 1 |
| New.ReferenceOTU204 | 1 | 1 | 1 | 1 | 1 | 1 | 1 |
| New.ReferenceOTU95 | 1 | 1 | 1 | 0 | 1 | 1 | 1 |
| New.ReferenceOTU64 | 0 | 1 | 1 | 1 | 1 | 1 | 1 |
| New.ReferenceOTU122 | 1 | 1 | 1 | 1 | 1 | 1 | 1 |
| New.CleanUp.ReferenceOTU1270 | 0 | 1 | 0 | 1 | 0 | 0 | 1 |
| New.CleanUp.ReferenceOTU2811 | 0 | 0 | 0 | 0 | 0 | 1 | 0 |
| New.CleanUp.ReferenceOTU4089 | 0 | 1 | 1 | 0 | 0 | 0 | 0 |
| New.CleanUp.ReferenceOTU6163 | 0 | 0 | 0 | 0 | 1 | 1 | 0 |
| New.CleanUp.ReferenceOTU6499 | 0 | 0 | 1 | 0 | 0 | 1 | 0 |
| New.CleanUp.ReferenceOTU7035 | 1 | 1 | 0 | 0 | 0 | 0 | 0 |
| New.CleanUp.ReferenceOTU7823 | 1 | 0 | 0 | 0 | 0 | 0 | 0 |
| New.CleanUp.ReferenceOTU8117 | 0 | 1 | 0 | 0 | 1 | 0 | 0 |
| New.CleanUp.ReferenceOTU9185 | 0 | 1 | 1 | 1 | 0 | 0 | 1 |
| New.CleanUp.ReferenceOTU12152 | 1 | 0 | 0 | 0 | 0 | 0 | 0 |
| New.CleanUp.ReferenceOTU13387 | 1 | 0 | 0 | 0 | 0 | 0 | 0 |
| New.CleanUp.ReferenceOTU14327 | 0 | 0 | 0 | 0 | 1 | 1 | 0 |
| New.CleanUp.ReferenceOTU15357 | 1 | 1 | 1 | 1 | 0 | 1 | 1 |
| New.CleanUp.ReferenceOTU16742 | 0 | 0 | 0 | 0 | 1 | 1 | 0 |
| New.CleanUp.ReferenceOTU17284 | 1 | 1 | 0 | 0 | 0 | 0 | 0 |
| New.CleanUp.ReferenceOTU18037 | 1 | 0 | 0 | 0 | 0 | 0 | 0 |
| New.CleanUp.ReferenceOTU23555 | 0 | 1 | 1 | 0 | 1 | 1 | 0 |
| New.CleanUp.ReferenceOTU23712 | 0 | 0 | 0 | 0 | 0 | 1 | 0 |
| New.CleanUp.ReferenceOTU24849 | 1 | 0 | 0 | 0 | 0 | 0 | 0 |
| New.CleanUp.ReferenceOTU24890 | 0 | 1 | 0 | 1 | 1 | 1 | 0 |
| New.CleanUp.ReferenceOTU28524 | 1 | 1 | 1 | 0 | 0 | 0 | 0 |
| New.CleanUp.ReferenceOTU28736 | 0 | 0 | 0 | 0 | 0 | 1 | 0 |
| New.CleanUp.ReferenceOTU29207 | 0 | 1 | 0 | 1 | 0 | 0 | 0 |
| New.CleanUp.ReferenceOTU30948 | 1 | 0 | 0 | 1 | 0 | 1 | 0 |
| New.CleanUp.ReferenceOTU31550 | 0 | 0 | 0 | 0 | 0 | 1 | 1 |
| New.CleanUp.ReferenceOTU32948 | 0 | 1 | 0 | 0 | 1 | 0 | 1 |
| New.CleanUp.ReferenceOTU33780 | 1 | 1 | 0 | 0 | 0 | 0 | 0 |
| New.CleanUp.ReferenceOTU34446 | 0 | 0 | 1 | 0 | 0 | 0 | 0 |
| New.CleanUp.ReferenceOTU34892 | 1 | 1 | 0 | 1 | 1 | 0 | 0 |
| New.CleanUp.ReferenceOTU34918 | 0 | 1 | 0 | 0 | 0 | 1 | 0 |
| New.CleanUp.ReferenceOTU36977 | 1 | 1 | 1 | 0 | 1 | 0 | 0 |
| New.CleanUp.ReferenceOTU37680 | 0 | 0 | 0 | 0 | 1 | 1 | 0 |
| New.CleanUp.ReferenceOTU38066 | 1 | 0 | 0 | 0 | 1 | 1 | 0 |
| New.CleanUp.ReferenceOTU39528 | 0 | 0 | 0 | 0 | 1 | 1 | 0 |
| New.CleanUp.ReferenceOTU39837 | 0 | 1 | 1 | 1 | 1 | 1 | 1 |
| New.CleanUp.ReferenceOTU39916 | 0 | 1 | 1 | 0 | 1 | 1 | 0 |
| New.CleanUp.ReferenceOTU41994 | 0 | 0 | 1 | 0 | 0 | 1 | 0 |
| New.CleanUp.ReferenceOTU42579 | 0 | 1 | 1 | 1 | 0 | 1 | 0 |
| New.CleanUp.ReferenceOTU44334 | 1 | 1 | 0 | 0 | 0 | 0 | 0 |
| New.CleanUp.ReferenceOTU47576 | 1 | 0 | 0 | 0 | 0 | 0 | 0 |
| New.CleanUp.ReferenceOTU51683 | 0 | 1 | 0 | 0 | 0 | 1 | 0 |
| New.CleanUp.ReferenceOTU52837 | 0 | 1 | 0 | 0 | 0 | 0 | 0 |
| New.CleanUp.ReferenceOTU54766 | 0 | 0 | 0 | 0 | 0 | 1 | 0 |
| New.CleanUp.ReferenceOTU57254 | 0 | 0 | 1 | 0 | 1 | 0 | 0 |
| New.CleanUp.ReferenceOTU57766 | 0 | 1 | 0 | 0 | 0 | 0 | 0 |
| New.CleanUp.ReferenceOTU63629 | 0 | 0 | 0 | 1 | 0 | 1 | 0 |
| New.CleanUp.ReferenceOTU64933 | 0 | 0 | 0 | 0 | 0 | 1 | 1 |
| New.CleanUp.ReferenceOTU66866 | 0 | 0 | 0 | 0 | 0 | 1 | 0 |
| New.CleanUp.ReferenceOTU67215 | 1 | 1 | 0 | 0 | 0 | 0 | 0 |
| New.CleanUp.ReferenceOTU67744 | 0 | 0 | 1 | 0 | 0 | 1 | 0 |
| New.CleanUp.ReferenceOTU68457 | 0 | 1 | 1 | 1 | 0 | 0 | 0 |
| New.CleanUp.ReferenceOTU70452 | 0 | 0 | 0 | 0 | 0 | 1 | 0 |
| New.CleanUp.ReferenceOTU71779 | 0 | 0 | 0 | 0 | 0 | 1 | 0 |
| New.CleanUp.ReferenceOTU72803 | 1 | 0 | 0 | 0 | 0 | 0 | 0 |
| New.CleanUp.ReferenceOTU73874 | 0 | 0 | 0 | 0 | 1 | 1 | 0 |
| New.CleanUp.ReferenceOTU75907 | 1 | 0 | 0 | 0 | 0 | 0 | 0 |
| New.CleanUp.ReferenceOTU76577 | 0 | 1 | 1 | 0 | 0 | 0 | 0 |
| New.CleanUp.ReferenceOTU77892 | 0 | 1 | 0 | 0 | 0 | 1 | 1 |
| New.CleanUp.ReferenceOTU78160 | 0 | 1 | 0 | 1 | 0 | 0 | 0 |
| New.CleanUp.ReferenceOTU79253 | 1 | 0 | 0 | 0 | 0 | 0 | 0 |
| New.CleanUp.ReferenceOTU81101 | 0 | 1 | 0 | 0 | 0 | 0 | 0 |
| New.CleanUp.ReferenceOTU81150 | 0 | 1 | 1 | 1 | 0 | 0 | 0 |
| JF714232.1.1791 | 0 | 1 | 1 | 1 | 0 | 1 | 0 |
| New.ReferenceOTU309 | 0 | 1 | 1 | 1 | 0 | 1 | 0 |
| New.CleanUp.ReferenceOTU2129 | 0 | 0 | 1 | 0 | 0 | 0 | 0 |
| New.CleanUp.ReferenceOTU72997 | 0 | 1 | 1 | 0 | 0 | 1 | 0 |
| New.ReferenceOTU295 | 1 | 1 | 0 | 0 | 0 | 0 | 0 |
| New.CleanUp.ReferenceOTU70429 | 1 | 1 | 0 | 0 | 0 | 0 | 0 |
| AM491022.1.1794 | 0 | 1 | 1 | 1 | 1 | 1 | 1 |
| New.ReferenceOTU210 | 0 | 1 | 1 | 1 | 1 | 1 | 1 |
| New.CleanUp.ReferenceOTU12844 | 0 | 1 | 1 | 0 | 1 | 0 | 0 |
| New.CleanUp.ReferenceOTU20789 | 0 | 0 | 1 | 0 | 0 | 0 | 0 |
| New.CleanUp.ReferenceOTU72944 | 0 | 0 | 1 | 0 | 0 | 0 | 0 |
| New.ReferenceOTU159 | 0 | 1 | 1 | 1 | 1 | 1 | 1 |
| KF130229.1.1762 | 0 | 1 | 1 | 0 | 0 | 1 | 0 |
| AJ246273.1.1799 | 0 | 1 | 1 | 0 | 0 | 0 | 1 |
| HM581603.1.1799 | 0 | 1 | 1 | 1 | 1 | 1 | 1 |
| FR874448.1.1798 | 0 | 1 | 1 | 1 | 1 | 1 | 1 |
| KF620971.1.1798 | 0 | 1 | 1 | 0 | 1 | 1 | 1 |
| KJ760407.1.1804 | 0 | 0 | 0 | 1 | 0 | 0 | 1 |
| New.CleanUp.ReferenceOTU6466 | 0 | 1 | 1 | 1 | 1 | 1 | 1 |
| New.CleanUp.ReferenceOTU13105 | 0 | 1 | 1 | 1 | 0 | 1 | 0 |
| New.CleanUp.ReferenceOTU71210 | 0 | 1 | 0 | 1 | 0 | 0 | 0 |
| New.CleanUp.ReferenceOTU61446 | 0 | 1 | 0 | 0 | 0 | 0 | 1 |
| New.CleanUp.ReferenceOTU21023 | 0 | 1 | 1 | 1 | 1 | 1 | 1 |
| New.CleanUp.ReferenceOTU29030 | 0 | 1 | 1 | 1 | 1 | 1 | 1 |
| New.CleanUp.ReferenceOTU3242 | 0 | 1 | 0 | 0 | 0 | 0 | 0 |
| New.CleanUp.ReferenceOTU80686 | 0 | 1 | 0 | 1 | 0 | 0 | 0 |
| New.CleanUp.ReferenceOTU18618 | 1 | 1 | 0 | 0 | 0 | 0 | 0 |
| X90992.1.1796 | 0 | 1 | 0 | 0 | 0 | 0 | 0 |
| New.CleanUp.ReferenceOTU57274 | 0 | 1 | 0 | 0 | 0 | 0 | 0 |
| KF620990.1.1809 | 0 | 1 | 0 | 1 | 1 | 1 | 0 |
| New.CleanUp.ReferenceOTU60323 | 0 | 1 | 0 | 1 | 1 | 1 | 0 |
| AM779755.1.1802 | 1 | 1 | 1 | 1 | 1 | 1 | 1 |
| KJ762999.1.1800 | 0 | 1 | 1 | 1 | 1 | 1 | 1 |
| GBYI01024087.1577.3305 | 1 | 1 | 0 | 1 | 0 | 0 | 0 |
| New.ReferenceOTU108 | 1 | 1 | 0 | 1 | 1 | 0 | 0 |
| New.ReferenceOTU205 | 1 | 1 | 1 | 1 | 0 | 1 | 1 |
| New.CleanUp.ReferenceOTU15979 | 1 | 1 | 0 | 1 | 0 | 0 | 0 |
| New.CleanUp.ReferenceOTU47289 | 1 | 1 | 0 | 0 | 0 | 0 | 0 |
| New.CleanUp.ReferenceOTU48027 | 1 | 1 | 0 | 0 | 0 | 0 | 1 |
| New.CleanUp.ReferenceOTU57002 | 1 | 0 | 0 | 0 | 1 | 0 | 0 |
| New.CleanUp.ReferenceOTU57718 | 1 | 1 | 0 | 1 | 0 | 0 | 0 |
| New.CleanUp.ReferenceOTU72457 | 1 | 1 | 0 | 0 | 0 | 0 | 0 |
| KP404851.1.1702 | 0 | 0 | 0 | 0 | 1 | 1 | 0 |
| New.CleanUp.ReferenceOTU7273 | 0 | 1 | 0 | 1 | 1 | 1 | 0 |
| New.CleanUp.ReferenceOTU59580 | 0 | 1 | 0 | 1 | 0 | 0 | 0 |
| New.CleanUp.ReferenceOTU59746 | 0 | 0 | 1 | 0 | 0 | 0 | 1 |
| New.CleanUp.ReferenceOTU28085 | 0 | 1 | 1 | 0 | 0 | 0 | 0 |
| New.CleanUp.ReferenceOTU81335 | 0 | 1 | 1 | 0 | 0 | 0 | 0 |
| AY208893.1.1819 | 0 | 1 | 0 | 1 | 1 | 1 | 0 |
| HQ658161.1.1742 | 0 | 1 | 1 | 1 | 1 | 1 | 1 |
| AF239260.1.1699 | 0 | 0 | 0 | 1 | 0 | 0 | 1 |
| GU647168.1.1540 | 0 | 1 | 1 | 1 | 1 | 1 | 1 |
| KC582924.1.1796 | 0 | 1 | 1 | 1 | 1 | 1 | 0 |
| DQ386753.1.1536 | 0 | 1 | 1 | 1 | 1 | 1 | 1 |
| KJ758904.1.1796 | 0 | 1 | 1 | 1 | 1 | 1 | 1 |
| AF472555.1.1812 | 1 | 1 | 1 | 1 | 1 | 1 | 1 |
| AY208894.1.1813 | 0 | 0 | 1 | 0 | 0 | 0 | 0 |
| AY775285.1.1789 | 0 | 1 | 0 | 1 | 1 | 0 | 1 |
| KJ763489.1.1801 | 0 | 1 | 1 | 1 | 1 | 1 | 1 |
| AY129040.1.1796 | 0 | 1 | 1 | 1 | 1 | 1 | 1 |
| KJ759356.1.1798 | 0 | 0 | 0 | 1 | 0 | 0 | 0 |
| KJ762980.1.1794 | 0 | 0 | 1 | 1 | 1 | 1 | 1 |
| KJ757626.1.1797 | 0 | 1 | 0 | 1 | 0 | 0 | 0 |
| New.ReferenceOTU132 | 0 | 1 | 0 | 1 | 1 | 0 | 1 |
| New.ReferenceOTU130 | 1 | 1 | 1 | 1 | 1 | 1 | 1 |
| New.ReferenceOTU178 | 1 | 1 | 1 | 1 | 1 | 1 | 1 |
| New.ReferenceOTU171 | 0 | 1 | 1 | 1 | 1 | 1 | 0 |
| New.ReferenceOTU101 | 0 | 1 | 1 | 1 | 1 | 1 | 1 |
| New.ReferenceOTU201 | 1 | 1 | 1 | 1 | 1 | 1 | 1 |
| New.ReferenceOTU148 | 0 | 1 | 1 | 1 | 1 | 1 | 1 |
| New.ReferenceOTU35 | 0 | 1 | 0 | 1 | 0 | 1 | 1 |
| New.ReferenceOTU127 | 1 | 1 | 1 | 1 | 1 | 1 | 1 |
| New.ReferenceOTU318 | 1 | 1 | 1 | 1 | 1 | 1 | 1 |
| New.ReferenceOTU169 | 0 | 1 | 1 | 1 | 1 | 1 | 1 |
| New.ReferenceOTU164 | 0 | 1 | 1 | 1 | 1 | 1 | 1 |
| New.ReferenceOTU189 | 0 | 1 | 1 | 1 | 1 | 1 | 1 |
| New.ReferenceOTU188 | 0 | 1 | 1 | 1 | 1 | 1 | 1 |
| New.ReferenceOTU211 | 0 | 1 | 0 | 1 | 1 | 1 | 0 |
| New.CleanUp.ReferenceOTU4 | 0 | 1 | 0 | 0 | 0 | 0 | 0 |
| New.CleanUp.ReferenceOTU100 | 0 | 1 | 0 | 1 | 1 | 0 | 0 |
| New.CleanUp.ReferenceOTU259 | 0 | 1 | 0 | 0 | 0 | 0 | 0 |
| New.CleanUp.ReferenceOTU311 | 0 | 1 | 0 | 0 | 1 | 0 | 1 |
| New.CleanUp.ReferenceOTU999 | 0 | 1 | 1 | 1 | 1 | 1 | 1 |
| New.CleanUp.ReferenceOTU1082 | 0 | 0 | 0 | 0 | 0 | 0 | 1 |
| New.CleanUp.ReferenceOTU1167 | 0 | 1 | 0 | 1 | 0 | 0 | 0 |
| New.CleanUp.ReferenceOTU1288 | 0 | 1 | 0 | 0 | 0 | 1 | 1 |
| New.CleanUp.ReferenceOTU1712 | 0 | 1 | 0 | 1 | 0 | 0 | 0 |
| New.CleanUp.ReferenceOTU1907 | 0 | 0 | 1 | 0 | 1 | 0 | 1 |
| New.CleanUp.ReferenceOTU1982 | 0 | 1 | 0 | 1 | 1 | 1 | 0 |
| New.CleanUp.ReferenceOTU2185 | 0 | 1 | 0 | 0 | 0 | 1 | 1 |
| New.CleanUp.ReferenceOTU2467 | 0 | 0 | 0 | 1 | 0 | 1 | 1 |
| New.CleanUp.ReferenceOTU2496 | 0 | 1 | 0 | 0 | 0 | 1 | 1 |
| New.CleanUp.ReferenceOTU2672 | 0 | 0 | 0 | 1 | 0 | 0 | 1 |
| New.CleanUp.ReferenceOTU3011 | 0 | 1 | 0 | 1 | 0 | 0 | 0 |
| New.CleanUp.ReferenceOTU3296 | 0 | 0 | 1 | 1 | 1 | 1 | 1 |
| New.CleanUp.ReferenceOTU3755 | 0 | 0 | 0 | 0 | 0 | 1 | 1 |
| New.CleanUp.ReferenceOTU4138 | 0 | 1 | 1 | 1 | 0 | 1 | 0 |
| New.CleanUp.ReferenceOTU4559 | 0 | 1 | 0 | 1 | 0 | 1 | 1 |
| New.CleanUp.ReferenceOTU4840 | 0 | 0 | 0 | 0 | 1 | 0 | 0 |
| New.CleanUp.ReferenceOTU4885 | 1 | 1 | 1 | 1 | 1 | 1 | 1 |
| New.CleanUp.ReferenceOTU5400 | 0 | 0 | 1 | 1 | 1 | 0 | 1 |
| New.CleanUp.ReferenceOTU5737 | 0 | 0 | 1 | 0 | 0 | 0 | 1 |
| New.CleanUp.ReferenceOTU5743 | 0 | 0 | 0 | 0 | 1 | 0 | 1 |
| New.CleanUp.ReferenceOTU6202 | 0 | 0 | 0 | 1 | 0 | 0 | 0 |
| New.CleanUp.ReferenceOTU6831 | 0 | 1 | 0 | 1 | 0 | 1 | 0 |
| New.CleanUp.ReferenceOTU7339 | 0 | 1 | 0 | 0 | 0 | 1 | 1 |
| New.CleanUp.ReferenceOTU7485 | 0 | 0 | 0 | 1 | 1 | 1 | 1 |
| New.CleanUp.ReferenceOTU9215 | 0 | 1 | 0 | 1 | 1 | 0 | 1 |
| New.CleanUp.ReferenceOTU9426 | 0 | 1 | 1 | 0 | 0 | 1 | 1 |
| New.CleanUp.ReferenceOTU9552 | 0 | 1 | 0 | 1 | 0 | 0 | 0 |
| New.CleanUp.ReferenceOTU9757 | 0 | 0 | 0 | 1 | 1 | 1 | 1 |
| New.CleanUp.ReferenceOTU9904 | 0 | 1 | 0 | 0 | 0 | 0 | 1 |
| New.CleanUp.ReferenceOTU10310 | 0 | 0 | 0 | 1 | 0 | 1 | 0 |
| New.CleanUp.ReferenceOTU10584 | 0 | 0 | 1 | 1 | 1 | 0 | 1 |
| New.CleanUp.ReferenceOTU10767 | 0 | 1 | 1 | 1 | 1 | 1 | 1 |
| New.CleanUp.ReferenceOTU10777 | 0 | 0 | 0 | 1 | 0 | 1 | 1 |
| New.CleanUp.ReferenceOTU10787 | 0 | 0 | 1 | 0 | 0 | 0 | 0 |
| New.CleanUp.ReferenceOTU11031 | 0 | 1 | 0 | 0 | 0 | 0 | 0 |
| New.CleanUp.ReferenceOTU11544 | 0 | 0 | 1 | 1 | 1 | 1 | 1 |
| New.CleanUp.ReferenceOTU11822 | 0 | 0 | 0 | 1 | 0 | 1 | 0 |
| New.CleanUp.ReferenceOTU11928 | 0 | 0 | 0 | 0 | 0 | 1 | 1 |
| New.CleanUp.ReferenceOTU12221 | 0 | 1 | 0 | 0 | 0 | 0 | 0 |
| New.CleanUp.ReferenceOTU12763 | 0 | 0 | 0 | 0 | 1 | 1 | 0 |
| New.CleanUp.ReferenceOTU14063 | 0 | 1 | 0 | 1 | 0 | 1 | 1 |
| New.CleanUp.ReferenceOTU14417 | 0 | 0 | 0 | 1 | 0 | 1 | 0 |
| New.CleanUp.ReferenceOTU14448 | 0 | 0 | 1 | 0 | 1 | 1 | 0 |
| New.CleanUp.ReferenceOTU14622 | 0 | 1 | 0 | 1 | 1 | 1 | 1 |
| New.CleanUp.ReferenceOTU15366 | 0 | 1 | 0 | 0 | 0 | 1 | 0 |
| New.CleanUp.ReferenceOTU15620 | 0 | 1 | 0 | 1 | 1 | 1 | 1 |
| New.CleanUp.ReferenceOTU16050 | 0 | 0 | 0 | 0 | 0 | 1 | 1 |
| New.CleanUp.ReferenceOTU16156 | 0 | 1 | 0 | 1 | 0 | 1 | 1 |
| New.CleanUp.ReferenceOTU16282 | 0 | 1 | 0 | 1 | 1 | 1 | 1 |
| New.CleanUp.ReferenceOTU16316 | 0 | 1 | 0 | 1 | 0 | 0 | 1 |
| New.CleanUp.ReferenceOTU17141 | 0 | 0 | 0 | 1 | 1 | 0 | 1 |
| New.CleanUp.ReferenceOTU17151 | 0 | 1 | 0 | 1 | 0 | 1 | 1 |
| New.CleanUp.ReferenceOTU17562 | 0 | 1 | 1 | 0 | 0 | 0 | 0 |
| New.CleanUp.ReferenceOTU17722 | 0 | 1 | 1 | 1 | 0 | 0 | 1 |
| New.CleanUp.ReferenceOTU18725 | 0 | 1 | 0 | 1 | 1 | 0 | 0 |
| New.CleanUp.ReferenceOTU18877 | 0 | 1 | 0 | 0 | 0 | 1 | 0 |
| New.CleanUp.ReferenceOTU18900 | 0 | 0 | 0 | 0 | 0 | 1 | 1 |
| New.CleanUp.ReferenceOTU18969 | 0 | 1 | 0 | 1 | 0 | 0 | 0 |
| New.CleanUp.ReferenceOTU19816 | 0 | 0 | 0 | 0 | 1 | 1 | 1 |
| New.CleanUp.ReferenceOTU20204 | 0 | 0 | 0 | 1 | 0 | 1 | 0 |
| New.CleanUp.ReferenceOTU20677 | 0 | 1 | 0 | 1 | 0 | 1 | 1 |
| New.CleanUp.ReferenceOTU20757 | 0 | 1 | 0 | 1 | 1 | 1 | 1 |
| New.CleanUp.ReferenceOTU21075 | 0 | 1 | 0 | 0 | 0 | 1 | 1 |
| New.CleanUp.ReferenceOTU21524 | 0 | 1 | 0 | 0 | 0 | 1 | 0 |
| New.CleanUp.ReferenceOTU21660 | 0 | 0 | 1 | 0 | 1 | 0 | 0 |
| New.CleanUp.ReferenceOTU21694 | 0 | 0 | 0 | 0 | 0 | 1 | 0 |
| New.CleanUp.ReferenceOTU22044 | 0 | 1 | 0 | 1 | 0 | 1 | 0 |
| New.CleanUp.ReferenceOTU23159 | 0 | 1 | 0 | 1 | 0 | 0 | 1 |
| New.CleanUp.ReferenceOTU23300 | 0 | 1 | 0 | 0 | 0 | 0 | 0 |
| New.CleanUp.ReferenceOTU23406 | 0 | 1 | 0 | 1 | 0 | 1 | 0 |
| New.CleanUp.ReferenceOTU23490 | 0 | 0 | 0 | 0 | 1 | 1 | 1 |
| New.CleanUp.ReferenceOTU23639 | 0 | 1 | 1 | 1 | 1 | 1 | 1 |
| New.CleanUp.ReferenceOTU23947 | 0 | 1 | 0 | 1 | 0 | 1 | 1 |
| New.CleanUp.ReferenceOTU24157 | 0 | 0 | 1 | 0 | 0 | 0 | 0 |
| New.CleanUp.ReferenceOTU24209 | 0 | 0 | 1 | 1 | 0 | 0 | 1 |
| New.CleanUp.ReferenceOTU24465 | 0 | 1 | 0 | 1 | 0 | 0 | 0 |
| New.CleanUp.ReferenceOTU25557 | 0 | 0 | 0 | 0 | 0 | 1 | 1 |
| New.CleanUp.ReferenceOTU26097 | 0 | 1 | 1 | 1 | 0 | 1 | 0 |
| New.CleanUp.ReferenceOTU26716 | 0 | 1 | 0 | 0 | 1 | 1 | 1 |
| New.CleanUp.ReferenceOTU28664 | 0 | 1 | 0 | 1 | 0 | 1 | 1 |
| New.CleanUp.ReferenceOTU28684 | 0 | 1 | 0 | 1 | 0 | 0 | 1 |
| New.CleanUp.ReferenceOTU29226 | 0 | 0 | 0 | 0 | 0 | 1 | 1 |
| New.CleanUp.ReferenceOTU29364 | 0 | 1 | 1 | 0 | 0 | 0 | 1 |
| New.CleanUp.ReferenceOTU29598 | 0 | 0 | 0 | 0 | 0 | 1 | 1 |
| New.CleanUp.ReferenceOTU29635 | 0 | 1 | 0 | 1 | 0 | 1 | 1 |
| New.CleanUp.ReferenceOTU29652 | 0 | 1 | 0 | 1 | 0 | 1 | 1 |
| New.CleanUp.ReferenceOTU30389 | 0 | 1 | 1 | 1 | 0 | 0 | 1 |
| New.CleanUp.ReferenceOTU30404 | 0 | 1 | 0 | 1 | 1 | 1 | 1 |
| New.CleanUp.ReferenceOTU30408 | 0 | 0 | 0 | 0 | 1 | 1 | 1 |
| New.CleanUp.ReferenceOTU30767 | 0 | 0 | 0 | 0 | 1 | 1 | 0 |
| New.CleanUp.ReferenceOTU31611 | 0 | 1 | 0 | 1 | 1 | 1 | 0 |
| New.CleanUp.ReferenceOTU31654 | 0 | 0 | 0 | 1 | 1 | 1 | 0 |
| New.CleanUp.ReferenceOTU32223 | 0 | 0 | 0 | 1 | 1 | 0 | 0 |
| New.CleanUp.ReferenceOTU32322 | 0 | 1 | 1 | 1 | 1 | 1 | 1 |
| New.CleanUp.ReferenceOTU32599 | 0 | 0 | 0 | 1 | 0 | 1 | 1 |
| New.CleanUp.ReferenceOTU32783 | 0 | 1 | 0 | 0 | 0 | 1 | 0 |
| New.CleanUp.ReferenceOTU33088 | 0 | 0 | 0 | 1 | 0 | 0 | 1 |
| New.CleanUp.ReferenceOTU33173 | 0 | 1 | 0 | 0 | 0 | 0 | 1 |
| New.CleanUp.ReferenceOTU33332 | 0 | 0 | 0 | 1 | 1 | 1 | 0 |
| New.CleanUp.ReferenceOTU33539 | 0 | 0 | 1 | 0 | 0 | 0 | 0 |
| New.CleanUp.ReferenceOTU34476 | 0 | 1 | 0 | 1 | 0 | 1 | 1 |
| New.CleanUp.ReferenceOTU34502 | 0 | 1 | 0 | 1 | 0 | 1 | 0 |
| New.CleanUp.ReferenceOTU34567 | 0 | 0 | 0 | 0 | 1 | 1 | 1 |
| New.CleanUp.ReferenceOTU34716 | 0 | 0 | 1 | 1 | 0 | 1 | 0 |
| New.CleanUp.ReferenceOTU34736 | 0 | 0 | 0 | 0 | 1 | 1 | 0 |
| New.CleanUp.ReferenceOTU34834 | 0 | 0 | 0 | 1 | 1 | 1 | 0 |
| New.CleanUp.ReferenceOTU35047 | 0 | 1 | 0 | 1 | 0 | 0 | 1 |
| New.CleanUp.ReferenceOTU35076 | 0 | 0 | 0 | 0 | 1 | 0 | 0 |
| New.CleanUp.ReferenceOTU35082 | 0 | 1 | 0 | 1 | 1 | 1 | 1 |
| New.CleanUp.ReferenceOTU35396 | 0 | 0 | 0 | 0 | 0 | 1 | 1 |
| New.CleanUp.ReferenceOTU35791 | 0 | 0 | 0 | 1 | 1 | 1 | 1 |
| New.CleanUp.ReferenceOTU35837 | 0 | 1 | 0 | 0 | 1 | 0 | 0 |
| New.CleanUp.ReferenceOTU35893 | 0 | 0 | 1 | 0 | 1 | 1 | 0 |
| New.CleanUp.ReferenceOTU36484 | 0 | 1 | 0 | 1 | 0 | 1 | 0 |
| New.CleanUp.ReferenceOTU36918 | 0 | 0 | 0 | 0 | 0 | 1 | 1 |
| New.CleanUp.ReferenceOTU37138 | 0 | 1 | 0 | 0 | 0 | 1 | 1 |
| New.CleanUp.ReferenceOTU37141 | 0 | 1 | 0 | 1 | 0 | 0 | 0 |
| New.CleanUp.ReferenceOTU37394 | 0 | 0 | 0 | 1 | 0 | 1 | 0 |
| New.CleanUp.ReferenceOTU37770 | 0 | 1 | 0 | 1 | 0 | 0 | 0 |
| New.CleanUp.ReferenceOTU38122 | 0 | 0 | 1 | 1 | 1 | 1 | 1 |
| New.CleanUp.ReferenceOTU38821 | 0 | 1 | 0 | 1 | 1 | 1 | 1 |
| New.CleanUp.ReferenceOTU38894 | 0 | 0 | 0 | 0 | 0 | 1 | 1 |
| New.CleanUp.ReferenceOTU39832 | 0 | 0 | 0 | 1 | 0 | 1 | 1 |
| New.CleanUp.ReferenceOTU40083 | 0 | 1 | 0 | 1 | 0 | 0 | 1 |
| New.CleanUp.ReferenceOTU40394 | 0 | 0 | 0 | 0 | 0 | 1 | 1 |
| New.CleanUp.ReferenceOTU40689 | 0 | 0 | 0 | 1 | 1 | 1 | 0 |
| New.CleanUp.ReferenceOTU40760 | 0 | 0 | 0 | 1 | 1 | 1 | 0 |
| New.CleanUp.ReferenceOTU41142 | 0 | 0 | 1 | 0 | 0 | 1 | 1 |
| New.CleanUp.ReferenceOTU41561 | 0 | 1 | 1 | 1 | 0 | 0 | 0 |
| New.CleanUp.ReferenceOTU43299 | 1 | 1 | 1 | 1 | 1 | 1 | 1 |
| New.CleanUp.ReferenceOTU43394 | 0 | 0 | 0 | 0 | 0 | 1 | 0 |
| New.CleanUp.ReferenceOTU43956 | 0 | 1 | 1 | 1 | 1 | 1 | 1 |
| New.CleanUp.ReferenceOTU44296 | 0 | 0 | 1 | 1 | 0 | 0 | 1 |
| New.CleanUp.ReferenceOTU44776 | 0 | 0 | 0 | 0 | 1 | 0 | 0 |
| New.CleanUp.ReferenceOTU44820 | 0 | 1 | 0 | 1 | 0 | 1 | 1 |
| New.CleanUp.ReferenceOTU45355 | 0 | 1 | 0 | 1 | 1 | 1 | 1 |
| New.CleanUp.ReferenceOTU45481 | 0 | 1 | 0 | 1 | 0 | 1 | 1 |
| New.CleanUp.ReferenceOTU46268 | 0 | 1 | 0 | 0 | 0 | 0 | 1 |
| New.CleanUp.ReferenceOTU46641 | 0 | 1 | 1 | 1 | 1 | 1 | 1 |
| New.CleanUp.ReferenceOTU47143 | 0 | 0 | 1 | 1 | 1 | 1 | 1 |
| New.CleanUp.ReferenceOTU47379 | 0 | 1 | 1 | 1 | 0 | 1 | 1 |
| New.CleanUp.ReferenceOTU48238 | 0 | 1 | 0 | 0 | 0 | 1 | 0 |
| New.CleanUp.ReferenceOTU48628 | 0 | 1 | 0 | 1 | 0 | 1 | 0 |
| New.CleanUp.ReferenceOTU49119 | 0 | 1 | 0 | 0 | 0 | 1 | 1 |
| New.CleanUp.ReferenceOTU49629 | 0 | 0 | 0 | 0 | 0 | 1 | 0 |
| New.CleanUp.ReferenceOTU49848 | 0 | 0 | 0 | 1 | 0 | 1 | 1 |
| New.CleanUp.ReferenceOTU50064 | 0 | 0 | 0 | 0 | 0 | 1 | 1 |
| New.CleanUp.ReferenceOTU50664 | 0 | 1 | 0 | 0 | 1 | 1 | 1 |
| New.CleanUp.ReferenceOTU50853 | 0 | 1 | 0 | 1 | 0 | 1 | 1 |
| New.CleanUp.ReferenceOTU51091 | 0 | 1 | 0 | 0 | 0 | 0 | 1 |
| New.CleanUp.ReferenceOTU52260 | 0 | 0 | 0 | 1 | 0 | 0 | 1 |
| New.CleanUp.ReferenceOTU52842 | 0 | 1 | 0 | 1 | 0 | 1 | 1 |
| New.CleanUp.ReferenceOTU52902 | 0 | 1 | 0 | 1 | 0 | 0 | 1 |
| New.CleanUp.ReferenceOTU53205 | 0 | 0 | 0 | 0 | 0 | 1 | 1 |
| New.CleanUp.ReferenceOTU53275 | 0 | 0 | 0 | 0 | 0 | 1 | 0 |
| New.CleanUp.ReferenceOTU53575 | 0 | 1 | 0 | 1 | 0 | 1 | 1 |
| New.CleanUp.ReferenceOTU53919 | 0 | 0 | 0 | 1 | 0 | 1 | 1 |
| New.CleanUp.ReferenceOTU54056 | 0 | 0 | 0 | 0 | 1 | 1 | 1 |
| New.CleanUp.ReferenceOTU54877 | 0 | 0 | 0 | 1 | 0 | 1 | 0 |
| New.CleanUp.ReferenceOTU55022 | 0 | 1 | 0 | 1 | 0 | 1 | 1 |
| New.CleanUp.ReferenceOTU55741 | 0 | 0 | 0 | 1 | 1 | 1 | 1 |
| New.CleanUp.ReferenceOTU56112 | 0 | 0 | 0 | 0 | 1 | 0 | 0 |
| New.CleanUp.ReferenceOTU56163 | 0 | 1 | 1 | 1 | 1 | 1 | 1 |
| New.CleanUp.ReferenceOTU56384 | 0 | 0 | 0 | 1 | 0 | 1 | 0 |
| New.CleanUp.ReferenceOTU56392 | 0 | 1 | 0 | 1 | 1 | 1 | 1 |
| New.CleanUp.ReferenceOTU57072 | 0 | 0 | 0 | 0 | 1 | 1 | 0 |
| New.CleanUp.ReferenceOTU57258 | 0 | 0 | 0 | 0 | 1 | 0 | 0 |
| New.CleanUp.ReferenceOTU57322 | 0 | 1 | 0 | 1 | 1 | 1 | 0 |
| New.CleanUp.ReferenceOTU57829 | 0 | 1 | 0 | 0 | 0 | 0 | 0 |
| New.CleanUp.ReferenceOTU58178 | 0 | 1 | 0 | 1 | 1 | 0 | 0 |
| New.CleanUp.ReferenceOTU58264 | 0 | 1 | 0 | 0 | 1 | 0 | 0 |
| New.CleanUp.ReferenceOTU58516 | 0 | 0 | 1 | 0 | 1 | 0 | 0 |
| New.CleanUp.ReferenceOTU58862 | 0 | 1 | 0 | 0 | 0 | 1 | 0 |
| New.CleanUp.ReferenceOTU58885 | 0 | 0 | 0 | 1 | 0 | 1 | 0 |
| New.CleanUp.ReferenceOTU59087 | 0 | 1 | 0 | 0 | 0 | 1 | 0 |
| New.CleanUp.ReferenceOTU59239 | 0 | 0 | 1 | 1 | 0 | 1 | 0 |
| New.CleanUp.ReferenceOTU59322 | 0 | 1 | 0 | 0 | 0 | 0 | 1 |
| New.CleanUp.ReferenceOTU59542 | 0 | 0 | 1 | 0 | 1 | 0 | 0 |
| New.CleanUp.ReferenceOTU59543 | 0 | 0 | 0 | 0 | 0 | 1 | 1 |
| New.CleanUp.ReferenceOTU59735 | 0 | 1 | 1 | 0 | 0 | 1 | 1 |
| New.CleanUp.ReferenceOTU59840 | 0 | 1 | 0 | 1 | 1 | 0 | 1 |
| New.CleanUp.ReferenceOTU60463 | 0 | 0 | 0 | 0 | 1 | 1 | 0 |
| New.CleanUp.ReferenceOTU60706 | 0 | 1 | 1 | 1 | 0 | 0 | 0 |
| New.CleanUp.ReferenceOTU60762 | 0 | 0 | 0 | 0 | 0 | 1 | 1 |
| New.CleanUp.ReferenceOTU61553 | 0 | 1 | 1 | 1 | 1 | 1 | 1 |
| New.CleanUp.ReferenceOTU61643 | 0 | 1 | 0 | 0 | 0 | 1 | 1 |
| New.CleanUp.ReferenceOTU61929 | 0 | 1 | 1 | 1 | 1 | 1 | 1 |
| New.CleanUp.ReferenceOTU62246 | 0 | 1 | 0 | 0 | 0 | 1 | 0 |
| New.CleanUp.ReferenceOTU62270 | 0 | 0 | 0 | 1 | 0 | 1 | 1 |
| New.CleanUp.ReferenceOTU62558 | 0 | 1 | 1 | 1 | 0 | 0 | 1 |
| New.CleanUp.ReferenceOTU62807 | 0 | 0 | 0 | 0 | 0 | 1 | 0 |
| New.CleanUp.ReferenceOTU62902 | 0 | 0 | 0 | 1 | 1 | 1 | 0 |
| New.CleanUp.ReferenceOTU62959 | 0 | 1 | 0 | 0 | 1 | 1 | 1 |
| New.CleanUp.ReferenceOTU65068 | 0 | 1 | 1 | 1 | 1 | 1 | 1 |
| New.CleanUp.ReferenceOTU65909 | 0 | 0 | 0 | 1 | 0 | 1 | 1 |
| New.CleanUp.ReferenceOTU65998 | 0 | 0 | 0 | 1 | 0 | 0 | 1 |
| New.CleanUp.ReferenceOTU66330 | 1 | 1 | 1 | 1 | 1 | 1 | 1 |
| New.CleanUp.ReferenceOTU66477 | 0 | 0 | 0 | 1 | 1 | 0 | 0 |
| New.CleanUp.ReferenceOTU67158 | 0 | 0 | 0 | 1 | 1 | 1 | 0 |
| New.CleanUp.ReferenceOTU67323 | 0 | 1 | 1 | 1 | 0 | 0 | 0 |
| New.CleanUp.ReferenceOTU67731 | 0 | 1 | 0 | 0 | 0 | 0 | 1 |
| New.CleanUp.ReferenceOTU68146 | 0 | 0 | 0 | 0 | 0 | 1 | 0 |
| New.CleanUp.ReferenceOTU68787 | 0 | 1 | 0 | 0 | 1 | 0 | 0 |
| New.CleanUp.ReferenceOTU69015 | 0 | 0 | 0 | 1 | 0 | 1 | 0 |
| New.CleanUp.ReferenceOTU69656 | 0 | 1 | 1 | 1 | 1 | 1 | 1 |
| New.CleanUp.ReferenceOTU69883 | 0 | 1 | 0 | 0 | 0 | 1 | 1 |
| New.CleanUp.ReferenceOTU70022 | 0 | 1 | 0 | 1 | 0 | 0 | 0 |
| New.CleanUp.ReferenceOTU70186 | 0 | 1 | 0 | 1 | 1 | 1 | 1 |
| New.CleanUp.ReferenceOTU70538 | 0 | 0 | 1 | 0 | 0 | 1 | 1 |
| New.CleanUp.ReferenceOTU70978 | 0 | 0 | 0 | 1 | 1 | 1 | 1 |
| New.CleanUp.ReferenceOTU72251 | 0 | 0 | 0 | 1 | 1 | 1 | 1 |
| New.CleanUp.ReferenceOTU72531 | 0 | 1 | 0 | 0 | 0 | 0 | 0 |
| New.CleanUp.ReferenceOTU72763 | 0 | 1 | 0 | 0 | 0 | 1 | 1 |
| New.CleanUp.ReferenceOTU72951 | 0 | 1 | 0 | 1 | 0 | 0 | 1 |
| New.CleanUp.ReferenceOTU73711 | 1 | 1 | 0 | 0 | 1 | 1 | 0 |
| New.CleanUp.ReferenceOTU74223 | 0 | 1 | 1 | 0 | 1 | 1 | 1 |
| New.CleanUp.ReferenceOTU74667 | 0 | 0 | 1 | 1 | 1 | 1 | 1 |
| New.CleanUp.ReferenceOTU74730 | 0 | 1 | 0 | 1 | 0 | 0 | 0 |
| New.CleanUp.ReferenceOTU74937 | 0 | 1 | 0 | 1 | 0 | 1 | 0 |
| New.CleanUp.ReferenceOTU74943 | 0 | 1 | 0 | 1 | 1 | 1 | 1 |
| New.CleanUp.ReferenceOTU75758 | 0 | 1 | 0 | 0 | 1 | 1 | 0 |
| New.CleanUp.ReferenceOTU76094 | 0 | 1 | 0 | 1 | 0 | 1 | 1 |
| New.CleanUp.ReferenceOTU76445 | 0 | 1 | 1 | 1 | 1 | 1 | 1 |
| New.CleanUp.ReferenceOTU76457 | 0 | 1 | 0 | 0 | 0 | 0 | 1 |
| New.CleanUp.ReferenceOTU76505 | 0 | 0 | 0 | 1 | 0 | 1 | 0 |
| New.CleanUp.ReferenceOTU76883 | 0 | 1 | 1 | 1 | 0 | 1 | 0 |
| New.CleanUp.ReferenceOTU77421 | 0 | 0 | 1 | 0 | 0 | 1 | 1 |
| New.CleanUp.ReferenceOTU77793 | 1 | 0 | 0 | 0 | 1 | 0 | 0 |
| New.CleanUp.ReferenceOTU78005 | 0 | 0 | 0 | 1 | 0 | 1 | 1 |
| New.CleanUp.ReferenceOTU78151 | 0 | 1 | 0 | 1 | 1 | 0 | 0 |
| New.CleanUp.ReferenceOTU78936 | 0 | 1 | 1 | 1 | 0 | 1 | 0 |
| New.CleanUp.ReferenceOTU78945 | 0 | 1 | 0 | 0 | 0 | 1 | 1 |
| New.CleanUp.ReferenceOTU79304 | 0 | 1 | 0 | 0 | 0 | 1 | 1 |
| New.CleanUp.ReferenceOTU80872 | 0 | 1 | 0 | 1 | 0 | 1 | 0 |
| New.CleanUp.ReferenceOTU3039 | 1 | 1 | 1 | 1 | 1 | 0 | 0 |
| New.CleanUp.ReferenceOTU3067 | 1 | 0 | 1 | 0 | 1 | 0 | 0 |
| New.CleanUp.ReferenceOTU15128 | 0 | 0 | 0 | 1 | 1 | 0 | 0 |
| New.CleanUp.ReferenceOTU15130 | 1 | 0 | 0 | 0 | 0 | 0 | 0 |
| New.CleanUp.ReferenceOTU61412 | 0 | 0 | 0 | 0 | 1 | 0 | 0 |
| New.CleanUp.ReferenceOTU66622 | 0 | 1 | 0 | 0 | 1 | 0 | 0 |
| KT346265.1.1782 | 0 | 1 | 0 | 1 | 0 | 1 | 0 |
| New.CleanUp.ReferenceOTU14205 | 0 | 1 | 0 | 1 | 0 | 1 | 0 |
| New.CleanUp.ReferenceOTU41752 | 0 | 1 | 0 | 1 | 0 | 1 | 0 |
| New.CleanUp.ReferenceOTU75727 | 0 | 0 | 0 | 0 | 0 | 1 | 0 |
| New.CleanUp.ReferenceOTU21497 | 0 | 0 | 0 | 0 | 0 | 1 | 0 |
| New.CleanUp.ReferenceOTU60131 | 0 | 0 | 0 | 0 | 0 | 1 | 0 |
| New.CleanUp.ReferenceOTU42388 | 0 | 0 | 0 | 0 | 0 | 1 | 1 |
| New.CleanUp.ReferenceOTU71159 | 0 | 1 | 0 | 0 | 0 | 1 | 1 |
| FR865625.2.1771 | 0 | 1 | 0 | 1 | 0 | 1 | 0 |
| New.CleanUp.ReferenceOTU25778 | 0 | 1 | 0 | 1 | 0 | 1 | 0 |
| New.CleanUp.ReferenceOTU57431 | 0 | 1 | 0 | 0 | 0 | 1 | 0 |
| AF022155.1.1790 | 0 | 0 | 0 | 0 | 1 | 0 | 0 |
| New.ReferenceOTU186 | 1 | 1 | 0 | 1 | 1 | 1 | 1 |
| New.CleanUp.ReferenceOTU9253 | 1 | 1 | 0 | 1 | 1 | 1 | 1 |
| New.CleanUp.ReferenceOTU74415 | 0 | 0 | 0 | 0 | 1 | 1 | 0 |
| New.CleanUp.ReferenceOTU26787 | 0 | 0 | 0 | 1 | 0 | 0 | 0 |
| New.CleanUp.ReferenceOTU37122 | 0 | 0 | 0 | 1 | 0 | 0 | 0 |
| New.CleanUp.ReferenceOTU51492 | 0 | 0 | 0 | 1 | 0 | 0 | 0 |
| New.CleanUp.ReferenceOTU54291 | 1 | 0 | 0 | 0 | 0 | 0 | 0 |
| AY831410.381.2182 | 0 | 0 | 0 | 1 | 0 | 1 | 0 |
| New.CleanUp.ReferenceOTU1843 | 0 | 0 | 0 | 1 | 0 | 0 | 0 |
| New.CleanUp.ReferenceOTU8807 | 0 | 0 | 0 | 1 | 0 | 1 | 0 |
| New.CleanUp.ReferenceOTU70236 | 0 | 0 | 0 | 0 | 0 | 1 | 0 |
| New.CleanUp.ReferenceOTU76635 | 0 | 0 | 0 | 0 | 0 | 1 | 0 |
| AY347309.334.2135 | 1 | 1 | 0 | 1 | 0 | 1 | 1 |
| New.CleanUp.ReferenceOTU6898 | 1 | 1 | 0 | 1 | 0 | 1 | 1 |
| New.CleanUp.ReferenceOTU34493 | 1 | 1 | 0 | 1 | 0 | 0 | 1 |
| New.CleanUp.ReferenceOTU78117 | 0 | 0 | 0 | 1 | 0 | 0 | 0 |
| New.CleanUp.ReferenceOTU13211 | 0 | 1 | 0 | 0 | 0 | 0 | 0 |
| New.CleanUp.ReferenceOTU42725 | 0 | 1 | 1 | 1 | 1 | 1 | 0 |
| New.CleanUp.ReferenceOTU79927 | 0 | 0 | 0 | 0 | 0 | 1 | 0 |
| KJ758298.1.1810 | 0 | 1 | 1 | 1 | 1 | 1 | 0 |
| New.CleanUp.ReferenceOTU10679 | 0 | 1 | 0 | 1 | 1 | 1 | 0 |
| New.CleanUp.ReferenceOTU48472 | 0 | 1 | 0 | 0 | 1 | 1 | 0 |
| New.CleanUp.ReferenceOTU70263 | 0 | 1 | 0 | 1 | 1 | 1 | 0 |
| DQ371292.1.1755 | 1 | 1 | 1 | 1 | 0 | 1 | 1 |
| New.ReferenceOTU294 | 1 | 1 | 1 | 1 | 0 | 1 | 1 |
| New.ReferenceOTU202 | 1 | 0 | 1 | 0 | 0 | 1 | 1 |
| New.CleanUp.ReferenceOTU348 | 1 | 0 | 0 | 0 | 0 | 1 | 0 |
| New.CleanUp.ReferenceOTU1217 | 0 | 1 | 0 | 0 | 0 | 1 | 1 |
| New.CleanUp.ReferenceOTU1484 | 0 | 0 | 0 | 0 | 0 | 1 | 0 |
| New.CleanUp.ReferenceOTU2012 | 0 | 0 | 0 | 0 | 0 | 1 | 0 |
| New.CleanUp.ReferenceOTU3240 | 0 | 0 | 0 | 0 | 0 | 1 | 0 |
| New.CleanUp.ReferenceOTU3823 | 0 | 0 | 0 | 0 | 0 | 1 | 0 |
| New.CleanUp.ReferenceOTU4976 | 0 | 0 | 0 | 0 | 0 | 1 | 1 |
| New.CleanUp.ReferenceOTU8992 | 0 | 1 | 0 | 0 | 0 | 1 | 0 |
| New.CleanUp.ReferenceOTU9385 | 0 | 0 | 0 | 0 | 0 | 1 | 0 |
| New.CleanUp.ReferenceOTU9634 | 0 | 0 | 0 | 0 | 0 | 1 | 0 |
| New.CleanUp.ReferenceOTU10504 | 0 | 0 | 0 | 0 | 0 | 0 | 1 |
| New.CleanUp.ReferenceOTU11358 | 0 | 0 | 0 | 0 | 0 | 1 | 1 |
| New.CleanUp.ReferenceOTU12547 | 0 | 0 | 0 | 0 | 0 | 1 | 0 |
| New.CleanUp.ReferenceOTU12772 | 0 | 0 | 1 | 0 | 0 | 1 | 0 |
| New.CleanUp.ReferenceOTU12813 | 0 | 0 | 0 | 0 | 0 | 1 | 0 |
| New.CleanUp.ReferenceOTU12971 | 0 | 0 | 0 | 0 | 0 | 1 | 0 |
| New.CleanUp.ReferenceOTU14057 | 0 | 0 | 0 | 0 | 0 | 1 | 1 |
| New.CleanUp.ReferenceOTU17327 | 0 | 0 | 0 | 0 | 0 | 1 | 0 |
| New.CleanUp.ReferenceOTU17340 | 0 | 0 | 0 | 0 | 0 | 1 | 0 |
| New.CleanUp.ReferenceOTU17589 | 0 | 0 | 0 | 0 | 0 | 1 | 1 |
| New.CleanUp.ReferenceOTU17945 | 0 | 0 | 0 | 0 | 0 | 1 | 0 |
| New.CleanUp.ReferenceOTU18656 | 0 | 0 | 0 | 0 | 0 | 1 | 0 |
| New.CleanUp.ReferenceOTU21394 | 0 | 0 | 0 | 0 | 0 | 1 | 0 |
| New.CleanUp.ReferenceOTU22321 | 0 | 0 | 0 | 0 | 0 | 1 | 0 |
| New.CleanUp.ReferenceOTU24506 | 0 | 0 | 0 | 0 | 0 | 1 | 0 |
| New.CleanUp.ReferenceOTU24601 | 0 | 0 | 0 | 0 | 0 | 1 | 0 |
| New.CleanUp.ReferenceOTU24833 | 0 | 1 | 0 | 0 | 0 | 1 | 0 |
| New.CleanUp.ReferenceOTU25393 | 0 | 0 | 0 | 0 | 0 | 1 | 0 |
| New.CleanUp.ReferenceOTU26279 | 0 | 0 | 0 | 0 | 0 | 1 | 0 |
| New.CleanUp.ReferenceOTU26490 | 0 | 0 | 0 | 0 | 0 | 1 | 0 |
| New.CleanUp.ReferenceOTU27979 | 0 | 0 | 0 | 0 | 0 | 1 | 0 |
| New.CleanUp.ReferenceOTU30239 | 0 | 0 | 0 | 0 | 0 | 1 | 0 |
| New.CleanUp.ReferenceOTU31609 | 0 | 0 | 1 | 0 | 0 | 1 | 1 |
| New.CleanUp.ReferenceOTU32290 | 0 | 0 | 0 | 0 | 0 | 1 | 1 |
| New.CleanUp.ReferenceOTU37324 | 0 | 0 | 0 | 0 | 0 | 1 | 0 |
| New.CleanUp.ReferenceOTU38237 | 0 | 0 | 0 | 0 | 0 | 1 | 0 |
| New.CleanUp.ReferenceOTU39404 | 0 | 0 | 0 | 0 | 0 | 1 | 0 |
| New.CleanUp.ReferenceOTU39513 | 0 | 0 | 0 | 0 | 0 | 1 | 0 |
| New.CleanUp.ReferenceOTU40207 | 0 | 0 | 0 | 0 | 0 | 1 | 0 |
| New.CleanUp.ReferenceOTU42627 | 0 | 0 | 0 | 0 | 0 | 1 | 0 |
| New.CleanUp.ReferenceOTU42726 | 0 | 0 | 0 | 0 | 0 | 1 | 0 |
| New.CleanUp.ReferenceOTU43724 | 0 | 0 | 0 | 0 | 0 | 1 | 0 |
| New.CleanUp.ReferenceOTU45388 | 0 | 0 | 0 | 0 | 0 | 0 | 1 |
| New.CleanUp.ReferenceOTU45982 | 0 | 0 | 0 | 0 | 0 | 1 | 0 |
| New.CleanUp.ReferenceOTU46575 | 0 | 0 | 0 | 0 | 0 | 1 | 0 |
| New.CleanUp.ReferenceOTU46832 | 0 | 0 | 0 | 0 | 0 | 1 | 0 |
| New.CleanUp.ReferenceOTU46963 | 0 | 0 | 0 | 0 | 0 | 1 | 0 |
| New.CleanUp.ReferenceOTU47461 | 0 | 0 | 0 | 0 | 0 | 1 | 0 |
| New.CleanUp.ReferenceOTU49577 | 0 | 0 | 0 | 0 | 0 | 1 | 0 |
| New.CleanUp.ReferenceOTU50450 | 0 | 1 | 0 | 0 | 0 | 1 | 0 |
| New.CleanUp.ReferenceOTU50917 | 0 | 0 | 0 | 0 | 0 | 1 | 0 |
| New.CleanUp.ReferenceOTU50969 | 0 | 0 | 0 | 0 | 0 | 1 | 0 |
| New.CleanUp.ReferenceOTU51990 | 0 | 0 | 0 | 0 | 0 | 1 | 0 |
| New.CleanUp.ReferenceOTU52741 | 0 | 0 | 0 | 0 | 0 | 1 | 0 |
| New.CleanUp.ReferenceOTU54552 | 0 | 0 | 0 | 0 | 0 | 1 | 0 |
| New.CleanUp.ReferenceOTU55006 | 0 | 0 | 0 | 0 | 0 | 1 | 0 |
| New.CleanUp.ReferenceOTU55029 | 0 | 0 | 0 | 0 | 0 | 1 | 0 |
| New.CleanUp.ReferenceOTU57267 | 0 | 0 | 0 | 0 | 0 | 1 | 0 |
| New.CleanUp.ReferenceOTU58244 | 0 | 0 | 0 | 0 | 0 | 1 | 0 |
| New.CleanUp.ReferenceOTU58542 | 0 | 0 | 0 | 0 | 0 | 1 | 0 |
| New.CleanUp.ReferenceOTU60553 | 0 | 0 | 0 | 0 | 0 | 1 | 0 |
| New.CleanUp.ReferenceOTU62556 | 0 | 0 | 0 | 0 | 0 | 1 | 0 |
| New.CleanUp.ReferenceOTU62925 | 0 | 0 | 0 | 0 | 0 | 1 | 0 |
| New.CleanUp.ReferenceOTU63298 | 0 | 0 | 1 | 0 | 0 | 1 | 1 |
| New.CleanUp.ReferenceOTU63966 | 0 | 0 | 0 | 0 | 0 | 1 | 0 |
| New.CleanUp.ReferenceOTU64911 | 0 | 0 | 0 | 0 | 0 | 1 | 0 |
| New.CleanUp.ReferenceOTU66046 | 0 | 0 | 0 | 0 | 0 | 1 | 0 |
| New.CleanUp.ReferenceOTU66174 | 0 | 0 | 0 | 0 | 0 | 1 | 0 |
| New.CleanUp.ReferenceOTU66802 | 0 | 0 | 0 | 0 | 0 | 1 | 0 |
| New.CleanUp.ReferenceOTU67966 | 0 | 0 | 0 | 0 | 0 | 1 | 0 |
| New.CleanUp.ReferenceOTU68548 | 0 | 0 | 0 | 0 | 0 | 1 | 0 |
| New.CleanUp.ReferenceOTU69013 | 0 | 0 | 0 | 0 | 0 | 1 | 0 |
| New.CleanUp.ReferenceOTU69052 | 0 | 0 | 0 | 0 | 0 | 1 | 0 |
| New.CleanUp.ReferenceOTU69198 | 0 | 0 | 0 | 0 | 0 | 1 | 0 |
| New.CleanUp.ReferenceOTU70542 | 0 | 0 | 0 | 0 | 0 | 1 | 0 |
| New.CleanUp.ReferenceOTU72114 | 0 | 0 | 0 | 0 | 0 | 1 | 0 |
| New.CleanUp.ReferenceOTU72395 | 0 | 0 | 0 | 0 | 0 | 1 | 0 |
| New.CleanUp.ReferenceOTU74237 | 0 | 0 | 0 | 0 | 0 | 1 | 0 |
| New.CleanUp.ReferenceOTU76602 | 0 | 0 | 0 | 0 | 0 | 1 | 0 |
| New.CleanUp.ReferenceOTU77607 | 0 | 0 | 0 | 0 | 0 | 1 | 0 |
| New.CleanUp.ReferenceOTU79429 | 0 | 0 | 0 | 0 | 0 | 1 | 0 |
| New.CleanUp.ReferenceOTU80333 | 0 | 0 | 0 | 0 | 0 | 1 | 1 |
| New.CleanUp.ReferenceOTU34490 | 0 | 0 | 0 | 0 | 0 | 1 | 0 |
| New.CleanUp.ReferenceOTU37441 | 0 | 0 | 0 | 0 | 0 | 1 | 0 |
| New.CleanUp.ReferenceOTU23140 | 0 | 1 | 0 | 0 | 0 | 1 | 0 |
| New.CleanUp.ReferenceOTU49305 | 0 | 0 | 0 | 0 | 0 | 1 | 0 |
| New.CleanUp.ReferenceOTU74179 | 0 | 0 | 0 | 0 | 0 | 1 | 0 |
| New.CleanUp.ReferenceOTU52604 | 0 | 1 | 0 | 1 | 1 | 1 | 0 |
| New.CleanUp.ReferenceOTU74403 | 0 | 0 | 0 | 0 | 1 | 0 | 0 |
| New.ReferenceOTU185 | 0 | 0 | 0 | 0 | 0 | 1 | 0 |
| New.CleanUp.ReferenceOTU5968 | 0 | 0 | 0 | 0 | 0 | 1 | 0 |
| New.CleanUp.ReferenceOTU48573 | 0 | 0 | 0 | 1 | 0 | 0 | 0 |
| New.CleanUp.ReferenceOTU71865 | 0 | 0 | 0 | 1 | 0 | 0 | 0 |
| New.CleanUp.ReferenceOTU18986 | 0 | 1 | 0 | 0 | 0 | 0 | 0 |
| New.CleanUp.ReferenceOTU30416 | 1 | 1 | 0 | 1 | 0 | 0 | 1 |
| New.CleanUp.ReferenceOTU51022 | 0 | 0 | 0 | 0 | 0 | 0 | 1 |
| New.CleanUp.ReferenceOTU71515 | 0 | 0 | 0 | 0 | 0 | 0 | 1 |
| AF009217.1.1800 | 1 | 1 | 0 | 1 | 0 | 0 | 0 |
| EU046338.1.1759 | 0 | 1 | 0 | 0 | 0 | 0 | 0 |
| EU046337.1.1747 | 1 | 1 | 0 | 1 | 0 | 0 | 0 |
| JN098201.1.1537 | 1 | 1 | 1 | 1 | 1 | 1 | 0 |
| FJ000195.1.1389 | 1 | 1 | 1 | 1 | 1 | 0 | 0 |
| KJ758919.1.1802 | 0 | 0 | 1 | 0 | 1 | 0 | 1 |
| KP790163.1.1798 | 1 | 1 | 1 | 1 | 1 | 0 | 0 |
| JF791102.1.1777 | 1 | 1 | 1 | 1 | 1 | 1 | 1 |
| KJ763897.1.1800 | 1 | 1 | 1 | 1 | 1 | 1 | 1 |
| KJ763153.1.1801 | 0 | 1 | 0 | 1 | 1 | 1 | 0 |
| KJ757982.1.1803 | 1 | 1 | 1 | 0 | 1 | 0 | 0 |
| KJ757821.1.1800 | 0 | 0 | 0 | 0 | 1 | 1 | 0 |
| JF826352.1.1611 | 0 | 1 | 0 | 0 | 1 | 0 | 0 |
| New.ReferenceOTU105 | 1 | 1 | 1 | 1 | 1 | 1 | 1 |
| New.ReferenceOTU128 | 1 | 1 | 1 | 0 | 1 | 1 | 0 |
| New.ReferenceOTU162 | 1 | 1 | 1 | 1 | 1 | 1 | 1 |
| New.CleanUp.ReferenceOTU1159 | 1 | 1 | 0 | 0 | 1 | 1 | 1 |
| New.CleanUp.ReferenceOTU1687 | 0 | 0 | 0 | 0 | 1 | 1 | 0 |
| New.CleanUp.ReferenceOTU2035 | 0 | 0 | 0 | 0 | 1 | 0 | 0 |
| New.CleanUp.ReferenceOTU2687 | 0 | 1 | 0 | 0 | 1 | 0 | 0 |
| New.CleanUp.ReferenceOTU2720 | 1 | 0 | 1 | 0 | 1 | 1 | 0 |
| New.CleanUp.ReferenceOTU2907 | 0 | 0 | 0 | 0 | 1 | 0 | 0 |
| New.CleanUp.ReferenceOTU3154 | 0 | 0 | 0 | 0 | 0 | 1 | 0 |
| New.CleanUp.ReferenceOTU4030 | 0 | 1 | 0 | 0 | 1 | 0 | 0 |
| New.CleanUp.ReferenceOTU5947 | 0 | 0 | 0 | 0 | 0 | 1 | 1 |
| New.CleanUp.ReferenceOTU8818 | 0 | 1 | 0 | 0 | 1 | 0 | 0 |
| New.CleanUp.ReferenceOTU9190 | 0 | 1 | 0 | 0 | 0 | 1 | 0 |
| New.CleanUp.ReferenceOTU10703 | 1 | 0 | 0 | 1 | 0 | 0 | 1 |
| New.CleanUp.ReferenceOTU11437 | 0 | 1 | 0 | 0 | 0 | 0 | 0 |
| New.CleanUp.ReferenceOTU12583 | 1 | 1 | 0 | 1 | 1 | 0 | 0 |
| New.CleanUp.ReferenceOTU12998 | 0 | 1 | 0 | 0 | 1 | 0 | 0 |
| New.CleanUp.ReferenceOTU14435 | 0 | 1 | 0 | 0 | 0 | 1 | 0 |
| New.CleanUp.ReferenceOTU16121 | 0 | 1 | 0 | 1 | 0 | 1 | 0 |
| New.CleanUp.ReferenceOTU17017 | 0 | 1 | 0 | 0 | 1 | 0 | 0 |
| New.CleanUp.ReferenceOTU17085 | 0 | 1 | 0 | 0 | 0 | 0 | 0 |
| New.CleanUp.ReferenceOTU17153 | 0 | 0 | 1 | 1 | 1 | 0 | 0 |
| New.CleanUp.ReferenceOTU17595 | 0 | 1 | 0 | 1 | 1 | 1 | 0 |
| New.CleanUp.ReferenceOTU18636 | 1 | 0 | 0 | 0 | 1 | 0 | 0 |
| New.CleanUp.ReferenceOTU18850 | 0 | 1 | 0 | 0 | 1 | 0 | 0 |
| New.CleanUp.ReferenceOTU21726 | 0 | 1 | 1 | 1 | 1 | 1 | 1 |
| New.CleanUp.ReferenceOTU22662 | 0 | 1 | 0 | 1 | 1 | 1 | 0 |
| New.CleanUp.ReferenceOTU24156 | 0 | 1 | 0 | 0 | 1 | 1 | 0 |
| New.CleanUp.ReferenceOTU25466 | 0 | 0 | 0 | 1 | 1 | 1 | 0 |
| New.CleanUp.ReferenceOTU26677 | 0 | 1 | 1 | 0 | 1 | 0 | 0 |
| New.CleanUp.ReferenceOTU28044 | 0 | 1 | 0 | 1 | 0 | 1 | 0 |
| New.CleanUp.ReferenceOTU29355 | 1 | 1 | 1 | 1 | 1 | 1 | 0 |
| New.CleanUp.ReferenceOTU31160 | 1 | 1 | 1 | 1 | 1 | 0 | 0 |
| New.CleanUp.ReferenceOTU32325 | 1 | 1 | 0 | 0 | 1 | 0 | 0 |
| New.CleanUp.ReferenceOTU36332 | 0 | 1 | 0 | 0 | 1 | 0 | 0 |
| New.CleanUp.ReferenceOTU37092 | 1 | 1 | 0 | 0 | 0 | 1 | 0 |
| New.CleanUp.ReferenceOTU37241 | 0 | 0 | 1 | 1 | 0 | 0 | 0 |
| New.CleanUp.ReferenceOTU37608 | 1 | 1 | 1 | 1 | 1 | 1 | 0 |
| New.CleanUp.ReferenceOTU38378 | 0 | 0 | 0 | 1 | 1 | 0 | 0 |
| New.CleanUp.ReferenceOTU38409 | 0 | 1 | 0 | 0 | 1 | 0 | 0 |
| New.CleanUp.ReferenceOTU41148 | 0 | 1 | 1 | 1 | 1 | 0 | 0 |
| New.CleanUp.ReferenceOTU42158 | 0 | 1 | 0 | 0 | 1 | 0 | 0 |
| New.CleanUp.ReferenceOTU42513 | 0 | 1 | 0 | 1 | 1 | 1 | 1 |
| New.CleanUp.ReferenceOTU44765 | 0 | 1 | 1 | 0 | 1 | 0 | 0 |
| New.CleanUp.ReferenceOTU45074 | 0 | 0 | 0 | 0 | 1 | 0 | 0 |
| New.CleanUp.ReferenceOTU47223 | 0 | 1 | 0 | 0 | 1 | 1 | 0 |
| New.CleanUp.ReferenceOTU48955 | 0 | 0 | 0 | 1 | 1 | 0 | 0 |
| New.CleanUp.ReferenceOTU50947 | 0 | 1 | 0 | 1 | 0 | 1 | 1 |
| New.CleanUp.ReferenceOTU50973 | 0 | 0 | 0 | 0 | 1 | 1 | 0 |
| New.CleanUp.ReferenceOTU52161 | 0 | 0 | 0 | 0 | 1 | 0 | 0 |
| New.CleanUp.ReferenceOTU52701 | 0 | 0 | 1 | 0 | 0 | 0 | 0 |
| New.CleanUp.ReferenceOTU52839 | 0 | 0 | 0 | 0 | 1 | 0 | 0 |
| New.CleanUp.ReferenceOTU54045 | 0 | 1 | 0 | 0 | 1 | 1 | 0 |
| New.CleanUp.ReferenceOTU54259 | 0 | 0 | 0 | 1 | 1 | 1 | 0 |
| New.CleanUp.ReferenceOTU56498 | 0 | 1 | 0 | 0 | 1 | 1 | 0 |
| New.CleanUp.ReferenceOTU56898 | 0 | 1 | 0 | 1 | 1 | 1 | 1 |
| New.CleanUp.ReferenceOTU56935 | 0 | 0 | 0 | 0 | 1 | 1 | 0 |
| New.CleanUp.ReferenceOTU57704 | 0 | 1 | 1 | 0 | 1 | 0 | 0 |
| New.CleanUp.ReferenceOTU58024 | 0 | 1 | 0 | 0 | 1 | 1 | 0 |
| New.CleanUp.ReferenceOTU58403 | 1 | 1 | 0 | 0 | 1 | 1 | 0 |
| New.CleanUp.ReferenceOTU59888 | 0 | 1 | 0 | 1 | 0 | 0 | 0 |
| New.CleanUp.ReferenceOTU60938 | 0 | 1 | 0 | 0 | 1 | 0 | 0 |
| New.CleanUp.ReferenceOTU62021 | 0 | 1 | 1 | 1 | 0 | 0 | 0 |
| New.CleanUp.ReferenceOTU62265 | 0 | 0 | 0 | 1 | 0 | 1 | 0 |
| New.CleanUp.ReferenceOTU62346 | 0 | 1 | 0 | 0 | 1 | 1 | 0 |
| New.CleanUp.ReferenceOTU63039 | 0 | 1 | 1 | 0 | 1 | 0 | 0 |
| New.CleanUp.ReferenceOTU63610 | 0 | 0 | 0 | 0 | 1 | 0 | 0 |
| New.CleanUp.ReferenceOTU65516 | 0 | 0 | 0 | 0 | 1 | 1 | 0 |
| New.CleanUp.ReferenceOTU66450 | 1 | 0 | 0 | 0 | 1 | 0 | 0 |
| New.CleanUp.ReferenceOTU68214 | 0 | 1 | 0 | 1 | 1 | 0 | 0 |
| New.CleanUp.ReferenceOTU69229 | 0 | 1 | 1 | 1 | 0 | 1 | 0 |
| New.CleanUp.ReferenceOTU72122 | 0 | 1 | 1 | 1 | 1 | 1 | 0 |
| New.CleanUp.ReferenceOTU72243 | 0 | 1 | 0 | 0 | 1 | 1 | 0 |
| New.CleanUp.ReferenceOTU72307 | 0 | 1 | 0 | 0 | 1 | 1 | 0 |
| New.CleanUp.ReferenceOTU72786 | 1 | 1 | 0 | 1 | 1 | 1 | 0 |
| New.CleanUp.ReferenceOTU74874 | 0 | 1 | 0 | 0 | 1 | 1 | 0 |
| New.CleanUp.ReferenceOTU76729 | 0 | 0 | 0 | 1 | 0 | 0 | 0 |
| New.CleanUp.ReferenceOTU78498 | 0 | 1 | 0 | 0 | 1 | 0 | 0 |
| New.CleanUp.ReferenceOTU78527 | 0 | 1 | 0 | 1 | 1 | 1 | 0 |
| New.CleanUp.ReferenceOTU78920 | 0 | 0 | 0 | 0 | 1 | 1 | 0 |
| New.CleanUp.ReferenceOTU81275 | 0 | 1 | 0 | 0 | 0 | 1 | 0 |
| KJ762871.1.1791 | 1 | 1 | 1 | 1 | 1 | 1 | 0 |
| KJ759562.1.1795 | 1 | 1 | 1 | 1 | 1 | 1 | 0 |
| KJ758000.1.1805 | 1 | 0 | 1 | 1 | 1 | 0 | 0 |
| FJ000198.1.1388 | 0 | 1 | 1 | 1 | 1 | 1 | 0 |
| KJ757963.1.1801 | 1 | 1 | 1 | 1 | 1 | 1 | 1 |
| FJ914446.1.1527 | 1 | 1 | 1 | 1 | 1 | 1 | 0 |
| GU820700.1.1238 | 0 | 0 | 0 | 0 | 1 | 1 | 0 |
| KJ762958.1.1792 | 0 | 1 | 0 | 1 | 0 | 0 | 0 |
| KJ757837.1.1795 | 0 | 0 | 0 | 1 | 1 | 0 | 0 |
| New.ReferenceOTU63 | 1 | 1 | 0 | 0 | 1 | 1 | 0 |
| JN873310.1.1682 | 0 | 0 | 0 | 0 | 0 | 1 | 0 |
| New.CleanUp.ReferenceOTU34084 | 0 | 0 | 0 | 0 | 0 | 1 | 0 |
| KJ759689.1.1806 | 0 | 1 | 1 | 1 | 0 | 0 | 0 |
| New.CleanUp.ReferenceOTU7457 | 0 | 1 | 0 | 0 | 0 | 0 | 0 |
| New.CleanUp.ReferenceOTU8184 | 0 | 1 | 1 | 0 | 0 | 0 | 0 |
| New.CleanUp.ReferenceOTU8292 | 0 | 1 | 0 | 0 | 0 | 0 | 0 |
| New.CleanUp.ReferenceOTU23276 | 1 | 1 | 1 | 1 | 0 | 0 | 0 |
| New.CleanUp.ReferenceOTU27721 | 0 | 1 | 0 | 0 | 0 | 0 | 0 |
| New.CleanUp.ReferenceOTU41705 | 0 | 1 | 1 | 0 | 0 | 0 | 0 |
| New.CleanUp.ReferenceOTU49211 | 0 | 1 | 0 | 1 | 0 | 0 | 0 |
| New.CleanUp.ReferenceOTU51698 | 0 | 1 | 0 | 0 | 0 | 0 | 0 |
| New.CleanUp.ReferenceOTU68268 | 1 | 1 | 1 | 1 | 0 | 0 | 0 |
| New.CleanUp.ReferenceOTU71336 | 0 | 1 | 0 | 0 | 0 | 0 | 0 |
| New.CleanUp.ReferenceOTU71934 | 0 | 1 | 0 | 0 | 0 | 0 | 0 |
| New.CleanUp.ReferenceOTU75655 | 0 | 1 | 0 | 0 | 0 | 0 | 0 |
| New.CleanUp.ReferenceOTU78713 | 1 | 1 | 1 | 0 | 0 | 0 | 0 |
| AF231803.1.1797 | 1 | 1 | 1 | 1 | 0 | 0 | 0 |
| New.CleanUp.ReferenceOTU44719 | 0 | 0 | 0 | 0 | 0 | 1 | 0 |
| New.CleanUp.ReferenceOTU50642 | 0 | 0 | 0 | 1 | 0 | 0 | 0 |
| U27498.1.1798 | 0 | 0 | 0 | 1 | 0 | 0 | 0 |
| AB088298.1.1804 | 0 | 0 | 0 | 1 | 0 | 0 | 0 |
| New.CleanUp.ReferenceOTU61758 | 0 | 0 | 0 | 1 | 0 | 0 | 0 |
| New.CleanUp.ReferenceOTU78345 | 0 | 0 | 0 | 1 | 0 | 0 | 0 |
| New.CleanUp.ReferenceOTU2853 | 0 | 0 | 0 | 1 | 0 | 0 | 0 |
| AF330215.1.1759 | 1 | 1 | 1 | 1 | 1 | 1 | 0 |
| New.ReferenceOTU243 | 1 | 1 | 1 | 1 | 1 | 1 | 0 |
| New.CleanUp.ReferenceOTU4436 | 0 | 1 | 0 | 0 | 0 | 0 | 0 |
| New.CleanUp.ReferenceOTU22984 | 0 | 0 | 1 | 0 | 0 | 0 | 0 |
| New.CleanUp.ReferenceOTU25692 | 0 | 1 | 0 | 0 | 0 | 0 | 0 |
| New.CleanUp.ReferenceOTU79895 | 0 | 0 | 1 | 0 | 0 | 0 | 0 |
| New.CleanUp.ReferenceOTU66283 | 0 | 1 | 0 | 0 | 1 | 1 | 0 |
| New.CleanUp.ReferenceOTU51941 | 1 | 0 | 0 | 0 | 0 | 0 | 0 |
| EF527175.1.1544 | 1 | 1 | 1 | 1 | 1 | 0 | 1 |
| New.CleanUp.ReferenceOTU20561 | 0 | 1 | 1 | 1 | 0 | 0 | 0 |
| New.CleanUp.ReferenceOTU21805 | 0 | 1 | 0 | 0 | 0 | 0 | 0 |
| New.CleanUp.ReferenceOTU34787 | 0 | 1 | 0 | 0 | 0 | 0 | 0 |
| New.CleanUp.ReferenceOTU46934 | 1 | 1 | 1 | 1 | 1 | 0 | 1 |
| New.CleanUp.ReferenceOTU51175 | 0 | 0 | 1 | 0 | 0 | 0 | 0 |
| New.CleanUp.ReferenceOTU79088 | 0 | 0 | 1 | 0 | 0 | 0 | 0 |
| New.CleanUp.ReferenceOTU79215 | 0 | 1 | 0 | 0 | 0 | 0 | 0 |
| New.CleanUp.ReferenceOTU80091 | 0 | 1 | 0 | 0 | 0 | 0 | 0 |
| New.CleanUp.ReferenceOTU80321 | 0 | 0 | 0 | 0 | 0 | 0 | 1 |
| New.CleanUp.ReferenceOTU81093 | 0 | 1 | 0 | 1 | 0 | 0 | 0 |
| GU820015.1.1200 | 0 | 1 | 0 | 0 | 0 | 1 | 0 |
| New.CleanUp.ReferenceOTU834 | 0 | 1 | 0 | 0 | 0 | 1 | 0 |
| New.CleanUp.ReferenceOTU4022 | 0 | 1 | 0 | 0 | 0 | 0 | 0 |
| New.CleanUp.ReferenceOTU16497 | 0 | 1 | 1 | 0 | 0 | 0 | 0 |
| New.CleanUp.ReferenceOTU20038 | 0 | 1 | 0 | 0 | 0 | 0 | 0 |
| New.CleanUp.ReferenceOTU26946 | 0 | 0 | 0 | 1 | 0 | 0 | 0 |
| New.CleanUp.ReferenceOTU35010 | 0 | 1 | 0 | 0 | 0 | 0 | 0 |
| New.CleanUp.ReferenceOTU37013 | 0 | 1 | 1 | 0 | 0 | 0 | 0 |
| New.CleanUp.ReferenceOTU38264 | 0 | 1 | 0 | 0 | 0 | 0 | 0 |
| New.CleanUp.ReferenceOTU45037 | 0 | 1 | 0 | 0 | 0 | 0 | 0 |
| New.CleanUp.ReferenceOTU46523 | 0 | 0 | 0 | 0 | 0 | 0 | 1 |
| New.CleanUp.ReferenceOTU51537 | 0 | 1 | 0 | 0 | 0 | 0 | 0 |
| New.CleanUp.ReferenceOTU68645 | 0 | 1 | 0 | 0 | 0 | 0 | 0 |
| New.CleanUp.ReferenceOTU74327 | 0 | 1 | 0 | 1 | 0 | 0 | 0 |
| New.CleanUp.ReferenceOTU15594 | 0 | 0 | 1 | 0 | 0 | 0 | 0 |
| New.CleanUp.ReferenceOTU17647 | 0 | 0 | 0 | 0 | 0 | 0 | 1 |
| New.CleanUp.ReferenceOTU40582 | 0 | 1 | 0 | 0 | 0 | 0 | 0 |
| New.CleanUp.ReferenceOTU63228 | 0 | 1 | 0 | 0 | 0 | 0 | 0 |
| New.CleanUp.ReferenceOTU64165 | 0 | 0 | 1 | 0 | 0 | 0 | 0 |
| New.CleanUp.ReferenceOTU69657 | 0 | 1 | 0 | 0 | 0 | 0 | 0 |
| New.CleanUp.ReferenceOTU76614 | 0 | 1 | 0 | 0 | 0 | 0 | 0 |
| New.CleanUp.ReferenceOTU18288 | 0 | 0 | 0 | 0 | 0 | 0 | 1 |
| New.CleanUp.ReferenceOTU68233 | 0 | 1 | 0 | 0 | 0 | 0 | 0 |
| New.CleanUp.ReferenceOTU73923 | 0 | 1 | 0 | 0 | 0 | 0 | 0 |
| New.CleanUp.ReferenceOTU5809 | 0 | 0 | 0 | 0 | 0 | 0 | 1 |
| New.CleanUp.ReferenceOTU46739 | 0 | 0 | 0 | 0 | 0 | 1 | 1 |
| New.CleanUp.ReferenceOTU47005 | 0 | 0 | 0 | 0 | 0 | 1 | 1 |
| New.CleanUp.ReferenceOTU49251 | 0 | 0 | 0 | 0 | 0 | 1 | 1 |
| New.CleanUp.ReferenceOTU2256 | 0 | 0 | 0 | 1 | 0 | 0 | 1 |
| New.CleanUp.ReferenceOTU4382 | 0 | 0 | 0 | 0 | 0 | 0 | 1 |
| New.CleanUp.ReferenceOTU7504 | 1 | 1 | 1 | 0 | 0 | 1 | 0 |
| New.CleanUp.ReferenceOTU9144 | 0 | 1 | 0 | 1 | 0 | 0 | 0 |
| New.CleanUp.ReferenceOTU18424 | 1 | 1 | 0 | 1 | 0 | 1 | 0 |
| New.CleanUp.ReferenceOTU25298 | 0 | 0 | 0 | 1 | 0 | 0 | 1 |
| New.CleanUp.ReferenceOTU62388 | 1 | 1 | 0 | 1 | 0 | 1 | 0 |
| New.CleanUp.ReferenceOTU66100 | 1 | 1 | 1 | 0 | 0 | 1 | 0 |
| New.CleanUp.ReferenceOTU71696 | 1 | 0 | 0 | 0 | 0 | 1 | 0 |
| New.CleanUp.ReferenceOTU40060 | 0 | 1 | 0 | 0 | 0 | 0 | 0 |
| AY180045.1.1720 | 0 | 1 | 0 | 0 | 0 | 0 | 0 |
| New.CleanUp.ReferenceOTU78754 | 0 | 1 | 0 | 0 | 0 | 0 | 0 |
| New.CleanUp.ReferenceOTU66960 | 0 | 1 | 0 | 0 | 0 | 0 | 0 |
| New.CleanUp.ReferenceOTU54838 | 0 | 0 | 1 | 0 | 0 | 0 | 0 |
| EF526884.1.1802 | 0 | 1 | 0 | 0 | 0 | 1 | 0 |
| New.CleanUp.ReferenceOTU7346 | 0 | 1 | 0 | 0 | 0 | 1 | 0 |
| New.CleanUp.ReferenceOTU13577 | 0 | 0 | 0 | 1 | 0 | 1 | 1 |
| New.CleanUp.ReferenceOTU75405 | 0 | 0 | 0 | 1 | 0 | 1 | 0 |
| JX457422.1.1496 | 1 | 1 | 0 | 0 | 1 | 1 | 1 |
| KJ762900.1.1797 | 0 | 0 | 0 | 0 | 0 | 1 | 0 |
| JF826354.1.1611 | 0 | 1 | 1 | 1 | 0 | 0 | 0 |
| KJ759247.1.1802 | 1 | 1 | 1 | 1 | 1 | 1 | 1 |
| New.ReferenceOTU329 | 1 | 1 | 1 | 1 | 1 | 1 | 1 |
| New.ReferenceOTU337 | 1 | 1 | 1 | 1 | 1 | 1 | 1 |
| New.ReferenceOTU146 | 1 | 1 | 1 | 1 | 1 | 1 | 1 |
| New.CleanUp.ReferenceOTU13721 | 0 | 0 | 0 | 0 | 0 | 1 | 0 |
| New.CleanUp.ReferenceOTU16427 | 0 | 0 | 0 | 0 | 1 | 1 | 0 |
| New.CleanUp.ReferenceOTU18655 | 0 | 0 | 0 | 0 | 1 | 0 | 0 |
| New.CleanUp.ReferenceOTU19551 | 0 | 0 | 0 | 0 | 1 | 1 | 0 |
| New.CleanUp.ReferenceOTU20394 | 0 | 1 | 1 | 1 | 0 | 0 | 0 |
| New.CleanUp.ReferenceOTU25947 | 0 | 1 | 0 | 0 | 1 | 1 | 0 |
| New.CleanUp.ReferenceOTU28432 | 0 | 1 | 1 | 1 | 0 | 0 | 0 |
| New.CleanUp.ReferenceOTU28498 | 0 | 0 | 0 | 0 | 0 | 1 | 0 |
| New.CleanUp.ReferenceOTU46264 | 0 | 0 | 0 | 0 | 0 | 1 | 0 |
| New.CleanUp.ReferenceOTU58706 | 0 | 0 | 1 | 0 | 1 | 1 | 0 |
| New.CleanUp.ReferenceOTU65829 | 1 | 1 | 1 | 1 | 0 | 1 | 0 |
| KJ763267.1.1797 | 1 | 1 | 1 | 1 | 1 | 1 | 1 |
| JN098199.1.1537 | 1 | 0 | 1 | 0 | 0 | 0 | 0 |
| New.CleanUp.ReferenceOTU14721 | 0 | 1 | 0 | 0 | 1 | 1 | 0 |
| New.CleanUp.ReferenceOTU71748 | 0 | 0 | 0 | 0 | 0 | 1 | 0 |
| New.CleanUp.ReferenceOTU25399 | 0 | 0 | 0 | 0 | 0 | 1 | 1 |
| New.CleanUp.ReferenceOTU62550 | 0 | 0 | 1 | 0 | 0 | 0 | 0 |
| GBRZ01000219.49.1839 | 0 | 0 | 0 | 0 | 0 | 1 | 0 |
| EU190867.1.1817 | 0 | 1 | 0 | 0 | 0 | 1 | 0 |
| New.CleanUp.ReferenceOTU16857 | 0 | 1 | 1 | 1 | 1 | 1 | 1 |
| New.CleanUp.ReferenceOTU35332 | 0 | 0 | 0 | 1 | 1 | 1 | 1 |
| New.CleanUp.ReferenceOTU6911 | 0 | 1 | 0 | 1 | 0 | 0 | 0 |
| New.CleanUp.ReferenceOTU29305 | 1 | 1 | 0 | 1 | 1 | 1 | 0 |
| New.ReferenceOTU258 | 1 | 0 | 1 | 0 | 1 | 1 | 0 |
| New.CleanUp.ReferenceOTU12894 | 1 | 0 | 1 | 1 | 1 | 0 | 0 |
| New.CleanUp.ReferenceOTU22366 | 1 | 0 | 0 | 0 | 0 | 0 | 0 |
| New.CleanUp.ReferenceOTU24988 | 1 | 0 | 0 | 0 | 0 | 0 | 0 |
| New.CleanUp.ReferenceOTU26663 | 0 | 1 | 0 | 1 | 1 | 1 | 0 |
| New.CleanUp.ReferenceOTU27857 | 0 | 1 | 1 | 1 | 0 | 0 | 0 |
| New.CleanUp.ReferenceOTU49490 | 0 | 1 | 1 | 1 | 0 | 1 | 0 |
| New.CleanUp.ReferenceOTU69064 | 0 | 0 | 1 | 1 | 0 | 1 | 0 |
| New.CleanUp.ReferenceOTU71255 | 0 | 0 | 1 | 1 | 0 | 1 | 0 |
| New.CleanUp.ReferenceOTU5989 | 0 | 1 | 0 | 0 | 0 | 0 | 0 |
| New.CleanUp.ReferenceOTU7596 | 0 | 1 | 0 | 0 | 0 | 0 | 0 |
| New.CleanUp.ReferenceOTU8074 | 0 | 1 | 0 | 0 | 0 | 0 | 0 |
| New.CleanUp.ReferenceOTU9283 | 1 | 1 | 0 | 0 | 1 | 1 | 0 |
| New.CleanUp.ReferenceOTU23614 | 1 | 0 | 1 | 0 | 0 | 0 | 0 |
| New.CleanUp.ReferenceOTU40656 | 1 | 1 | 0 | 0 | 1 | 1 | 0 |
| New.CleanUp.ReferenceOTU40746 | 0 | 1 | 1 | 0 | 0 | 0 | 0 |
| New.CleanUp.ReferenceOTU46294 | 0 | 1 | 0 | 0 | 0 | 0 | 0 |
| New.CleanUp.ReferenceOTU51503 | 0 | 1 | 0 | 0 | 0 | 0 | 0 |
| New.CleanUp.ReferenceOTU54542 | 0 | 1 | 0 | 0 | 0 | 0 | 0 |
| New.CleanUp.ReferenceOTU55792 | 0 | 1 | 0 | 0 | 0 | 0 | 0 |
| New.CleanUp.ReferenceOTU57749 | 0 | 1 | 1 | 0 | 0 | 0 | 0 |
| New.CleanUp.ReferenceOTU66242 | 1 | 1 | 0 | 0 | 0 | 0 | 0 |
| New.CleanUp.ReferenceOTU72689 | 1 | 0 | 1 | 0 | 0 | 0 | 0 |
| New.CleanUp.ReferenceOTU17693 | 0 | 1 | 0 | 0 | 0 | 0 | 0 |
| New.CleanUp.ReferenceOTU56970 | 0 | 0 | 0 | 0 | 0 | 1 | 0 |
| New.CleanUp.ReferenceOTU59321 | 0 | 1 | 1 | 0 | 1 | 1 | 0 |
| New.ReferenceOTU136 | 0 | 1 | 1 | 1 | 1 | 0 | 0 |
| New.CleanUp.ReferenceOTU5138 | 0 | 1 | 0 | 1 | 0 | 0 | 0 |
| New.CleanUp.ReferenceOTU9556 | 0 | 0 | 0 | 1 | 0 | 0 | 0 |
| New.CleanUp.ReferenceOTU10203 | 0 | 1 | 0 | 0 | 0 | 0 | 0 |
| New.CleanUp.ReferenceOTU12679 | 0 | 0 | 0 | 0 | 0 | 1 | 0 |
| New.CleanUp.ReferenceOTU43727 | 0 | 1 | 1 | 1 | 1 | 0 | 0 |
| New.CleanUp.ReferenceOTU59310 | 0 | 1 | 0 | 1 | 0 | 0 | 0 |
| New.CleanUp.ReferenceOTU60210 | 0 | 1 | 0 | 0 | 0 | 0 | 0 |
| New.CleanUp.ReferenceOTU62712 | 0 | 1 | 0 | 1 | 0 | 0 | 0 |
| New.CleanUp.ReferenceOTU63273 | 0 | 1 | 0 | 1 | 0 | 0 | 0 |
| New.CleanUp.ReferenceOTU76589 | 0 | 1 | 0 | 1 | 0 | 0 | 0 |
| New.CleanUp.ReferenceOTU18272 | 0 | 1 | 0 | 0 | 1 | 0 | 0 |
| New.CleanUp.ReferenceOTU1161 | 0 | 0 | 1 | 0 | 0 | 0 | 1 |
| New.CleanUp.ReferenceOTU1241 | 0 | 1 | 0 | 1 | 1 | 1 | 0 |
| New.CleanUp.ReferenceOTU2464 | 0 | 0 | 0 | 0 | 1 | 1 | 1 |
| New.CleanUp.ReferenceOTU5097 | 0 | 0 | 0 | 0 | 1 | 0 | 0 |
| New.CleanUp.ReferenceOTU8111 | 0 | 1 | 0 | 1 | 1 | 1 | 0 |
| New.CleanUp.ReferenceOTU11648 | 0 | 0 | 1 | 0 | 1 | 1 | 0 |
| New.CleanUp.ReferenceOTU11683 | 0 | 0 | 0 | 0 | 1 | 0 | 1 |
| New.CleanUp.ReferenceOTU11990 | 0 | 0 | 0 | 0 | 1 | 0 | 0 |
| New.CleanUp.ReferenceOTU13823 | 0 | 1 | 0 | 1 | 1 | 1 | 0 |
| New.CleanUp.ReferenceOTU14013 | 0 | 1 | 0 | 1 | 0 | 1 | 0 |
| New.CleanUp.ReferenceOTU17558 | 0 | 1 | 0 | 1 | 1 | 1 | 0 |
| New.CleanUp.ReferenceOTU17926 | 0 | 1 | 0 | 0 | 0 | 1 | 0 |
| New.CleanUp.ReferenceOTU18653 | 0 | 0 | 0 | 1 | 0 | 1 | 1 |
| New.CleanUp.ReferenceOTU20864 | 0 | 1 | 1 | 1 | 0 | 0 | 0 |
| New.CleanUp.ReferenceOTU21067 | 0 | 1 | 0 | 1 | 0 | 1 | 1 |
| New.CleanUp.ReferenceOTU21958 | 0 | 1 | 0 | 0 | 0 | 1 | 1 |
| New.CleanUp.ReferenceOTU21970 | 0 | 0 | 1 | 0 | 0 | 1 | 0 |
| New.CleanUp.ReferenceOTU25226 | 0 | 1 | 0 | 0 | 0 | 0 | 0 |
| New.CleanUp.ReferenceOTU28394 | 0 | 1 | 0 | 0 | 0 | 1 | 0 |
| New.CleanUp.ReferenceOTU29028 | 0 | 0 | 0 | 1 | 0 | 1 | 0 |
| New.CleanUp.ReferenceOTU30054 | 0 | 0 | 0 | 0 | 0 | 1 | 1 |
| New.CleanUp.ReferenceOTU32406 | 0 | 1 | 0 | 0 | 0 | 0 | 0 |
| New.CleanUp.ReferenceOTU33839 | 0 | 1 | 0 | 1 | 0 | 1 | 0 |
| New.CleanUp.ReferenceOTU37214 | 0 | 0 | 0 | 1 | 1 | 0 | 1 |
| New.CleanUp.ReferenceOTU39673 | 0 | 1 | 0 | 0 | 0 | 1 | 0 |
| New.CleanUp.ReferenceOTU45522 | 0 | 1 | 0 | 0 | 0 | 0 | 1 |
| New.CleanUp.ReferenceOTU47171 | 0 | 0 | 0 | 1 | 0 | 0 | 1 |
| New.CleanUp.ReferenceOTU48738 | 0 | 1 | 0 | 1 | 1 | 1 | 1 |
| New.CleanUp.ReferenceOTU52315 | 0 | 0 | 0 | 1 | 0 | 0 | 0 |
| New.CleanUp.ReferenceOTU53848 | 0 | 0 | 0 | 0 | 0 | 0 | 1 |
| New.CleanUp.ReferenceOTU55688 | 0 | 0 | 1 | 1 | 0 | 0 | 0 |
| New.CleanUp.ReferenceOTU55873 | 0 | 1 | 0 | 1 | 1 | 1 | 0 |
| New.CleanUp.ReferenceOTU57191 | 0 | 1 | 0 | 0 | 1 | 1 | 1 |
| New.CleanUp.ReferenceOTU59248 | 0 | 1 | 0 | 1 | 0 | 1 | 1 |
| New.CleanUp.ReferenceOTU59758 | 0 | 0 | 0 | 0 | 0 | 0 | 1 |
| New.CleanUp.ReferenceOTU63361 | 0 | 1 | 1 | 1 | 0 | 1 | 1 |
| New.CleanUp.ReferenceOTU64634 | 0 | 1 | 0 | 0 | 0 | 1 | 0 |
| New.CleanUp.ReferenceOTU65353 | 0 | 0 | 0 | 1 | 0 | 0 | 0 |
| New.CleanUp.ReferenceOTU66484 | 0 | 1 | 0 | 1 | 0 | 0 | 1 |
| New.CleanUp.ReferenceOTU66662 | 0 | 1 | 0 | 0 | 0 | 0 | 0 |
| New.CleanUp.ReferenceOTU66949 | 0 | 1 | 0 | 1 | 0 | 0 | 0 |
| New.CleanUp.ReferenceOTU67298 | 0 | 1 | 0 | 1 | 0 | 1 | 0 |
| New.CleanUp.ReferenceOTU67818 | 0 | 1 | 0 | 1 | 0 | 0 | 0 |
| New.CleanUp.ReferenceOTU69158 | 0 | 0 | 0 | 1 | 1 | 1 | 1 |
| New.CleanUp.ReferenceOTU69651 | 0 | 0 | 0 | 1 | 1 | 1 | 1 |
| New.CleanUp.ReferenceOTU69738 | 0 | 1 | 1 | 0 | 0 | 1 | 1 |
| New.CleanUp.ReferenceOTU71446 | 0 | 0 | 0 | 1 | 1 | 0 | 0 |
| New.CleanUp.ReferenceOTU73013 | 0 | 1 | 1 | 1 | 1 | 1 | 1 |
| New.CleanUp.ReferenceOTU74899 | 0 | 0 | 0 | 0 | 1 | 0 | 0 |
| New.CleanUp.ReferenceOTU76810 | 0 | 1 | 0 | 0 | 0 | 1 | 1 |
| New.CleanUp.ReferenceOTU77931 | 0 | 0 | 0 | 1 | 0 | 1 | 1 |
| New.CleanUp.ReferenceOTU79074 | 0 | 1 | 0 | 0 | 0 | 0 | 0 |
| DQ386748.1.1523 | 0 | 1 | 1 | 1 | 1 | 1 | 0 |
| KU743847.1.1799 | 0 | 1 | 0 | 1 | 1 | 1 | 0 |
| New.ReferenceOTU49 | 0 | 1 | 1 | 1 | 1 | 1 | 0 |
| New.CleanUp.ReferenceOTU19521 | 0 | 0 | 1 | 1 | 1 | 1 | 0 |
| New.CleanUp.ReferenceOTU28610 | 0 | 0 | 0 | 0 | 1 | 0 | 0 |
| New.CleanUp.ReferenceOTU59460 | 0 | 0 | 0 | 1 | 0 | 0 | 0 |
| New.CleanUp.ReferenceOTU59757 | 0 | 0 | 0 | 1 | 1 | 0 | 0 |
| New.ReferenceOTU213 | 1 | 1 | 1 | 1 | 1 | 1 | 1 |
| New.ReferenceOTU141 | 1 | 1 | 1 | 1 | 1 | 1 | 1 |
| New.CleanUp.ReferenceOTU33564 | 0 | 0 | 0 | 1 | 0 | 0 | 0 |
| New.CleanUp.ReferenceOTU44077 | 1 | 0 | 0 | 0 | 0 | 1 | 0 |
| KJ757867.1.1810 | 0 | 0 | 0 | 1 | 1 | 0 | 0 |
| New.ReferenceOTU80 | 0 | 0 | 0 | 0 | 1 | 0 | 0 |
| New.CleanUp.ReferenceOTU1881 | 0 | 0 | 0 | 0 | 1 | 0 | 0 |
| New.CleanUp.ReferenceOTU9137 | 0 | 1 | 0 | 1 | 0 | 1 | 0 |
| New.CleanUp.ReferenceOTU9992 | 0 | 0 | 1 | 0 | 1 | 0 | 0 |
| New.CleanUp.ReferenceOTU11846 | 0 | 0 | 0 | 0 | 0 | 1 | 0 |
| New.CleanUp.ReferenceOTU19006 | 0 | 1 | 0 | 0 | 0 | 1 | 0 |
| New.CleanUp.ReferenceOTU23813 | 0 | 0 | 0 | 0 | 0 | 1 | 0 |
| New.CleanUp.ReferenceOTU29411 | 0 | 0 | 0 | 1 | 1 | 0 | 0 |
| New.CleanUp.ReferenceOTU35381 | 0 | 0 | 0 | 1 | 1 | 0 | 0 |
| New.CleanUp.ReferenceOTU44704 | 0 | 0 | 0 | 0 | 0 | 0 | 1 |
| New.CleanUp.ReferenceOTU55660 | 0 | 1 | 0 | 1 | 1 | 0 | 0 |
| New.CleanUp.ReferenceOTU56050 | 0 | 1 | 0 | 1 | 0 | 1 | 0 |
| New.CleanUp.ReferenceOTU56517 | 0 | 0 | 0 | 0 | 1 | 0 | 0 |
| New.CleanUp.ReferenceOTU62039 | 0 | 0 | 0 | 0 | 0 | 0 | 1 |
| DQ504325.1.1684 | 0 | 0 | 0 | 0 | 1 | 0 | 0 |
| LC054930.1.1771 | 0 | 1 | 1 | 1 | 1 | 1 | 1 |
| KJ758221.1.1803 | 1 | 1 | 0 | 1 | 1 | 0 | 0 |
| KJ764223.1.1893 | 1 | 1 | 1 | 1 | 1 | 1 | 1 |
| KJ757356.1.1795 | 1 | 0 | 0 | 1 | 0 | 0 | 1 |
| KJ763635.1.1804 | 0 | 0 | 0 | 1 | 1 | 1 | 0 |
| KJ763891.1.1800 | 1 | 0 | 0 | 0 | 1 | 1 | 0 |
| GU819864.1.1219 | 0 | 0 | 1 | 1 | 1 | 1 | 0 |
| AB626150.1.1790 | 1 | 1 | 1 | 0 | 1 | 0 | 0 |
| KJ762844.1.1802 | 1 | 0 | 0 | 0 | 1 | 1 | 0 |
| KJ757157.1.1806 | 1 | 1 | 1 | 1 | 1 | 1 | 1 |
| FJ000199.1.1378 | 1 | 1 | 1 | 1 | 1 | 1 | 0 |
| AJ506972.1.1802 | 1 | 0 | 1 | 1 | 1 | 1 | 1 |
| AJ968729.1.1806 | 1 | 1 | 1 | 1 | 1 | 1 | 0 |
| JF791024.1.1777 | 1 | 1 | 1 | 0 | 1 | 1 | 0 |
| KJ758159.1.1804 | 1 | 1 | 0 | 1 | 0 | 0 | 0 |
| New.CleanUp.ReferenceOTU10695 | 1 | 0 | 0 | 0 | 0 | 0 | 0 |
| New.CleanUp.ReferenceOTU23157 | 1 | 1 | 0 | 1 | 1 | 1 | 1 |
| New.CleanUp.ReferenceOTU37519 | 0 | 0 | 1 | 0 | 0 | 0 | 0 |
| New.CleanUp.ReferenceOTU64766 | 0 | 0 | 0 | 1 | 0 | 0 | 0 |
| New.CleanUp.ReferenceOTU65833 | 1 | 0 | 0 | 0 | 0 | 0 | 0 |
| New.CleanUp.ReferenceOTU70650 | 1 | 1 | 1 | 0 | 0 | 0 | 0 |
| New.CleanUp.ReferenceOTU76243 | 0 | 0 | 0 | 1 | 1 | 0 | 0 |
| New.CleanUp.ReferenceOTU15852 | 0 | 1 | 0 | 1 | 0 | 0 | 0 |
| New.CleanUp.ReferenceOTU16617 | 0 | 1 | 0 | 0 | 0 | 0 | 0 |
| New.CleanUp.ReferenceOTU16706 | 0 | 0 | 0 | 1 | 0 | 0 | 0 |
| New.CleanUp.ReferenceOTU17313 | 0 | 0 | 0 | 0 | 0 | 1 | 0 |
| New.CleanUp.ReferenceOTU17913 | 0 | 1 | 0 | 0 | 0 | 1 | 0 |
| New.CleanUp.ReferenceOTU18352 | 1 | 0 | 0 | 0 | 0 | 0 | 0 |
| New.CleanUp.ReferenceOTU19945 | 0 | 1 | 0 | 0 | 0 | 0 | 0 |
| New.CleanUp.ReferenceOTU20499 | 0 | 0 | 0 | 0 | 1 | 1 | 0 |
| New.CleanUp.ReferenceOTU21020 | 0 | 1 | 0 | 0 | 1 | 0 | 0 |
| New.CleanUp.ReferenceOTU21221 | 0 | 0 | 1 | 0 | 1 | 1 | 0 |
| New.CleanUp.ReferenceOTU21691 | 0 | 1 | 1 | 1 | 1 | 1 | 1 |
| New.CleanUp.ReferenceOTU21821 | 0 | 1 | 0 | 0 | 0 | 0 | 0 |
| New.CleanUp.ReferenceOTU21878 | 0 | 1 | 0 | 1 | 1 | 0 | 0 |
| New.CleanUp.ReferenceOTU22388 | 0 | 1 | 0 | 0 | 0 | 0 | 0 |
| New.CleanUp.ReferenceOTU22578 | 0 | 0 | 0 | 0 | 0 | 1 | 0 |
| New.CleanUp.ReferenceOTU22716 | 0 | 0 | 0 | 0 | 1 | 1 | 0 |
| New.CleanUp.ReferenceOTU22989 | 1 | 1 | 1 | 1 | 1 | 1 | 1 |
| New.CleanUp.ReferenceOTU24301 | 0 | 1 | 0 | 0 | 0 | 0 | 0 |
| New.CleanUp.ReferenceOTU25221 | 0 | 1 | 0 | 0 | 0 | 0 | 0 |
| New.CleanUp.ReferenceOTU25275 | 1 | 1 | 1 | 1 | 1 | 1 | 0 |
| New.CleanUp.ReferenceOTU26236 | 0 | 0 | 0 | 1 | 1 | 1 | 1 |
| New.CleanUp.ReferenceOTU26360 | 0 | 1 | 0 | 0 | 0 | 0 | 0 |
| New.CleanUp.ReferenceOTU26584 | 0 | 0 | 0 | 0 | 1 | 1 | 0 |
| New.CleanUp.ReferenceOTU27012 | 0 | 0 | 0 | 1 | 0 | 0 | 0 |
| New.CleanUp.ReferenceOTU27471 | 1 | 0 | 0 | 0 | 0 | 0 | 0 |
| New.CleanUp.ReferenceOTU27475 | 1 | 0 | 1 | 0 | 0 | 0 | 0 |
| New.CleanUp.ReferenceOTU27508 | 0 | 0 | 0 | 0 | 1 | 0 | 0 |
| New.CleanUp.ReferenceOTU27656 | 0 | 1 | 0 | 0 | 0 | 0 | 0 |
| New.CleanUp.ReferenceOTU27906 | 0 | 1 | 1 | 0 | 0 | 1 | 0 |
| New.CleanUp.ReferenceOTU27998 | 0 | 1 | 1 | 1 | 1 | 1 | 0 |
| New.CleanUp.ReferenceOTU28264 | 0 | 1 | 1 | 1 | 0 | 0 | 0 |
| New.CleanUp.ReferenceOTU28342 | 0 | 1 | 0 | 1 | 0 | 1 | 0 |
| New.CleanUp.ReferenceOTU28398 | 0 | 0 | 0 | 1 | 0 | 0 | 0 |
| New.CleanUp.ReferenceOTU29157 | 1 | 0 | 0 | 1 | 0 | 1 | 0 |
| New.CleanUp.ReferenceOTU29173 | 0 | 0 | 0 | 1 | 1 | 1 | 0 |
| New.CleanUp.ReferenceOTU29406 | 0 | 1 | 1 | 0 | 1 | 0 | 0 |
| New.CleanUp.ReferenceOTU29734 | 0 | 0 | 0 | 0 | 1 | 0 | 0 |
| New.CleanUp.ReferenceOTU29929 | 0 | 0 | 1 | 0 | 0 | 0 | 0 |
| New.CleanUp.ReferenceOTU29960 | 1 | 1 | 0 | 1 | 0 | 1 | 0 |
| New.CleanUp.ReferenceOTU30675 | 0 | 0 | 0 | 0 | 1 | 1 | 0 |
| New.CleanUp.ReferenceOTU32206 | 0 | 1 | 0 | 0 | 1 | 0 | 0 |
| New.CleanUp.ReferenceOTU32271 | 0 | 1 | 0 | 0 | 0 | 0 | 0 |
| New.CleanUp.ReferenceOTU33019 | 0 | 0 | 0 | 0 | 1 | 0 | 0 |
| New.CleanUp.ReferenceOTU33198 | 0 | 0 | 0 | 1 | 0 | 1 | 0 |
| New.CleanUp.ReferenceOTU33245 | 0 | 1 | 0 | 0 | 0 | 0 | 0 |
| New.CleanUp.ReferenceOTU33256 | 0 | 1 | 0 | 0 | 1 | 0 | 0 |
| New.CleanUp.ReferenceOTU33570 | 0 | 0 | 1 | 0 | 0 | 0 | 0 |
| New.CleanUp.ReferenceOTU33662 | 0 | 1 | 0 | 0 | 0 | 0 | 0 |
| New.CleanUp.ReferenceOTU33852 | 1 | 0 | 0 | 0 | 0 | 0 | 0 |
| New.CleanUp.ReferenceOTU35062 | 1 | 1 | 0 | 0 | 0 | 0 | 0 |
| New.CleanUp.ReferenceOTU35711 | 0 | 0 | 0 | 0 | 1 | 0 | 0 |
| New.CleanUp.ReferenceOTU37777 | 0 | 0 | 0 | 0 | 1 | 1 | 0 |
| New.CleanUp.ReferenceOTU37824 | 0 | 1 | 0 | 0 | 0 | 0 | 0 |
| New.CleanUp.ReferenceOTU38102 | 0 | 0 | 0 | 0 | 1 | 0 | 0 |
| New.CleanUp.ReferenceOTU38223 | 0 | 1 | 0 | 1 | 1 | 0 | 0 |
| New.CleanUp.ReferenceOTU39000 | 1 | 0 | 0 | 0 | 0 | 0 | 0 |
| New.CleanUp.ReferenceOTU39551 | 1 | 0 | 0 | 1 | 0 | 1 | 0 |
| New.CleanUp.ReferenceOTU39792 | 1 | 0 | 1 | 0 | 0 | 0 | 0 |
| New.CleanUp.ReferenceOTU39996 | 0 | 1 | 0 | 1 | 0 | 1 | 0 |
| New.CleanUp.ReferenceOTU41405 | 0 | 0 | 0 | 0 | 1 | 0 | 0 |
| New.CleanUp.ReferenceOTU42252 | 1 | 0 | 0 | 1 | 0 | 0 | 1 |
| New.CleanUp.ReferenceOTU42396 | 0 | 0 | 0 | 1 | 0 | 1 | 0 |
| New.CleanUp.ReferenceOTU42904 | 1 | 1 | 0 | 0 | 0 | 0 | 0 |
| New.CleanUp.ReferenceOTU44054 | 0 | 1 | 0 | 1 | 0 | 0 | 0 |
| New.CleanUp.ReferenceOTU44087 | 0 | 0 | 0 | 0 | 1 | 0 | 0 |
| New.CleanUp.ReferenceOTU44373 | 0 | 1 | 0 | 0 | 1 | 0 | 0 |
| New.CleanUp.ReferenceOTU45059 | 0 | 1 | 1 | 1 | 0 | 1 | 0 |
| New.CleanUp.ReferenceOTU45100 | 1 | 0 | 1 | 0 | 0 | 0 | 0 |
| New.CleanUp.ReferenceOTU45275 | 0 | 0 | 0 | 0 | 1 | 1 | 0 |
| New.CleanUp.ReferenceOTU46086 | 0 | 1 | 0 | 1 | 0 | 0 | 0 |
| New.CleanUp.ReferenceOTU46766 | 0 | 0 | 0 | 0 | 1 | 0 | 0 |
| New.CleanUp.ReferenceOTU46957 | 0 | 0 | 0 | 0 | 0 | 1 | 0 |
| New.CleanUp.ReferenceOTU47440 | 1 | 0 | 0 | 0 | 0 | 0 | 0 |
| New.CleanUp.ReferenceOTU47518 | 0 | 1 | 0 | 0 | 1 | 0 | 0 |
| New.CleanUp.ReferenceOTU47536 | 0 | 1 | 0 | 1 | 1 | 0 | 0 |
| New.CleanUp.ReferenceOTU47550 | 0 | 1 | 0 | 0 | 1 | 0 | 0 |
| New.CleanUp.ReferenceOTU47734 | 0 | 1 | 1 | 1 | 1 | 0 | 0 |
| New.CleanUp.ReferenceOTU48124 | 0 | 1 | 0 | 1 | 0 | 1 | 0 |
| New.CleanUp.ReferenceOTU48234 | 0 | 1 | 0 | 0 | 1 | 0 | 0 |
| New.CleanUp.ReferenceOTU48311 | 1 | 1 | 0 | 0 | 1 | 0 | 0 |
| New.CleanUp.ReferenceOTU49259 | 0 | 0 | 0 | 1 | 0 | 0 | 0 |
| New.CleanUp.ReferenceOTU49820 | 0 | 1 | 0 | 0 | 0 | 0 | 0 |
| New.CleanUp.ReferenceOTU50126 | 0 | 0 | 0 | 0 | 0 | 1 | 0 |
| New.CleanUp.ReferenceOTU50862 | 0 | 0 | 0 | 1 | 1 | 0 | 0 |
| New.CleanUp.ReferenceOTU51560 | 0 | 1 | 0 | 0 | 0 | 0 | 0 |
| New.CleanUp.ReferenceOTU51872 | 0 | 1 | 0 | 1 | 0 | 0 | 0 |
| New.CleanUp.ReferenceOTU51965 | 0 | 0 | 1 | 0 | 1 | 0 | 0 |
| New.CleanUp.ReferenceOTU52014 | 0 | 1 | 0 | 1 | 0 | 1 | 0 |
| New.CleanUp.ReferenceOTU52302 | 1 | 1 | 1 | 0 | 1 | 1 | 0 |
| New.CleanUp.ReferenceOTU52661 | 0 | 1 | 1 | 0 | 0 | 0 | 0 |
| New.CleanUp.ReferenceOTU53859 | 0 | 0 | 1 | 0 | 0 | 0 | 0 |
| New.CleanUp.ReferenceOTU53907 | 0 | 0 | 0 | 1 | 0 | 0 | 0 |
| New.CleanUp.ReferenceOTU54282 | 0 | 0 | 0 | 0 | 0 | 1 | 0 |
| New.CleanUp.ReferenceOTU54652 | 1 | 1 | 0 | 1 | 0 | 0 | 0 |
| New.CleanUp.ReferenceOTU54662 | 0 | 1 | 0 | 0 | 0 | 1 | 0 |
| New.CleanUp.ReferenceOTU54696 | 0 | 0 | 0 | 0 | 1 | 0 | 0 |
| New.CleanUp.ReferenceOTU54742 | 0 | 0 | 0 | 0 | 1 | 0 | 0 |
| New.CleanUp.ReferenceOTU54810 | 0 | 0 | 0 | 1 | 0 | 1 | 0 |
| New.CleanUp.ReferenceOTU55906 | 0 | 0 | 0 | 0 | 1 | 0 | 0 |
| New.CleanUp.ReferenceOTU56222 | 0 | 1 | 0 | 1 | 0 | 1 | 0 |
| New.CleanUp.ReferenceOTU57660 | 1 | 0 | 0 | 0 | 0 | 0 | 0 |
| New.CleanUp.ReferenceOTU57868 | 0 | 1 | 0 | 0 | 1 | 0 | 0 |
| New.CleanUp.ReferenceOTU57871 | 1 | 1 | 0 | 1 | 1 | 1 | 1 |
| New.CleanUp.ReferenceOTU58172 | 0 | 0 | 0 | 1 | 1 | 0 | 0 |
| New.CleanUp.ReferenceOTU58727 | 0 | 0 | 0 | 1 | 0 | 0 | 0 |
| New.CleanUp.ReferenceOTU58996 | 0 | 1 | 0 | 0 | 1 | 1 | 0 |
| New.CleanUp.ReferenceOTU59640 | 0 | 1 | 0 | 0 | 0 | 1 | 0 |
| New.CleanUp.ReferenceOTU59670 | 1 | 1 | 1 | 0 | 0 | 0 | 0 |
| New.CleanUp.ReferenceOTU59732 | 1 | 0 | 0 | 0 | 0 | 0 | 0 |
| New.CleanUp.ReferenceOTU59771 | 0 | 0 | 0 | 1 | 1 | 1 | 0 |
| New.CleanUp.ReferenceOTU60155 | 1 | 1 | 0 | 1 | 1 | 1 | 0 |
| New.CleanUp.ReferenceOTU60380 | 0 | 1 | 0 | 0 | 1 | 1 | 0 |
| New.CleanUp.ReferenceOTU61128 | 0 | 1 | 1 | 1 | 0 | 1 | 1 |
| New.CleanUp.ReferenceOTU61340 | 0 | 1 | 0 | 1 | 1 | 1 | 0 |
| New.CleanUp.ReferenceOTU61370 | 1 | 0 | 0 | 0 | 0 | 0 | 0 |
| New.CleanUp.ReferenceOTU61484 | 1 | 0 | 1 | 0 | 0 | 0 | 0 |
| New.CleanUp.ReferenceOTU61856 | 0 | 0 | 0 | 0 | 0 | 0 | 1 |
| New.CleanUp.ReferenceOTU62544 | 0 | 0 | 1 | 0 | 0 | 0 | 0 |
| New.CleanUp.ReferenceOTU62892 | 1 | 0 | 0 | 0 | 0 | 0 | 0 |
| New.CleanUp.ReferenceOTU63195 | 0 | 0 | 0 | 1 | 0 | 0 | 0 |
| New.CleanUp.ReferenceOTU63484 | 0 | 0 | 0 | 0 | 1 | 1 | 0 |
| New.CleanUp.ReferenceOTU63633 | 0 | 1 | 1 | 1 | 0 | 0 | 0 |
| New.CleanUp.ReferenceOTU63740 | 0 | 1 | 0 | 0 | 0 | 0 | 0 |
| New.CleanUp.ReferenceOTU63790 | 0 | 0 | 0 | 0 | 0 | 1 | 0 |
| New.CleanUp.ReferenceOTU64148 | 0 | 0 | 1 | 0 | 0 | 1 | 0 |
| New.CleanUp.ReferenceOTU64205 | 0 | 0 | 0 | 0 | 0 | 1 | 0 |
| New.CleanUp.ReferenceOTU64264 | 0 | 0 | 0 | 0 | 1 | 0 | 0 |
| New.CleanUp.ReferenceOTU64461 | 0 | 0 | 0 | 0 | 0 | 1 | 0 |
| New.CleanUp.ReferenceOTU64950 | 0 | 0 | 0 | 0 | 1 | 0 | 0 |
| New.CleanUp.ReferenceOTU65001 | 0 | 1 | 0 | 1 | 1 | 0 | 0 |
| New.CleanUp.ReferenceOTU65294 | 1 | 0 | 0 | 0 | 0 | 0 | 0 |
| New.CleanUp.ReferenceOTU65804 | 1 | 1 | 0 | 1 | 1 | 1 | 0 |
| New.CleanUp.ReferenceOTU66357 | 0 | 0 | 0 | 0 | 1 | 0 | 0 |
| New.CleanUp.ReferenceOTU66383 | 0 | 0 | 0 | 1 | 0 | 1 | 0 |
| New.CleanUp.ReferenceOTU66444 | 0 | 1 | 0 | 0 | 0 | 0 | 0 |
| New.CleanUp.ReferenceOTU66782 | 0 | 1 | 0 | 1 | 0 | 0 | 0 |
| New.CleanUp.ReferenceOTU67287 | 0 | 1 | 0 | 1 | 1 | 1 | 0 |
| New.CleanUp.ReferenceOTU67310 | 0 | 0 | 0 | 0 | 1 | 0 | 0 |
| New.CleanUp.ReferenceOTU67321 | 0 | 0 | 1 | 0 | 0 | 0 | 0 |
| New.CleanUp.ReferenceOTU67377 | 0 | 1 | 0 | 0 | 0 | 0 | 0 |
| New.CleanUp.ReferenceOTU67452 | 0 | 1 | 1 | 0 | 0 | 0 | 0 |
| New.CleanUp.ReferenceOTU67606 | 0 | 1 | 0 | 0 | 1 | 0 | 0 |
| New.CleanUp.ReferenceOTU67689 | 0 | 1 | 0 | 0 | 1 | 1 | 0 |
| New.CleanUp.ReferenceOTU68004 | 0 | 0 | 0 | 0 | 1 | 0 | 0 |
| New.CleanUp.ReferenceOTU68229 | 0 | 1 | 0 | 0 | 1 | 0 | 0 |
| New.CleanUp.ReferenceOTU68914 | 0 | 1 | 0 | 1 | 0 | 1 | 0 |
| New.CleanUp.ReferenceOTU69205 | 0 | 0 | 1 | 0 | 0 | 0 | 0 |
| New.CleanUp.ReferenceOTU69584 | 0 | 1 | 0 | 0 | 0 | 1 | 0 |
| New.ReferenceOTU82 | 1 | 1 | 1 | 1 | 0 | 0 | 0 |
| New.ReferenceOTU88 | 0 | 1 | 1 | 1 | 0 | 1 | 1 |
| New.ReferenceOTU264 | 1 | 1 | 1 | 1 | 1 | 0 | 0 |
| New.CleanUp.ReferenceOTU408 | 0 | 0 | 0 | 0 | 1 | 0 | 0 |
| New.CleanUp.ReferenceOTU587 | 0 | 1 | 1 | 1 | 0 | 1 | 0 |
| New.CleanUp.ReferenceOTU1518 | 0 | 0 | 0 | 0 | 0 | 1 | 0 |
| New.CleanUp.ReferenceOTU1644 | 0 | 1 | 0 | 1 | 0 | 0 | 0 |
| New.CleanUp.ReferenceOTU1794 | 0 | 0 | 1 | 0 | 1 | 0 | 0 |
| New.CleanUp.ReferenceOTU2095 | 0 | 0 | 0 | 0 | 1 | 0 | 0 |
| New.CleanUp.ReferenceOTU2407 | 0 | 0 | 0 | 0 | 0 | 1 | 0 |
| New.CleanUp.ReferenceOTU3255 | 0 | 0 | 0 | 1 | 0 | 0 | 0 |
| New.CleanUp.ReferenceOTU5100 | 0 | 0 | 0 | 1 | 0 | 0 | 0 |
| New.CleanUp.ReferenceOTU5335 | 0 | 1 | 0 | 1 | 0 | 1 | 1 |
| New.CleanUp.ReferenceOTU5357 | 1 | 1 | 1 | 1 | 0 | 0 | 0 |
| New.CleanUp.ReferenceOTU5482 | 1 | 1 | 0 | 0 | 1 | 1 | 0 |
| New.CleanUp.ReferenceOTU6185 | 0 | 1 | 1 | 1 | 0 | 0 | 0 |
| New.CleanUp.ReferenceOTU6612 | 0 | 0 | 0 | 0 | 0 | 1 | 0 |
| New.CleanUp.ReferenceOTU7141 | 0 | 0 | 1 | 0 | 0 | 0 | 0 |
| New.CleanUp.ReferenceOTU7381 | 0 | 0 | 0 | 0 | 1 | 0 | 0 |
| New.CleanUp.ReferenceOTU7738 | 0 | 1 | 0 | 1 | 0 | 0 | 0 |
| New.CleanUp.ReferenceOTU7860 | 0 | 0 | 0 | 0 | 0 | 1 | 0 |
| New.CleanUp.ReferenceOTU7944 | 0 | 1 | 0 | 0 | 1 | 1 | 0 |
| New.CleanUp.ReferenceOTU8073 | 0 | 1 | 0 | 0 | 0 | 1 | 0 |
| New.CleanUp.ReferenceOTU8283 | 1 | 0 | 0 | 0 | 1 | 1 | 0 |
| New.CleanUp.ReferenceOTU8402 | 0 | 0 | 0 | 0 | 1 | 0 | 0 |
| New.CleanUp.ReferenceOTU8653 | 1 | 0 | 1 | 1 | 0 | 0 | 0 |
| New.CleanUp.ReferenceOTU9796 | 0 | 1 | 1 | 0 | 1 | 0 | 0 |
| New.CleanUp.ReferenceOTU10278 | 0 | 1 | 0 | 0 | 0 | 0 | 0 |
| New.CleanUp.ReferenceOTU10488 | 0 | 1 | 0 | 1 | 0 | 0 | 0 |
| New.CleanUp.ReferenceOTU10890 | 0 | 1 | 0 | 0 | 0 | 0 | 0 |
| New.CleanUp.ReferenceOTU12379 | 0 | 1 | 1 | 1 | 1 | 1 | 0 |
| New.CleanUp.ReferenceOTU12527 | 0 | 0 | 0 | 0 | 1 | 0 | 0 |
| New.CleanUp.ReferenceOTU13645 | 0 | 0 | 1 | 0 | 0 | 0 | 0 |
| New.CleanUp.ReferenceOTU13698 | 0 | 0 | 0 | 0 | 0 | 1 | 0 |
| New.CleanUp.ReferenceOTU14162 | 0 | 0 | 0 | 0 | 1 | 0 | 0 |
| New.CleanUp.ReferenceOTU14739 | 0 | 0 | 0 | 0 | 0 | 1 | 0 |
| New.CleanUp.ReferenceOTU14839 | 0 | 1 | 0 | 0 | 0 | 0 | 0 |
| New.CleanUp.ReferenceOTU14892 | 0 | 1 | 0 | 0 | 1 | 0 | 0 |
| New.CleanUp.ReferenceOTU14916 | 0 | 1 | 0 | 1 | 0 | 1 | 0 |
| New.CleanUp.ReferenceOTU15200 | 0 | 1 | 0 | 1 | 0 | 1 | 0 |
| JF826393.1.1617 | 1 | 1 | 0 | 1 | 1 | 1 | 0 |
| KJ757793.1.1806 | 0 | 0 | 0 | 1 | 1 | 1 | 0 |
| KJ757613.1.1810 | 1 | 1 | 1 | 1 | 1 | 1 | 1 |
| KJ759399.1.1798 | 1 | 1 | 0 | 1 | 0 | 0 | 0 |
| AM503929.1.1802 | 1 | 0 | 0 | 1 | 1 | 1 | 0 |
| KJ757191.1.1801 | 0 | 1 | 0 | 1 | 1 | 1 | 0 |
| KJ762862.1.1800 | 1 | 1 | 0 | 0 | 0 | 1 | 0 |
| KJ757193.1.1802 | 1 | 1 | 0 | 0 | 1 | 1 | 0 |
| KJ762841.1.1801 | 0 | 1 | 1 | 1 | 0 | 1 | 0 |
| JN098211.1.1537 | 0 | 0 | 1 | 1 | 1 | 1 | 0 |
| KJ925446.1.1502 | 0 | 0 | 0 | 1 | 1 | 1 | 0 |
| KT860951.1.1807 | 1 | 0 | 0 | 0 | 1 | 1 | 0 |
| AF022194.1.1803 | 0 | 1 | 0 | 1 | 1 | 1 | 0 |
| LC054949.1.1767 | 0 | 0 | 0 | 1 | 0 | 1 | 1 |
| AY590483.1.1802 | 1 | 1 | 0 | 0 | 0 | 0 | 0 |
| KJ758170.1.1812 | 1 | 1 | 1 | 1 | 1 | 1 | 1 |
| KJ757124.1.1800 | 1 | 0 | 0 | 1 | 1 | 0 | 0 |
| KJ757441.1.1801 | 1 | 1 | 0 | 0 | 0 | 1 | 0 |
| KJ759690.1.1806 | 1 | 0 | 1 | 0 | 0 | 0 | 1 |
| KF751923.1.1729 | 0 | 0 | 0 | 0 | 0 | 1 | 0 |
| GU820339.1.1229 | 0 | 1 | 0 | 0 | 1 | 1 | 0 |
| KJ761545.1.1802 | 1 | 0 | 1 | 1 | 0 | 1 | 0 |
| AY590484.1.1805 | 1 | 0 | 0 | 1 | 1 | 1 | 0 |
| KJ758260.1.1802 | 0 | 0 | 0 | 1 | 1 | 1 | 1 |
| New.ReferenceOTU44 | 1 | 0 | 0 | 0 | 0 | 0 | 0 |
| New.ReferenceOTU73 | 1 | 1 | 1 | 1 | 1 | 0 | 0 |
| New.ReferenceOTU302 | 0 | 0 | 1 | 0 | 0 | 0 | 0 |
| New.ReferenceOTU20 | 1 | 1 | 1 | 1 | 1 | 1 | 1 |
| New.ReferenceOTU276 | 0 | 0 | 1 | 0 | 0 | 0 | 0 |
| New.ReferenceOTU353 | 0 | 0 | 0 | 0 | 1 | 0 | 0 |
| New.CleanUp.ReferenceOTU70517 | 1 | 1 | 1 | 1 | 1 | 1 | 0 |
| New.CleanUp.ReferenceOTU70829 | 0 | 0 | 0 | 0 | 1 | 0 | 0 |
| New.CleanUp.ReferenceOTU71268 | 1 | 0 | 0 | 0 | 0 | 0 | 0 |
| New.CleanUp.ReferenceOTU71776 | 0 | 0 | 0 | 0 | 0 | 1 | 0 |
| New.CleanUp.ReferenceOTU71889 | 0 | 0 | 0 | 0 | 0 | 1 | 0 |
| New.CleanUp.ReferenceOTU72225 | 0 | 0 | 0 | 0 | 0 | 1 | 0 |
| New.CleanUp.ReferenceOTU72369 | 0 | 0 | 1 | 0 | 0 | 0 | 0 |
| New.CleanUp.ReferenceOTU72617 | 0 | 0 | 0 | 0 | 0 | 1 | 0 |
| New.CleanUp.ReferenceOTU72898 | 0 | 1 | 0 | 1 | 1 | 1 | 0 |
| New.CleanUp.ReferenceOTU73559 | 0 | 0 | 0 | 0 | 1 | 0 | 0 |
| New.CleanUp.ReferenceOTU73693 | 0 | 0 | 1 | 1 | 1 | 1 | 0 |
| New.CleanUp.ReferenceOTU74461 | 0 | 1 | 0 | 0 | 0 | 0 | 0 |
| New.CleanUp.ReferenceOTU74515 | 1 | 1 | 1 | 1 | 1 | 1 | 0 |
| New.CleanUp.ReferenceOTU75012 | 0 | 0 | 0 | 1 | 1 | 0 | 0 |
| New.CleanUp.ReferenceOTU75392 | 1 | 1 | 0 | 0 | 0 | 1 | 0 |
| New.CleanUp.ReferenceOTU75490 | 1 | 1 | 0 | 1 | 1 | 1 | 0 |
| New.CleanUp.ReferenceOTU75773 | 0 | 1 | 0 | 0 | 1 | 1 | 0 |
| New.CleanUp.ReferenceOTU75876 | 0 | 0 | 0 | 0 | 0 | 1 | 1 |
| New.CleanUp.ReferenceOTU76664 | 1 | 0 | 1 | 1 | 0 | 0 | 0 |
| New.CleanUp.ReferenceOTU76704 | 0 | 1 | 0 | 0 | 0 | 0 | 0 |
| New.CleanUp.ReferenceOTU76998 | 0 | 0 | 0 | 0 | 1 | 1 | 0 |
| New.CleanUp.ReferenceOTU77380 | 0 | 0 | 0 | 0 | 1 | 1 | 0 |
| New.CleanUp.ReferenceOTU77839 | 0 | 0 | 0 | 0 | 0 | 1 | 0 |
| New.CleanUp.ReferenceOTU78291 | 0 | 0 | 0 | 0 | 0 | 1 | 0 |
| New.CleanUp.ReferenceOTU78323 | 1 | 0 | 0 | 0 | 0 | 0 | 0 |
| New.CleanUp.ReferenceOTU78370 | 0 | 1 | 1 | 0 | 1 | 0 | 0 |
| New.CleanUp.ReferenceOTU78429 | 1 | 1 | 0 | 0 | 0 | 0 | 0 |
| New.CleanUp.ReferenceOTU78486 | 0 | 1 | 0 | 0 | 1 | 0 | 0 |
| New.CleanUp.ReferenceOTU79073 | 0 | 0 | 0 | 1 | 0 | 1 | 0 |
| New.CleanUp.ReferenceOTU79332 | 0 | 1 | 1 | 1 | 0 | 1 | 1 |
| New.CleanUp.ReferenceOTU79454 | 0 | 0 | 0 | 1 | 0 | 0 | 0 |
| New.CleanUp.ReferenceOTU79860 | 1 | 1 | 1 | 0 | 0 | 1 | 0 |
| New.CleanUp.ReferenceOTU81020 | 0 | 0 | 0 | 0 | 0 | 1 | 0 |
| New.CleanUp.ReferenceOTU81321 | 1 | 0 | 0 | 1 | 0 | 1 | 0 |
| New.ReferenceOTU23 | 1 | 1 | 1 | 1 | 0 | 1 | 0 |
| New.CleanUp.ReferenceOTU71476 | 1 | 0 | 0 | 0 | 0 | 0 | 0 |
| New.CleanUp.ReferenceOTU66833 | 0 | 1 | 0 | 0 | 0 | 1 | 0 |
| New.CleanUp.ReferenceOTU74077 | 0 | 0 | 0 | 1 | 0 | 1 | 0 |
| New.CleanUp.ReferenceOTU316 | 0 | 0 | 0 | 1 | 0 | 0 | 0 |
| New.CleanUp.ReferenceOTU2722 | 0 | 0 | 1 | 1 | 0 | 0 | 0 |
| New.CleanUp.ReferenceOTU16707 | 0 | 0 | 1 | 0 | 0 | 0 | 0 |
| New.CleanUp.ReferenceOTU16786 | 1 | 0 | 0 | 0 | 0 | 0 | 0 |
| New.CleanUp.ReferenceOTU21212 | 0 | 1 | 0 | 1 | 0 | 0 | 0 |
| New.CleanUp.ReferenceOTU28677 | 1 | 1 | 0 | 0 | 0 | 0 | 0 |
| New.CleanUp.ReferenceOTU36032 | 0 | 0 | 1 | 0 | 0 | 0 | 1 |
| New.CleanUp.ReferenceOTU42568 | 0 | 0 | 1 | 0 | 0 | 0 | 1 |
| New.CleanUp.ReferenceOTU46366 | 0 | 0 | 1 | 1 | 0 | 0 | 0 |
| New.CleanUp.ReferenceOTU49372 | 0 | 0 | 0 | 0 | 0 | 0 | 1 |
| New.CleanUp.ReferenceOTU54145 | 1 | 1 | 1 | 1 | 0 | 1 | 0 |
| New.CleanUp.ReferenceOTU56563 | 0 | 0 | 1 | 0 | 0 | 0 | 0 |
| New.CleanUp.ReferenceOTU60568 | 0 | 0 | 0 | 1 | 0 | 0 | 0 |
| New.CleanUp.ReferenceOTU62942 | 0 | 0 | 1 | 0 | 0 | 0 | 1 |
| New.CleanUp.ReferenceOTU64728 | 0 | 1 | 0 | 1 | 0 | 0 | 0 |
| New.CleanUp.ReferenceOTU66032 | 0 | 0 | 1 | 1 | 1 | 1 | 1 |
| New.CleanUp.ReferenceOTU69889 | 0 | 0 | 0 | 0 | 0 | 0 | 1 |
| KJ763500.1.1801 | 0 | 1 | 1 | 0 | 0 | 0 | 0 |
| KJ759613.1.1802 | 0 | 1 | 0 | 1 | 0 | 0 | 0 |
| KF129639.1.1754 | 0 | 1 | 0 | 0 | 1 | 0 | 0 |
| AY129050.1.1798 | 0 | 0 | 0 | 0 | 1 | 0 | 0 |
| KJ759669.1.1803 | 0 | 1 | 1 | 0 | 0 | 0 | 0 |
| KJ757457.1.1800 | 0 | 1 | 0 | 1 | 0 | 1 | 0 |
| DQ386756.1.1535 | 1 | 1 | 1 | 1 | 1 | 1 | 1 |
| KJ759509.1.1799 | 0 | 1 | 1 | 0 | 0 | 0 | 0 |
| New.ReferenceOTU78 | 0 | 1 | 1 | 0 | 0 | 0 | 0 |
| New.ReferenceOTU152 | 0 | 1 | 0 | 1 | 1 | 1 | 1 |
| New.CleanUp.ReferenceOTU266 | 0 | 1 | 1 | 1 | 0 | 1 | 0 |
| New.CleanUp.ReferenceOTU347 | 0 | 0 | 0 | 0 | 1 | 0 | 0 |
| New.CleanUp.ReferenceOTU879 | 0 | 0 | 0 | 0 | 0 | 0 | 1 |
| New.CleanUp.ReferenceOTU1281 | 0 | 1 | 1 | 0 | 0 | 0 | 0 |
| New.CleanUp.ReferenceOTU1998 | 0 | 1 | 1 | 1 | 1 | 1 | 1 |
| New.CleanUp.ReferenceOTU2099 | 0 | 1 | 0 | 0 | 1 | 0 | 0 |
| New.CleanUp.ReferenceOTU4270 | 0 | 0 | 0 | 0 | 0 | 0 | 1 |
| New.CleanUp.ReferenceOTU4521 | 0 | 1 | 1 | 1 | 1 | 1 | 1 |
| New.CleanUp.ReferenceOTU4783 | 0 | 1 | 0 | 1 | 0 | 1 | 1 |
| New.CleanUp.ReferenceOTU4866 | 0 | 1 | 1 | 1 | 1 | 1 | 1 |
| New.CleanUp.ReferenceOTU5617 | 0 | 1 | 0 | 0 | 1 | 0 | 1 |
| New.CleanUp.ReferenceOTU8397 | 0 | 1 | 0 | 1 | 0 | 0 | 0 |
| New.CleanUp.ReferenceOTU8774 | 0 | 0 | 1 | 0 | 0 | 0 | 0 |
| New.CleanUp.ReferenceOTU9008 | 0 | 0 | 0 | 1 | 1 | 0 | 0 |
| New.CleanUp.ReferenceOTU11568 | 0 | 1 | 0 | 0 | 1 | 0 | 0 |
| New.CleanUp.ReferenceOTU12751 | 0 | 0 | 0 | 0 | 0 | 1 | 1 |
| New.CleanUp.ReferenceOTU15317 | 0 | 0 | 1 | 0 | 0 | 1 | 1 |
| New.CleanUp.ReferenceOTU17970 | 0 | 1 | 1 | 0 | 1 | 0 | 0 |
| New.CleanUp.ReferenceOTU18014 | 0 | 0 | 0 | 0 | 1 | 0 | 0 |
| New.CleanUp.ReferenceOTU18209 | 0 | 1 | 0 | 1 | 0 | 0 | 0 |
| New.CleanUp.ReferenceOTU18919 | 0 | 0 | 1 | 1 | 1 | 1 | 0 |
| New.CleanUp.ReferenceOTU19815 | 0 | 1 | 0 | 1 | 0 | 1 | 0 |
| New.CleanUp.ReferenceOTU20885 | 0 | 1 | 0 | 0 | 1 | 0 | 1 |
| New.CleanUp.ReferenceOTU21581 | 0 | 1 | 0 | 1 | 1 | 1 | 1 |
| New.CleanUp.ReferenceOTU21689 | 0 | 1 | 1 | 1 | 1 | 1 | 0 |
| New.CleanUp.ReferenceOTU22861 | 0 | 1 | 0 | 1 | 0 | 1 | 0 |
| New.CleanUp.ReferenceOTU25432 | 0 | 1 | 0 | 0 | 0 | 1 | 0 |
| New.CleanUp.ReferenceOTU26413 | 0 | 1 | 1 | 1 | 1 | 1 | 1 |
| New.CleanUp.ReferenceOTU27177 | 0 | 1 | 0 | 0 | 1 | 0 | 0 |
| New.CleanUp.ReferenceOTU27242 | 0 | 0 | 0 | 0 | 0 | 1 | 0 |
| New.CleanUp.ReferenceOTU29663 | 0 | 0 | 0 | 0 | 1 | 1 | 0 |
| New.CleanUp.ReferenceOTU30185 | 0 | 1 | 0 | 1 | 1 | 0 | 0 |
| New.CleanUp.ReferenceOTU33504 | 0 | 1 | 0 | 1 | 1 | 1 | 1 |
| New.CleanUp.ReferenceOTU34710 | 0 | 0 | 1 | 1 | 0 | 0 | 0 |
| New.CleanUp.ReferenceOTU36215 | 0 | 0 | 0 | 1 | 1 | 0 | 0 |
| New.CleanUp.ReferenceOTU41936 | 0 | 1 | 0 | 1 | 1 | 1 | 0 |
| New.CleanUp.ReferenceOTU42536 | 0 | 1 | 0 | 1 | 1 | 1 | 1 |
| New.CleanUp.ReferenceOTU42969 | 0 | 1 | 0 | 1 | 0 | 0 | 0 |
| New.CleanUp.ReferenceOTU43036 | 0 | 1 | 0 | 0 | 0 | 1 | 0 |
| New.CleanUp.ReferenceOTU43216 | 0 | 1 | 0 | 1 | 0 | 1 | 0 |
| New.CleanUp.ReferenceOTU44344 | 0 | 0 | 0 | 0 | 0 | 0 | 1 |
| New.CleanUp.ReferenceOTU44635 | 0 | 1 | 1 | 0 | 0 | 1 | 1 |
| New.CleanUp.ReferenceOTU44855 | 0 | 1 | 0 | 0 | 0 | 0 | 0 |
| New.CleanUp.ReferenceOTU47416 | 0 | 1 | 0 | 1 | 0 | 0 | 0 |
| New.CleanUp.ReferenceOTU48683 | 0 | 0 | 0 | 0 | 1 | 0 | 0 |
| New.CleanUp.ReferenceOTU48996 | 0 | 1 | 0 | 0 | 1 | 1 | 0 |
| New.CleanUp.ReferenceOTU49633 | 0 | 0 | 0 | 1 | 1 | 0 | 0 |
| New.CleanUp.ReferenceOTU50926 | 0 | 1 | 1 | 1 | 1 | 1 | 1 |
| New.CleanUp.ReferenceOTU54118 | 0 | 1 | 1 | 1 | 1 | 1 | 1 |
| New.CleanUp.ReferenceOTU54525 | 0 | 1 | 0 | 1 | 0 | 1 | 0 |
| New.CleanUp.ReferenceOTU55556 | 0 | 1 | 0 | 1 | 0 | 0 | 0 |
| New.CleanUp.ReferenceOTU57395 | 0 | 1 | 1 | 0 | 0 | 0 | 1 |
| New.CleanUp.ReferenceOTU57455 | 0 | 0 | 0 | 1 | 1 | 0 | 0 |
| New.CleanUp.ReferenceOTU57966 | 0 | 1 | 0 | 1 | 0 | 0 | 0 |
| New.CleanUp.ReferenceOTU58210 | 0 | 0 | 0 | 1 | 1 | 0 | 0 |
| New.CleanUp.ReferenceOTU58416 | 0 | 0 | 0 | 0 | 1 | 1 | 1 |
| New.CleanUp.ReferenceOTU60200 | 0 | 0 | 0 | 0 | 0 | 1 | 1 |
| New.CleanUp.ReferenceOTU62760 | 0 | 1 | 0 | 1 | 0 | 1 | 1 |
| New.CleanUp.ReferenceOTU63248 | 0 | 1 | 0 | 1 | 0 | 1 | 0 |
| New.CleanUp.ReferenceOTU64316 | 0 | 1 | 0 | 1 | 1 | 1 | 1 |
| New.CleanUp.ReferenceOTU66591 | 0 | 1 | 1 | 1 | 0 | 0 | 0 |
| New.CleanUp.ReferenceOTU67994 | 0 | 1 | 0 | 1 | 0 | 0 | 1 |
| New.CleanUp.ReferenceOTU68890 | 0 | 1 | 0 | 1 | 1 | 1 | 0 |
| New.CleanUp.ReferenceOTU70430 | 0 | 1 | 0 | 0 | 0 | 0 | 0 |
| New.CleanUp.ReferenceOTU71980 | 0 | 0 | 0 | 1 | 0 | 1 | 1 |
| New.CleanUp.ReferenceOTU72631 | 0 | 0 | 1 | 0 | 0 | 1 | 0 |
| New.CleanUp.ReferenceOTU73082 | 0 | 1 | 1 | 0 | 1 | 1 | 0 |
| New.CleanUp.ReferenceOTU73135 | 0 | 1 | 0 | 0 | 1 | 0 | 0 |
| New.CleanUp.ReferenceOTU73596 | 0 | 1 | 0 | 1 | 0 | 0 | 0 |
| New.CleanUp.ReferenceOTU73643 | 0 | 1 | 1 | 1 | 1 | 1 | 1 |
| New.CleanUp.ReferenceOTU74338 | 0 | 0 | 0 | 1 | 1 | 0 | 0 |
| New.CleanUp.ReferenceOTU74603 | 0 | 0 | 1 | 0 | 0 | 0 | 0 |
| New.CleanUp.ReferenceOTU74801 | 0 | 1 | 0 | 0 | 1 | 1 | 0 |
| New.CleanUp.ReferenceOTU75167 | 0 | 0 | 1 | 0 | 0 | 0 | 0 |
| New.CleanUp.ReferenceOTU75777 | 0 | 1 | 0 | 1 | 1 | 1 | 1 |
| New.CleanUp.ReferenceOTU77749 | 0 | 1 | 0 | 0 | 0 | 0 | 0 |
| New.CleanUp.ReferenceOTU78297 | 0 | 1 | 1 | 0 | 1 | 1 | 0 |
| New.CleanUp.ReferenceOTU79080 | 0 | 0 | 0 | 0 | 0 | 0 | 1 |
| New.CleanUp.ReferenceOTU80311 | 0 | 0 | 0 | 0 | 1 | 0 | 0 |
| New.CleanUp.ReferenceOTU80681 | 0 | 1 | 0 | 0 | 1 | 0 | 1 |
| New.CleanUp.ReferenceOTU72580 | 0 | 1 | 0 | 1 | 0 | 0 | 0 |
| GAPN01002113.1.1308 | 0 | 1 | 1 | 1 | 0 | 1 | 1 |
| GAPN01002964.889.2435 | 0 | 1 | 1 | 1 | 1 | 1 | 1 |
| AY485455.1.1787 | 0 | 1 | 0 | 1 | 0 | 1 | 0 |
| GAPN01002893.1.1362 | 0 | 1 | 1 | 1 | 0 | 1 | 1 |
| New.ReferenceOTU43 | 0 | 1 | 1 | 1 | 0 | 1 | 1 |
| New.CleanUp.ReferenceOTU4868 | 0 | 1 | 1 | 1 | 0 | 1 | 0 |
| New.CleanUp.ReferenceOTU5378 | 0 | 1 | 0 | 0 | 0 | 0 | 0 |
| New.CleanUp.ReferenceOTU5618 | 0 | 1 | 0 | 0 | 0 | 0 | 0 |
| New.CleanUp.ReferenceOTU5665 | 0 | 1 | 0 | 0 | 0 | 0 | 0 |
| New.CleanUp.ReferenceOTU7195 | 0 | 1 | 0 | 0 | 0 | 1 | 0 |
| New.CleanUp.ReferenceOTU8143 | 0 | 1 | 0 | 1 | 0 | 1 | 0 |
| New.CleanUp.ReferenceOTU8951 | 0 | 1 | 1 | 0 | 0 | 1 | 1 |
| New.CleanUp.ReferenceOTU9597 | 0 | 1 | 0 | 0 | 0 | 1 | 0 |
| New.CleanUp.ReferenceOTU9715 | 0 | 1 | 0 | 1 | 0 | 1 | 1 |
| New.CleanUp.ReferenceOTU12756 | 0 | 1 | 0 | 0 | 0 | 1 | 0 |
| New.CleanUp.ReferenceOTU13446 | 0 | 1 | 1 | 1 | 0 | 0 | 0 |
| New.CleanUp.ReferenceOTU14598 | 0 | 1 | 0 | 0 | 0 | 1 | 1 |
| New.CleanUp.ReferenceOTU15324 | 0 | 1 | 0 | 1 | 0 | 1 | 1 |
| New.CleanUp.ReferenceOTU15779 | 0 | 1 | 0 | 1 | 0 | 1 | 1 |
| New.CleanUp.ReferenceOTU17007 | 0 | 1 | 1 | 0 | 0 | 1 | 1 |
| New.CleanUp.ReferenceOTU20032 | 0 | 0 | 1 | 1 | 0 | 1 | 0 |
| New.CleanUp.ReferenceOTU24224 | 0 | 1 | 0 | 1 | 0 | 0 | 0 |
| New.CleanUp.ReferenceOTU26087 | 0 | 1 | 0 | 1 | 0 | 0 | 0 |
| New.CleanUp.ReferenceOTU26224 | 0 | 1 | 0 | 0 | 0 | 1 | 0 |
| New.CleanUp.ReferenceOTU26848 | 0 | 1 | 0 | 1 | 1 | 1 | 0 |
| New.CleanUp.ReferenceOTU28510 | 0 | 1 | 1 | 0 | 0 | 0 | 1 |
| New.CleanUp.ReferenceOTU30546 | 0 | 1 | 0 | 0 | 0 | 0 | 0 |
| New.CleanUp.ReferenceOTU30984 | 0 | 1 | 0 | 0 | 0 | 0 | 1 |
| New.CleanUp.ReferenceOTU36146 | 0 | 1 | 0 | 0 | 0 | 0 | 0 |
| New.CleanUp.ReferenceOTU36184 | 0 | 0 | 1 | 0 | 0 | 1 | 0 |
| New.CleanUp.ReferenceOTU42779 | 0 | 1 | 0 | 0 | 0 | 0 | 1 |
| New.CleanUp.ReferenceOTU46667 | 0 | 0 | 0 | 1 | 0 | 1 | 1 |
| New.CleanUp.ReferenceOTU48479 | 0 | 1 | 0 | 1 | 1 | 1 | 0 |
| New.CleanUp.ReferenceOTU51330 | 0 | 0 | 0 | 1 | 0 | 1 | 0 |
| New.CleanUp.ReferenceOTU53547 | 0 | 1 | 1 | 0 | 0 | 1 | 0 |
| New.CleanUp.ReferenceOTU57558 | 0 | 1 | 0 | 1 | 1 | 0 | 0 |
| New.CleanUp.ReferenceOTU57567 | 0 | 1 | 0 | 0 | 0 | 1 | 0 |
| New.CleanUp.ReferenceOTU57924 | 0 | 1 | 0 | 1 | 0 | 0 | 0 |
| New.CleanUp.ReferenceOTU59078 | 0 | 1 | 0 | 1 | 0 | 0 | 0 |
| New.CleanUp.ReferenceOTU60882 | 0 | 1 | 0 | 0 | 0 | 0 | 0 |
| New.CleanUp.ReferenceOTU62692 | 0 | 1 | 0 | 0 | 0 | 1 | 0 |
| New.CleanUp.ReferenceOTU63101 | 0 | 1 | 0 | 0 | 0 | 0 | 0 |
| New.CleanUp.ReferenceOTU63118 | 0 | 1 | 0 | 1 | 0 | 0 | 0 |
| New.CleanUp.ReferenceOTU66746 | 0 | 1 | 0 | 0 | 0 | 1 | 0 |
| New.CleanUp.ReferenceOTU67354 | 0 | 1 | 0 | 1 | 0 | 1 | 0 |
| New.CleanUp.ReferenceOTU67380 | 0 | 0 | 0 | 1 | 0 | 0 | 0 |
| New.CleanUp.ReferenceOTU67661 | 0 | 1 | 0 | 0 | 0 | 1 | 0 |
| New.CleanUp.ReferenceOTU68571 | 1 | 1 | 0 | 1 | 0 | 1 | 1 |
| New.CleanUp.ReferenceOTU70157 | 0 | 1 | 0 | 0 | 0 | 0 | 0 |
| New.CleanUp.ReferenceOTU70955 | 0 | 1 | 0 | 1 | 0 | 0 | 0 |
| New.CleanUp.ReferenceOTU75046 | 1 | 1 | 0 | 1 | 0 | 0 | 0 |
| New.CleanUp.ReferenceOTU76103 | 0 | 1 | 0 | 0 | 0 | 1 | 0 |
| New.CleanUp.ReferenceOTU77863 | 0 | 1 | 1 | 0 | 0 | 1 | 0 |
| New.CleanUp.ReferenceOTU80938 | 0 | 1 | 0 | 1 | 0 | 1 | 0 |
| New.CleanUp.ReferenceOTU36361 | 0 | 1 | 0 | 0 | 0 | 0 | 0 |
| New.CleanUp.ReferenceOTU56949 | 0 | 1 | 0 | 0 | 0 | 0 | 0 |
| AY179994.1.1783 | 0 | 1 | 0 | 1 | 1 | 1 | 0 |
| KC771176.1.1743 | 0 | 1 | 1 | 1 | 0 | 1 | 0 |
| New.ReferenceOTU104 | 1 | 1 | 1 | 1 | 0 | 1 | 1 |
| New.ReferenceOTU142 | 0 | 1 | 1 | 0 | 1 | 1 | 0 |
| New.ReferenceOTU275 | 0 | 1 | 0 | 1 | 1 | 1 | 0 |
| New.ReferenceOTU13 | 0 | 1 | 0 | 1 | 1 | 1 | 0 |
| New.ReferenceOTU165 | 1 | 1 | 0 | 1 | 1 | 1 | 1 |
| New.CleanUp.ReferenceOTU134 | 0 | 1 | 0 | 0 | 0 | 0 | 1 |
| New.CleanUp.ReferenceOTU1008 | 0 | 1 | 0 | 1 | 0 | 1 | 0 |
| New.CleanUp.ReferenceOTU2530 | 0 | 1 | 1 | 0 | 1 | 1 | 0 |
| New.CleanUp.ReferenceOTU12664 | 0 | 1 | 0 | 0 | 0 | 0 | 0 |
| New.CleanUp.ReferenceOTU20126 | 0 | 1 | 0 | 0 | 0 | 1 | 0 |
| New.CleanUp.ReferenceOTU22243 | 0 | 1 | 0 | 0 | 1 | 1 | 0 |
| New.CleanUp.ReferenceOTU22743 | 0 | 0 | 0 | 0 | 1 | 1 | 0 |
| New.CleanUp.ReferenceOTU28515 | 1 | 1 | 1 | 1 | 0 | 0 | 0 |
| New.CleanUp.ReferenceOTU32689 | 0 | 1 | 0 | 0 | 0 | 0 | 0 |
| New.CleanUp.ReferenceOTU33741 | 0 | 0 | 0 | 0 | 1 | 1 | 0 |
| New.CleanUp.ReferenceOTU33930 | 0 | 1 | 0 | 1 | 1 | 1 | 1 |
| New.CleanUp.ReferenceOTU40034 | 1 | 0 | 0 | 1 | 0 | 1 | 0 |
| New.CleanUp.ReferenceOTU40449 | 0 | 1 | 0 | 0 | 0 | 0 | 0 |
| New.CleanUp.ReferenceOTU43988 | 1 | 1 | 1 | 1 | 1 | 1 | 1 |
| New.CleanUp.ReferenceOTU51454 | 0 | 1 | 0 | 1 | 0 | 0 | 0 |
| New.CleanUp.ReferenceOTU52100 | 0 | 1 | 0 | 0 | 0 | 0 | 0 |
| New.CleanUp.ReferenceOTU52540 | 0 | 1 | 1 | 1 | 1 | 1 | 1 |
| New.CleanUp.ReferenceOTU53368 | 0 | 1 | 0 | 0 | 1 | 0 | 0 |
| New.CleanUp.ReferenceOTU57422 | 0 | 1 | 0 | 0 | 1 | 1 | 0 |
| New.CleanUp.ReferenceOTU71896 | 0 | 1 | 0 | 0 | 0 | 1 | 0 |
| New.CleanUp.ReferenceOTU75241 | 1 | 1 | 1 | 1 | 0 | 0 | 0 |
| New.CleanUp.ReferenceOTU75969 | 1 | 1 | 1 | 1 | 1 | 1 | 1 |
| New.CleanUp.ReferenceOTU77944 | 0 | 1 | 0 | 0 | 0 | 0 | 0 |
| New.CleanUp.ReferenceOTU79106 | 0 | 0 | 0 | 0 | 1 | 0 | 0 |
| New.CleanUp.ReferenceOTU79718 | 0 | 1 | 0 | 1 | 1 | 1 | 1 |
| GU385575.1.1705 | 0 | 1 | 0 | 1 | 0 | 1 | 0 |
| LC054952.1.1822 | 1 | 1 | 1 | 1 | 0 | 0 | 0 |
| LC192339.1.1466 | 0 | 1 | 0 | 1 | 0 | 1 | 0 |
| GU385607.1.1249 | 1 | 1 | 1 | 1 | 0 | 1 | 0 |
| JN090863.1.1783 | 1 | 1 | 0 | 0 | 0 | 0 | 0 |
| New.CleanUp.ReferenceOTU22475 | 0 | 1 | 0 | 1 | 0 | 0 | 0 |
| New.CleanUp.ReferenceOTU48197 | 0 | 1 | 0 | 0 | 0 | 0 | 0 |
| New.CleanUp.ReferenceOTU48726 | 0 | 1 | 0 | 0 | 0 | 0 | 0 |
| New.CleanUp.ReferenceOTU51943 | 0 | 1 | 0 | 1 | 0 | 1 | 1 |
| New.CleanUp.ReferenceOTU56638 | 0 | 1 | 0 | 1 | 0 | 1 | 1 |
| New.CleanUp.ReferenceOTU58032 | 0 | 0 | 0 | 1 | 0 | 0 | 0 |
| New.CleanUp.ReferenceOTU77969 | 0 | 1 | 0 | 1 | 0 | 1 | 0 |
| AY256290.1.1226 | 0 | 1 | 0 | 1 | 0 | 0 | 0 |
| AY256263.1.1226 | 0 | 1 | 1 | 1 | 0 | 1 | 1 |
| New.CleanUp.ReferenceOTU50340 | 0 | 1 | 1 | 1 | 0 | 1 | 1 |
| New.CleanUp.ReferenceOTU57948 | 0 | 0 | 0 | 0 | 0 | 1 | 0 |
| AY882488.1.1253 | 0 | 1 | 1 | 1 | 0 | 0 | 0 |
| KJ577847.1.1609 | 0 | 1 | 1 | 1 | 0 | 1 | 0 |
| New.ReferenceOTU36 | 0 | 1 | 1 | 1 | 1 | 1 | 0 |
| New.CleanUp.ReferenceOTU1857 | 0 | 0 | 0 | 1 | 0 | 0 | 0 |
| New.CleanUp.ReferenceOTU3787 | 0 | 1 | 0 | 1 | 0 | 0 | 0 |
| New.CleanUp.ReferenceOTU4497 | 0 | 0 | 0 | 1 | 0 | 0 | 0 |
| New.CleanUp.ReferenceOTU8825 | 0 | 1 | 0 | 1 | 0 | 0 | 0 |
| New.CleanUp.ReferenceOTU11152 | 0 | 1 | 1 | 1 | 0 | 0 | 0 |
| New.CleanUp.ReferenceOTU11707 | 0 | 1 | 0 | 1 | 0 | 0 | 0 |
| New.CleanUp.ReferenceOTU11969 | 0 | 0 | 0 | 1 | 0 | 0 | 0 |
| New.CleanUp.ReferenceOTU16743 | 0 | 0 | 0 | 1 | 0 | 0 | 0 |
| New.CleanUp.ReferenceOTU23953 | 0 | 1 | 0 | 1 | 0 | 0 | 0 |
| New.CleanUp.ReferenceOTU24518 | 0 | 1 | 0 | 1 | 0 | 0 | 0 |
| New.CleanUp.ReferenceOTU25987 | 0 | 0 | 0 | 1 | 0 | 0 | 0 |
| New.CleanUp.ReferenceOTU27993 | 0 | 1 | 0 | 1 | 0 | 1 | 0 |
| New.CleanUp.ReferenceOTU30211 | 0 | 0 | 0 | 1 | 0 | 0 | 0 |
| New.CleanUp.ReferenceOTU32585 | 0 | 1 | 0 | 1 | 0 | 0 | 0 |
| New.CleanUp.ReferenceOTU33035 | 0 | 1 | 1 | 1 | 0 | 0 | 0 |
| New.CleanUp.ReferenceOTU39784 | 0 | 0 | 0 | 1 | 0 | 0 | 0 |
| New.CleanUp.ReferenceOTU40640 | 0 | 1 | 0 | 1 | 0 | 0 | 0 |
| New.CleanUp.ReferenceOTU42027 | 0 | 0 | 0 | 1 | 0 | 0 | 0 |
| New.CleanUp.ReferenceOTU42287 | 0 | 1 | 0 | 1 | 0 | 0 | 0 |
| New.CleanUp.ReferenceOTU42401 | 0 | 0 | 0 | 1 | 0 | 0 | 0 |
| New.CleanUp.ReferenceOTU47699 | 0 | 0 | 0 | 1 | 0 | 0 | 0 |
| New.CleanUp.ReferenceOTU50030 | 0 | 0 | 0 | 1 | 0 | 0 | 0 |
| New.CleanUp.ReferenceOTU56637 | 0 | 1 | 0 | 1 | 0 | 0 | 0 |
| New.CleanUp.ReferenceOTU56963 | 0 | 0 | 0 | 1 | 0 | 0 | 0 |
| New.CleanUp.ReferenceOTU58789 | 0 | 1 | 0 | 1 | 0 | 0 | 0 |
| New.CleanUp.ReferenceOTU59956 | 0 | 0 | 0 | 1 | 0 | 0 | 0 |
| New.CleanUp.ReferenceOTU61938 | 0 | 0 | 0 | 1 | 0 | 0 | 0 |
| New.CleanUp.ReferenceOTU66331 | 0 | 1 | 0 | 1 | 0 | 0 | 0 |
| AJ535163.1.1804 | 0 | 1 | 0 | 0 | 0 | 0 | 0 |
| New.CleanUp.ReferenceOTU44170 | 0 | 1 | 0 | 0 | 0 | 1 | 0 |
| New.CleanUp.ReferenceOTU64642 | 0 | 1 | 0 | 0 | 0 | 1 | 0 |
| New.CleanUp.ReferenceOTU78596 | 0 | 0 | 0 | 1 | 0 | 1 | 0 |
| HM749944.1.1542 | 0 | 0 | 0 | 1 | 0 | 1 | 0 |
| AM746974.1.1781 | 0 | 0 | 0 | 1 | 0 | 1 | 1 |
| New.CleanUp.ReferenceOTU35419 | 0 | 1 | 0 | 1 | 0 | 1 | 0 |
| New.CleanUp.ReferenceOTU53720 | 0 | 1 | 0 | 1 | 0 | 1 | 0 |
| JX413548.1.1619 | 0 | 0 | 0 | 1 | 0 | 1 | 0 |
| New.CleanUp.ReferenceOTU44783 | 0 | 0 | 1 | 1 | 1 | 0 | 1 |
| New.CleanUp.ReferenceOTU49206 | 0 | 1 | 0 | 1 | 0 | 0 | 1 |
| New.CleanUp.ReferenceOTU50599 | 0 | 1 | 0 | 0 | 0 | 0 | 0 |
| New.CleanUp.ReferenceOTU69516 | 0 | 1 | 0 | 0 | 0 | 1 | 0 |
| New.CleanUp.ReferenceOTU80794 | 0 | 1 | 0 | 0 | 0 | 1 | 0 |
| New.ReferenceOTU157 | 1 | 1 | 1 | 1 | 0 | 1 | 1 |
| New.CleanUp.ReferenceOTU17046 | 0 | 1 | 0 | 1 | 1 | 1 | 1 |
| New.CleanUp.ReferenceOTU34635 | 0 | 0 | 0 | 1 | 0 | 1 | 1 |
| New.CleanUp.ReferenceOTU38340 | 0 | 0 | 0 | 1 | 0 | 0 | 0 |
| New.CleanUp.ReferenceOTU40139 | 0 | 0 | 1 | 1 | 0 | 0 | 0 |
| New.CleanUp.ReferenceOTU46478 | 0 | 1 | 1 | 1 | 0 | 1 | 1 |
| New.CleanUp.ReferenceOTU47259 | 0 | 0 | 0 | 1 | 0 | 1 | 0 |
| New.CleanUp.ReferenceOTU49813 | 0 | 0 | 1 | 1 | 0 | 0 | 1 |
| New.CleanUp.ReferenceOTU62145 | 0 | 0 | 0 | 1 | 0 | 1 | 1 |
| New.CleanUp.ReferenceOTU74663 | 0 | 1 | 1 | 1 | 0 | 1 | 1 |
| New.CleanUp.ReferenceOTU78175 | 0 | 0 | 0 | 1 | 0 | 1 | 1 |
| EF423409.1.1783 | 0 | 1 | 1 | 1 | 0 | 1 | 1 |
| New.CleanUp.ReferenceOTU40171 | 0 | 1 | 0 | 0 | 0 | 0 | 0 |
| New.CleanUp.ReferenceOTU35803 | 0 | 0 | 0 | 1 | 0 | 1 | 1 |
| New.CleanUp.ReferenceOTU38176 | 0 | 0 | 0 | 1 | 0 | 1 | 1 |
| New.CleanUp.ReferenceOTU60883 | 0 | 0 | 0 | 1 | 0 | 1 | 0 |
| New.CleanUp.ReferenceOTU74478 | 0 | 0 | 0 | 1 | 0 | 1 | 0 |
| New.CleanUp.ReferenceOTU1949 | 0 | 1 | 1 | 1 | 0 | 0 | 0 |
| New.CleanUp.ReferenceOTU17345 | 0 | 1 | 1 | 0 | 0 | 1 | 0 |
| New.CleanUp.ReferenceOTU28057 | 0 | 1 | 0 | 0 | 0 | 0 | 0 |
| New.CleanUp.ReferenceOTU31784 | 0 | 1 | 0 | 0 | 0 | 0 | 0 |
| New.CleanUp.ReferenceOTU31874 | 0 | 0 | 0 | 1 | 0 | 0 | 0 |
| New.CleanUp.ReferenceOTU42269 | 0 | 1 | 1 | 0 | 0 | 1 | 0 |
| New.CleanUp.ReferenceOTU43484 | 0 | 0 | 0 | 1 | 0 | 0 | 0 |
| New.CleanUp.ReferenceOTU44158 | 0 | 1 | 1 | 0 | 0 | 1 | 0 |
| New.CleanUp.ReferenceOTU47147 | 0 | 1 | 1 | 1 | 0 | 1 | 0 |
| New.CleanUp.ReferenceOTU56758 | 0 | 1 | 1 | 1 | 0 | 1 | 0 |
| New.CleanUp.ReferenceOTU60333 | 0 | 1 | 1 | 0 | 0 | 0 | 0 |
| New.CleanUp.ReferenceOTU61901 | 0 | 1 | 1 | 0 | 0 | 1 | 0 |
| New.CleanUp.ReferenceOTU73666 | 0 | 1 | 0 | 0 | 0 | 0 | 0 |
| AB769958.1.1763 | 0 | 1 | 1 | 1 | 0 | 1 | 0 |
| New.CleanUp.ReferenceOTU7351 | 0 | 1 | 0 | 1 | 0 | 1 | 0 |
| New.CleanUp.ReferenceOTU20294 | 0 | 0 | 0 | 0 | 0 | 1 | 0 |
| New.CleanUp.ReferenceOTU23535 | 0 | 1 | 0 | 1 | 0 | 1 | 0 |
| New.CleanUp.ReferenceOTU25877 | 0 | 1 | 0 | 0 | 0 | 1 | 1 |
| New.CleanUp.ReferenceOTU26357 | 0 | 1 | 0 | 0 | 0 | 0 | 0 |
| New.CleanUp.ReferenceOTU38318 | 0 | 1 | 1 | 1 | 0 | 1 | 1 |
| New.CleanUp.ReferenceOTU46304 | 0 | 1 | 0 | 0 | 0 | 0 | 0 |
| New.CleanUp.ReferenceOTU46708 | 0 | 1 | 0 | 1 | 0 | 0 | 0 |
| New.CleanUp.ReferenceOTU48541 | 0 | 1 | 0 | 0 | 0 | 1 | 0 |
| New.CleanUp.ReferenceOTU54146 | 0 | 1 | 0 | 1 | 0 | 1 | 0 |
| New.CleanUp.ReferenceOTU54672 | 0 | 0 | 0 | 0 | 0 | 1 | 0 |
| New.CleanUp.ReferenceOTU57942 | 0 | 1 | 0 | 0 | 0 | 0 | 0 |
| New.CleanUp.ReferenceOTU60245 | 0 | 1 | 0 | 0 | 0 | 1 | 0 |
| New.CleanUp.ReferenceOTU65718 | 0 | 0 | 0 | 0 | 0 | 1 | 0 |
| New.CleanUp.ReferenceOTU69407 | 0 | 1 | 0 | 1 | 0 | 0 | 0 |
| New.CleanUp.ReferenceOTU75269 | 0 | 1 | 0 | 1 | 0 | 0 | 0 |
| New.CleanUp.ReferenceOTU75282 | 0 | 1 | 0 | 0 | 0 | 1 | 0 |
| New.CleanUp.ReferenceOTU81096 | 0 | 0 | 1 | 0 | 0 | 1 | 0 |
| GU385674.1.1694 | 1 | 1 | 1 | 1 | 0 | 1 | 1 |
| GU385694.1.1683 | 0 | 1 | 1 | 1 | 0 | 1 | 0 |
| KU561183.1.1710 | 1 | 1 | 1 | 1 | 1 | 1 | 1 |
| GU385677.1.1695 | 1 | 1 | 1 | 0 | 0 | 1 | 1 |
| GU385686.1.1763 | 0 | 1 | 1 | 0 | 0 | 1 | 0 |
| AM743094.1.1607 | 0 | 1 | 1 | 1 | 0 | 1 | 1 |
| AY256254.1.1223 | 0 | 1 | 0 | 0 | 0 | 1 | 0 |
| KU561109.1.1708 | 1 | 1 | 1 | 1 | 0 | 1 | 1 |
| New.ReferenceOTU283 | 1 | 1 | 1 | 1 | 0 | 1 | 1 |
| New.ReferenceOTU61 | 1 | 1 | 1 | 1 | 1 | 1 | 1 |
| New.CleanUp.ReferenceOTU151 | 1 | 1 | 1 | 0 | 1 | 1 | 1 |
| New.CleanUp.ReferenceOTU1075 | 0 | 1 | 0 | 1 | 0 | 1 | 0 |
| New.CleanUp.ReferenceOTU1323 | 0 | 0 | 0 | 0 | 0 | 1 | 0 |
| New.CleanUp.ReferenceOTU3575 | 0 | 0 | 0 | 1 | 0 | 1 | 0 |
| New.CleanUp.ReferenceOTU6006 | 1 | 0 | 0 | 1 | 0 | 1 | 0 |
| New.CleanUp.ReferenceOTU7553 | 0 | 1 | 0 | 0 | 0 | 0 | 0 |
| New.CleanUp.ReferenceOTU7761 | 1 | 1 | 1 | 1 | 1 | 1 | 1 |
| New.CleanUp.ReferenceOTU8354 | 0 | 1 | 1 | 1 | 0 | 0 | 0 |
| New.CleanUp.ReferenceOTU8697 | 0 | 0 | 0 | 1 | 0 | 0 | 0 |
| New.CleanUp.ReferenceOTU8824 | 0 | 0 | 0 | 0 | 0 | 1 | 0 |
| New.CleanUp.ReferenceOTU8977 | 0 | 1 | 0 | 0 | 0 | 1 | 0 |
| New.CleanUp.ReferenceOTU11163 | 0 | 0 | 1 | 0 | 0 | 1 | 0 |
| New.CleanUp.ReferenceOTU11547 | 0 | 0 | 0 | 0 | 0 | 1 | 0 |
| New.CleanUp.ReferenceOTU11617 | 0 | 1 | 0 | 0 | 0 | 0 | 0 |
| New.CleanUp.ReferenceOTU11859 | 0 | 1 | 1 | 1 | 1 | 1 | 1 |
| New.CleanUp.ReferenceOTU12270 | 0 | 1 | 0 | 0 | 0 | 1 | 0 |
| New.CleanUp.ReferenceOTU12581 | 0 | 1 | 0 | 0 | 0 | 1 | 1 |
| New.CleanUp.ReferenceOTU13109 | 0 | 1 | 0 | 1 | 1 | 1 | 0 |
| New.CleanUp.ReferenceOTU13630 | 0 | 0 | 0 | 0 | 0 | 1 | 0 |
| New.CleanUp.ReferenceOTU13690 | 1 | 1 | 0 | 1 | 0 | 1 | 1 |
| New.CleanUp.ReferenceOTU14857 | 0 | 1 | 0 | 1 | 0 | 1 | 1 |
| New.CleanUp.ReferenceOTU14874 | 0 | 1 | 0 | 0 | 0 | 1 | 0 |
| New.CleanUp.ReferenceOTU15268 | 0 | 1 | 0 | 1 | 0 | 0 | 0 |
| New.CleanUp.ReferenceOTU15399 | 0 | 0 | 0 | 0 | 0 | 1 | 0 |
| New.CleanUp.ReferenceOTU16635 | 0 | 1 | 0 | 1 | 0 | 0 | 0 |
| New.CleanUp.ReferenceOTU17060 | 0 | 1 | 0 | 1 | 0 | 1 | 0 |
| New.CleanUp.ReferenceOTU18198 | 1 | 1 | 1 | 1 | 0 | 1 | 1 |
| New.CleanUp.ReferenceOTU18872 | 0 | 1 | 0 | 0 | 0 | 0 | 0 |
| New.CleanUp.ReferenceOTU19802 | 0 | 1 | 0 | 1 | 0 | 0 | 0 |
| New.CleanUp.ReferenceOTU22176 | 0 | 0 | 0 | 0 | 0 | 1 | 0 |
| New.CleanUp.ReferenceOTU23135 | 1 | 1 | 1 | 1 | 1 | 1 | 1 |
| New.CleanUp.ReferenceOTU23377 | 0 | 1 | 0 | 0 | 0 | 1 | 0 |
| New.CleanUp.ReferenceOTU24395 | 0 | 1 | 1 | 1 | 1 | 1 | 1 |
| New.CleanUp.ReferenceOTU25895 | 0 | 0 | 0 | 0 | 0 | 1 | 0 |
| New.CleanUp.ReferenceOTU26061 | 0 | 1 | 1 | 0 | 0 | 1 | 0 |
| New.CleanUp.ReferenceOTU27054 | 0 | 0 | 1 | 0 | 0 | 1 | 0 |
| New.CleanUp.ReferenceOTU30481 | 1 | 1 | 0 | 1 | 0 | 1 | 0 |
| New.CleanUp.ReferenceOTU30789 | 0 | 1 | 0 | 1 | 0 | 0 | 0 |
| New.CleanUp.ReferenceOTU31129 | 0 | 1 | 1 | 1 | 0 | 1 | 1 |
| New.CleanUp.ReferenceOTU32455 | 0 | 1 | 1 | 1 | 0 | 1 | 0 |
| New.CleanUp.ReferenceOTU33269 | 1 | 1 | 0 | 0 | 0 | 1 | 0 |
| New.CleanUp.ReferenceOTU33627 | 0 | 1 | 1 | 0 | 0 | 0 | 1 |
| New.CleanUp.ReferenceOTU34168 | 0 | 1 | 0 | 0 | 0 | 1 | 0 |
| New.CleanUp.ReferenceOTU35169 | 0 | 1 | 0 | 0 | 0 | 1 | 0 |
| New.CleanUp.ReferenceOTU35221 | 0 | 0 | 0 | 1 | 0 | 1 | 0 |
| New.CleanUp.ReferenceOTU35561 | 0 | 1 | 0 | 0 | 0 | 0 | 0 |
| New.CleanUp.ReferenceOTU37385 | 0 | 0 | 0 | 0 | 0 | 1 | 1 |
| New.CleanUp.ReferenceOTU39155 | 0 | 0 | 0 | 0 | 0 | 1 | 0 |
| New.CleanUp.ReferenceOTU39286 | 0 | 0 | 1 | 0 | 0 | 1 | 0 |
| New.CleanUp.ReferenceOTU40265 | 0 | 1 | 0 | 0 | 0 | 1 | 1 |
| New.CleanUp.ReferenceOTU40951 | 0 | 1 | 1 | 0 | 0 | 0 | 1 |
| New.CleanUp.ReferenceOTU43898 | 0 | 0 | 0 | 1 | 0 | 1 | 0 |
| New.CleanUp.ReferenceOTU46196 | 0 | 1 | 0 | 0 | 0 | 1 | 0 |
| New.CleanUp.ReferenceOTU46720 | 1 | 1 | 0 | 1 | 0 | 1 | 1 |
| New.CleanUp.ReferenceOTU46747 | 0 | 1 | 1 | 1 | 0 | 1 | 0 |
| New.CleanUp.ReferenceOTU47823 | 0 | 1 | 1 | 0 | 0 | 0 | 0 |
| New.CleanUp.ReferenceOTU48248 | 0 | 1 | 1 | 1 | 0 | 1 | 0 |
| New.CleanUp.ReferenceOTU49667 | 0 | 1 | 0 | 0 | 0 | 1 | 0 |
| New.CleanUp.ReferenceOTU51100 | 0 | 0 | 0 | 0 | 0 | 1 | 0 |
| New.CleanUp.ReferenceOTU51823 | 0 | 1 | 0 | 1 | 0 | 0 | 1 |
| New.CleanUp.ReferenceOTU52348 | 0 | 1 | 1 | 0 | 0 | 1 | 1 |
| New.CleanUp.ReferenceOTU52559 | 0 | 0 | 0 | 0 | 0 | 0 | 1 |
| New.CleanUp.ReferenceOTU53140 | 1 | 1 | 1 | 1 | 0 | 1 | 1 |
| New.CleanUp.ReferenceOTU53248 | 0 | 1 | 0 | 1 | 0 | 1 | 0 |
| New.CleanUp.ReferenceOTU53799 | 0 | 1 | 0 | 0 | 0 | 1 | 0 |
| New.CleanUp.ReferenceOTU53915 | 0 | 1 | 1 | 1 | 0 | 1 | 0 |
| New.CleanUp.ReferenceOTU55141 | 0 | 0 | 0 | 0 | 0 | 1 | 0 |
| New.CleanUp.ReferenceOTU56553 | 0 | 1 | 1 | 0 | 0 | 1 | 1 |
| New.CleanUp.ReferenceOTU57638 | 0 | 0 | 0 | 1 | 0 | 1 | 0 |
| New.CleanUp.ReferenceOTU57731 | 0 | 1 | 0 | 1 | 0 | 1 | 0 |
| New.CleanUp.ReferenceOTU62402 | 0 | 0 | 0 | 0 | 1 | 1 | 0 |
| New.CleanUp.ReferenceOTU63617 | 0 | 0 | 0 | 0 | 0 | 1 | 0 |
| New.CleanUp.ReferenceOTU64390 | 0 | 0 | 0 | 0 | 0 | 1 | 0 |
| New.CleanUp.ReferenceOTU65378 | 0 | 1 | 1 | 0 | 0 | 0 | 0 |
| New.CleanUp.ReferenceOTU65443 | 0 | 0 | 0 | 0 | 0 | 1 | 0 |
| New.CleanUp.ReferenceOTU67838 | 0 | 0 | 0 | 0 | 0 | 1 | 1 |
| New.CleanUp.ReferenceOTU67851 | 0 | 1 | 0 | 1 | 0 | 1 | 0 |
| New.CleanUp.ReferenceOTU67869 | 0 | 1 | 0 | 1 | 0 | 1 | 0 |
| New.CleanUp.ReferenceOTU68221 | 0 | 0 | 0 | 0 | 0 | 1 | 0 |
| New.CleanUp.ReferenceOTU68411 | 0 | 1 | 1 | 0 | 0 | 1 | 0 |
| New.CleanUp.ReferenceOTU69721 | 0 | 1 | 0 | 1 | 1 | 1 | 1 |
| New.CleanUp.ReferenceOTU70361 | 0 | 1 | 0 | 0 | 0 | 1 | 0 |
| New.CleanUp.ReferenceOTU72493 | 0 | 1 | 1 | 1 | 0 | 1 | 0 |
| New.CleanUp.ReferenceOTU73132 | 0 | 0 | 1 | 0 | 0 | 1 | 0 |
| New.CleanUp.ReferenceOTU73786 | 0 | 1 | 0 | 1 | 0 | 1 | 0 |
| New.CleanUp.ReferenceOTU74722 | 0 | 1 | 1 | 1 | 0 | 1 | 0 |
| New.CleanUp.ReferenceOTU75483 | 0 | 0 | 0 | 1 | 0 | 1 | 0 |
| New.CleanUp.ReferenceOTU76149 | 0 | 1 | 0 | 1 | 0 | 1 | 1 |
| New.CleanUp.ReferenceOTU76556 | 0 | 1 | 1 | 1 | 0 | 1 | 0 |
| New.CleanUp.ReferenceOTU78863 | 0 | 0 | 1 | 0 | 0 | 1 | 0 |
| New.CleanUp.ReferenceOTU81018 | 0 | 1 | 1 | 1 | 0 | 1 | 0 |
| AY179995.1.1787 | 1 | 1 | 1 | 1 | 1 | 1 | 1 |
| KJ961667.1.1787 | 0 | 1 | 0 | 0 | 0 | 1 | 0 |
| JQ045338.1.1716 | 1 | 1 | 1 | 1 | 0 | 1 | 1 |
| KC771170.1.1785 | 1 | 1 | 1 | 1 | 1 | 1 | 1 |
| AM501966.1.1745 | 1 | 1 | 1 | 1 | 1 | 1 | 1 |
| GQ219689.1.1778 | 1 | 1 | 1 | 0 | 0 | 1 | 1 |
| KJ961665.1.1783 | 1 | 1 | 1 | 1 | 0 | 1 | 1 |
| New.CleanUp.ReferenceOTU1427 | 0 | 1 | 0 | 1 | 0 | 0 | 0 |
| New.CleanUp.ReferenceOTU4393 | 0 | 1 | 0 | 0 | 0 | 1 | 0 |
| New.CleanUp.ReferenceOTU5067 | 0 | 0 | 0 | 0 | 0 | 1 | 0 |
| New.CleanUp.ReferenceOTU7954 | 0 | 1 | 0 | 0 | 0 | 1 | 0 |
| New.CleanUp.ReferenceOTU12824 | 0 | 0 | 0 | 0 | 0 | 1 | 0 |
| New.CleanUp.ReferenceOTU13125 | 1 | 1 | 0 | 0 | 0 | 1 | 0 |
| New.CleanUp.ReferenceOTU17148 | 0 | 1 | 0 | 0 | 0 | 1 | 0 |
| New.CleanUp.ReferenceOTU17843 | 0 | 0 | 0 | 1 | 0 | 1 | 1 |
| New.CleanUp.ReferenceOTU21814 | 0 | 0 | 0 | 0 | 0 | 1 | 0 |
| New.CleanUp.ReferenceOTU28596 | 0 | 1 | 1 | 0 | 0 | 1 | 1 |
| New.CleanUp.ReferenceOTU31153 | 0 | 1 | 0 | 0 | 0 | 0 | 0 |
| New.CleanUp.ReferenceOTU34666 | 0 | 0 | 0 | 0 | 0 | 1 | 1 |
| New.CleanUp.ReferenceOTU35873 | 0 | 1 | 0 | 0 | 0 | 1 | 0 |
| New.CleanUp.ReferenceOTU36528 | 0 | 0 | 0 | 0 | 0 | 1 | 0 |
| New.CleanUp.ReferenceOTU38458 | 0 | 1 | 0 | 0 | 0 | 1 | 1 |
| New.CleanUp.ReferenceOTU41512 | 0 | 1 | 0 | 1 | 0 | 1 | 0 |
| New.CleanUp.ReferenceOTU42139 | 0 | 1 | 0 | 1 | 0 | 0 | 0 |
| New.CleanUp.ReferenceOTU45209 | 0 | 1 | 0 | 0 | 0 | 1 | 0 |
| New.CleanUp.ReferenceOTU45432 | 0 | 1 | 0 | 1 | 0 | 1 | 0 |
| New.CleanUp.ReferenceOTU47789 | 0 | 1 | 0 | 0 | 0 | 1 | 0 |
| New.CleanUp.ReferenceOTU48072 | 0 | 0 | 0 | 0 | 0 | 1 | 0 |
| New.CleanUp.ReferenceOTU49333 | 0 | 1 | 0 | 0 | 0 | 1 | 0 |
| New.CleanUp.ReferenceOTU50368 | 0 | 1 | 0 | 0 | 0 | 1 | 0 |
| New.CleanUp.ReferenceOTU56254 | 0 | 1 | 0 | 1 | 0 | 1 | 1 |
| New.CleanUp.ReferenceOTU63326 | 0 | 1 | 1 | 0 | 1 | 1 | 1 |
| New.CleanUp.ReferenceOTU68520 | 0 | 1 | 0 | 0 | 0 | 1 | 0 |
| New.CleanUp.ReferenceOTU71174 | 0 | 1 | 1 | 0 | 0 | 1 | 0 |
| New.CleanUp.ReferenceOTU46158 | 1 | 1 | 0 | 1 | 0 | 1 | 0 |
| EF100375.1.1221 | 1 | 1 | 0 | 1 | 0 | 0 | 0 |
| U42381.1.1788 | 1 | 0 | 0 | 1 | 0 | 0 | 0 |
| New.CleanUp.ReferenceOTU35434 | 1 | 1 | 0 | 1 | 0 | 0 | 0 |
| New.CleanUp.ReferenceOTU2122 | 0 | 1 | 0 | 1 | 0 | 0 | 0 |
| EF527128.1.1769 | 1 | 1 | 0 | 0 | 0 | 0 | 0 |
| New.CleanUp.ReferenceOTU45071 | 1 | 1 | 0 | 0 | 0 | 0 | 0 |
| DQ103873.1.1810 | 1 | 1 | 0 | 1 | 0 | 1 | 0 |
| New.CleanUp.ReferenceOTU11237 | 1 | 0 | 0 | 0 | 0 | 0 | 0 |
| New.CleanUp.ReferenceOTU11296 | 1 | 0 | 0 | 0 | 0 | 0 | 0 |
| New.CleanUp.ReferenceOTU13259 | 1 | 1 | 1 | 1 | 0 | 1 | 1 |
| New.CleanUp.ReferenceOTU25483 | 1 | 0 | 0 | 0 | 0 | 0 | 0 |
| New.CleanUp.ReferenceOTU31121 | 1 | 0 | 0 | 0 | 0 | 1 | 0 |
| New.CleanUp.ReferenceOTU69316 | 1 | 0 | 0 | 0 | 0 | 0 | 0 |
| New.CleanUp.ReferenceOTU80370 | 1 | 1 | 1 | 1 | 0 | 1 | 1 |
| JQ967322.1.1672 | 1 | 0 | 0 | 1 | 0 | 1 | 1 |
| DQ310204.1.1397 | 1 | 0 | 0 | 0 | 1 | 1 | 1 |
| New.ReferenceOTU58 | 1 | 1 | 0 | 1 | 1 | 1 | 1 |
| New.CleanUp.ReferenceOTU48838 | 1 | 1 | 0 | 1 | 1 | 0 | 1 |
| New.CleanUp.ReferenceOTU72315 | 1 | 0 | 0 | 0 | 0 | 0 | 0 |
| GU823283.1.1236 | 1 | 0 | 0 | 0 | 0 | 0 | 0 |
| GU823044.1.1223 | 1 | 0 | 0 | 0 | 0 | 0 | 1 |
| AF109322.1.1762 | 1 | 1 | 0 | 0 | 0 | 0 | 0 |
| New.CleanUp.ReferenceOTU5772 | 1 | 0 | 0 | 1 | 0 | 1 | 0 |
| New.CleanUp.ReferenceOTU63159 | 1 | 0 | 0 | 0 | 0 | 0 | 0 |
| AY642717.1.1718 | 1 | 0 | 0 | 0 | 0 | 0 | 0 |
| AY919766.1.1723 | 1 | 1 | 0 | 1 | 1 | 0 | 1 |
| JQ967328.1.1651 | 1 | 0 | 0 | 0 | 0 | 1 | 0 |
| HF549063.1.1706 | 1 | 0 | 0 | 0 | 0 | 0 | 0 |
| New.CleanUp.ReferenceOTU4609 | 0 | 0 | 0 | 0 | 0 | 0 | 1 |
| New.CleanUp.ReferenceOTU24587 | 1 | 0 | 0 | 1 | 0 | 0 | 0 |
| New.CleanUp.ReferenceOTU46713 | 1 | 0 | 0 | 0 | 0 | 0 | 0 |
| New.CleanUp.ReferenceOTU75683 | 1 | 0 | 0 | 0 | 0 | 0 | 0 |
| New.CleanUp.ReferenceOTU62477 | 1 | 1 | 1 | 1 | 0 | 1 | 0 |
| L37204.1.1809 | 0 | 0 | 1 | 0 | 0 | 0 | 0 |
| New.CleanUp.ReferenceOTU76244 | 0 | 1 | 1 | 0 | 0 | 0 | 0 |
| AB695483.1.1770 | 1 | 1 | 1 | 1 | 0 | 1 | 0 |
| New.CleanUp.ReferenceOTU50597 | 1 | 1 | 1 | 1 | 0 | 1 | 0 |
| New.CleanUp.ReferenceOTU76014 | 0 | 0 | 0 | 1 | 0 | 0 | 0 |
| New.CleanUp.ReferenceOTU31754 | 0 | 0 | 0 | 0 | 0 | 1 | 0 |
| New.CleanUp.ReferenceOTU45542 | 0 | 0 | 0 | 0 | 0 | 1 | 0 |
| New.CleanUp.ReferenceOTU10771 | 0 | 1 | 0 | 1 | 0 | 1 | 0 |
| New.CleanUp.ReferenceOTU33690 | 0 | 1 | 0 | 0 | 0 | 0 | 0 |
| New.CleanUp.ReferenceOTU34052 | 0 | 1 | 0 | 0 | 0 | 0 | 0 |
| New.CleanUp.ReferenceOTU35514 | 0 | 1 | 0 | 1 | 0 | 0 | 0 |
| New.CleanUp.ReferenceOTU50623 | 0 | 1 | 0 | 1 | 0 | 1 | 0 |
| New.CleanUp.ReferenceOTU22700 | 1 | 0 | 0 | 0 | 0 | 0 | 0 |
| New.CleanUp.ReferenceOTU47186 | 0 | 1 | 0 | 0 | 0 | 0 | 0 |
| New.CleanUp.ReferenceOTU65790 | 0 | 1 | 0 | 0 | 0 | 0 | 0 |
| New.CleanUp.ReferenceOTU8838 | 1 | 1 | 1 | 0 | 0 | 0 | 0 |
| New.CleanUp.ReferenceOTU57279 | 1 | 1 | 0 | 0 | 0 | 0 | 0 |
| New.CleanUp.ReferenceOTU35156 | 1 | 0 | 0 | 0 | 0 | 0 | 0 |
| New.CleanUp.ReferenceOTU16723 | 0 | 0 | 0 | 0 | 0 | 1 | 0 |
| New.CleanUp.ReferenceOTU18123 | 0 | 1 | 0 | 0 | 0 | 1 | 0 |
| New.ReferenceOTU65 | 0 | 1 | 0 | 0 | 0 | 1 | 0 |
| New.CleanUp.ReferenceOTU10593 | 0 | 1 | 0 | 0 | 0 | 1 | 0 |
| New.CleanUp.ReferenceOTU19702 | 1 | 0 | 0 | 0 | 0 | 0 | 0 |
| New.CleanUp.ReferenceOTU80508 | 1 | 1 | 0 | 0 | 0 | 1 | 0 |
| New.CleanUp.ReferenceOTU42339 | 0 | 0 | 0 | 0 | 0 | 1 | 0 |
| JN975249.1.1617 | 0 | 0 | 0 | 0 | 0 | 1 | 0 |
| AB430609.1.1742 | 0 | 1 | 1 | 1 | 0 | 0 | 0 |
| New.CleanUp.ReferenceOTU16794 | 0 | 1 | 0 | 1 | 0 | 0 | 0 |
| New.CleanUp.ReferenceOTU27919 | 0 | 1 | 0 | 1 | 0 | 0 | 0 |
| New.CleanUp.ReferenceOTU64080 | 0 | 1 | 1 | 1 | 0 | 0 | 0 |
| New.CleanUp.ReferenceOTU66818 | 0 | 1 | 0 | 0 | 0 | 0 | 0 |
| New.CleanUp.ReferenceOTU32594 | 0 | 0 | 0 | 1 | 0 | 0 | 0 |
| New.CleanUp.ReferenceOTU72607 | 0 | 0 | 0 | 1 | 0 | 0 | 0 |
| KF925343.1.1655 | 0 | 1 | 1 | 0 | 0 | 0 | 0 |
| New.CleanUp.ReferenceOTU30072 | 0 | 1 | 1 | 0 | 0 | 0 | 0 |
| New.CleanUp.ReferenceOTU59348 | 0 | 1 | 0 | 0 | 0 | 0 | 0 |
| KC222326.1.1675 | 0 | 1 | 0 | 0 | 0 | 0 | 0 |
| New.CleanUp.ReferenceOTU2800 | 0 | 1 | 0 | 1 | 0 | 1 | 0 |
| AY179992.1.1965 | 0 | 1 | 0 | 0 | 0 | 1 | 1 |
| AY256297.1.1296 | 0 | 1 | 1 | 1 | 1 | 1 | 1 |
| KX109777.1.1762 | 1 | 1 | 1 | 1 | 1 | 1 | 0 |
| KU561103.1.1689 | 0 | 1 | 0 | 1 | 1 | 1 | 1 |
| KC222328.1.1704 | 0 | 1 | 0 | 0 | 0 | 1 | 0 |
| EF151967.1.1787 | 0 | 1 | 0 | 1 | 0 | 0 | 0 |
| New.ReferenceOTU239 | 0 | 1 | 0 | 1 | 0 | 1 | 0 |
| New.ReferenceOTU85 | 0 | 1 | 0 | 1 | 0 | 1 | 0 |
| New.CleanUp.ReferenceOTU927 | 0 | 0 | 0 | 0 | 0 | 1 | 1 |
| New.CleanUp.ReferenceOTU2872 | 0 | 0 | 0 | 0 | 0 | 1 | 0 |
| New.CleanUp.ReferenceOTU4777 | 0 | 1 | 0 | 0 | 0 | 0 | 0 |
| New.CleanUp.ReferenceOTU4990 | 0 | 0 | 0 | 0 | 0 | 1 | 0 |
| New.CleanUp.ReferenceOTU5375 | 0 | 1 | 0 | 1 | 1 | 0 | 1 |
| New.CleanUp.ReferenceOTU6873 | 0 | 1 | 0 | 0 | 0 | 0 | 0 |
| New.CleanUp.ReferenceOTU6957 | 0 | 1 | 0 | 1 | 0 | 0 | 0 |
| New.CleanUp.ReferenceOTU8566 | 1 | 1 | 0 | 0 | 0 | 1 | 0 |
| New.CleanUp.ReferenceOTU11127 | 0 | 1 | 0 | 0 | 0 | 0 | 0 |
| New.CleanUp.ReferenceOTU15327 | 0 | 1 | 0 | 1 | 0 | 0 | 0 |
| New.CleanUp.ReferenceOTU15574 | 0 | 0 | 0 | 0 | 0 | 1 | 0 |
| New.CleanUp.ReferenceOTU15781 | 0 | 1 | 0 | 0 | 1 | 1 | 1 |
| New.CleanUp.ReferenceOTU18421 | 0 | 0 | 0 | 0 | 0 | 1 | 0 |
| New.CleanUp.ReferenceOTU19602 | 0 | 1 | 0 | 0 | 0 | 1 | 0 |
| New.CleanUp.ReferenceOTU20702 | 0 | 0 | 0 | 0 | 0 | 1 | 1 |
| New.CleanUp.ReferenceOTU20767 | 0 | 0 | 0 | 1 | 0 | 0 | 0 |
| New.CleanUp.ReferenceOTU26147 | 0 | 1 | 0 | 0 | 0 | 1 | 0 |
| New.CleanUp.ReferenceOTU27422 | 0 | 1 | 1 | 1 | 0 | 0 | 0 |
| New.CleanUp.ReferenceOTU27580 | 0 | 1 | 0 | 0 | 0 | 1 | 0 |
| New.CleanUp.ReferenceOTU33975 | 0 | 1 | 0 | 1 | 0 | 0 | 0 |
| New.CleanUp.ReferenceOTU34799 | 0 | 1 | 0 | 1 | 0 | 0 | 0 |
| New.CleanUp.ReferenceOTU36560 | 0 | 1 | 0 | 1 | 0 | 0 | 0 |
| New.CleanUp.ReferenceOTU36834 | 1 | 1 | 0 | 0 | 0 | 0 | 0 |
| New.CleanUp.ReferenceOTU38930 | 0 | 0 | 0 | 0 | 0 | 1 | 0 |
| New.CleanUp.ReferenceOTU39510 | 0 | 1 | 1 | 0 | 0 | 0 | 1 |
| New.CleanUp.ReferenceOTU39895 | 1 | 1 | 1 | 1 | 0 | 0 | 0 |
| New.CleanUp.ReferenceOTU40569 | 0 | 1 | 0 | 0 | 0 | 0 | 0 |
| New.CleanUp.ReferenceOTU42194 | 0 | 1 | 0 | 1 | 0 | 1 | 0 |
| New.CleanUp.ReferenceOTU42340 | 0 | 0 | 0 | 0 | 0 | 1 | 0 |
| New.CleanUp.ReferenceOTU42586 | 0 | 1 | 0 | 0 | 0 | 1 | 0 |
| New.CleanUp.ReferenceOTU44775 | 0 | 1 | 0 | 0 | 0 | 0 | 0 |
| New.CleanUp.ReferenceOTU46664 | 0 | 1 | 0 | 1 | 0 | 0 | 0 |
| New.CleanUp.ReferenceOTU49032 | 0 | 1 | 0 | 0 | 0 | 0 | 0 |
| New.CleanUp.ReferenceOTU49475 | 0 | 1 | 1 | 0 | 0 | 0 | 0 |
| New.CleanUp.ReferenceOTU50403 | 0 | 1 | 0 | 0 | 0 | 0 | 0 |
| New.CleanUp.ReferenceOTU52321 | 0 | 1 | 0 | 1 | 0 | 1 | 1 |
| New.CleanUp.ReferenceOTU53755 | 0 | 1 | 0 | 0 | 0 | 0 | 1 |
| New.CleanUp.ReferenceOTU56810 | 0 | 1 | 0 | 1 | 0 | 0 | 0 |
| New.CleanUp.ReferenceOTU61021 | 0 | 1 | 0 | 1 | 0 | 0 | 0 |
| New.CleanUp.ReferenceOTU61708 | 0 | 0 | 0 | 0 | 0 | 1 | 0 |
| New.CleanUp.ReferenceOTU62320 | 0 | 1 | 0 | 0 | 0 | 0 | 0 |
| New.CleanUp.ReferenceOTU65017 | 0 | 1 | 0 | 1 | 1 | 0 | 0 |
| New.CleanUp.ReferenceOTU66374 | 0 | 1 | 0 | 0 | 0 | 1 | 0 |
| New.CleanUp.ReferenceOTU66581 | 0 | 0 | 0 | 0 | 0 | 1 | 0 |
| New.CleanUp.ReferenceOTU66714 | 0 | 1 | 0 | 1 | 0 | 0 | 0 |
| New.CleanUp.ReferenceOTU67591 | 0 | 1 | 0 | 1 | 1 | 1 | 1 |
| New.CleanUp.ReferenceOTU68370 | 0 | 1 | 0 | 1 | 0 | 1 | 1 |
| New.CleanUp.ReferenceOTU69523 | 0 | 1 | 0 | 0 | 0 | 0 | 0 |
| New.CleanUp.ReferenceOTU71285 | 0 | 1 | 0 | 1 | 0 | 1 | 0 |
| New.CleanUp.ReferenceOTU71449 | 0 | 1 | 0 | 0 | 0 | 0 | 0 |
| New.CleanUp.ReferenceOTU71669 | 1 | 1 | 1 | 1 | 1 | 1 | 0 |
| New.CleanUp.ReferenceOTU72425 | 0 | 0 | 0 | 1 | 0 | 0 | 0 |
| New.CleanUp.ReferenceOTU75246 | 0 | 0 | 0 | 0 | 0 | 1 | 0 |
| New.CleanUp.ReferenceOTU75601 | 0 | 1 | 0 | 0 | 0 | 0 | 0 |
| New.CleanUp.ReferenceOTU79696 | 0 | 0 | 0 | 0 | 0 | 1 | 0 |
| New.CleanUp.ReferenceOTU80101 | 1 | 1 | 1 | 1 | 0 | 1 | 0 |
| JX401243.1.1786 | 0 | 1 | 1 | 1 | 0 | 0 | 0 |
| GU823174.1.1397 | 0 | 1 | 1 | 1 | 0 | 0 | 1 |
| New.CleanUp.ReferenceOTU47429 | 0 | 1 | 1 | 1 | 0 | 0 | 1 |
| KC771156.1.1803 | 0 | 1 | 0 | 0 | 0 | 0 | 0 |
| New.CleanUp.ReferenceOTU21985 | 0 | 1 | 0 | 0 | 0 | 0 | 0 |
| AF525668.1.1730 | 0 | 1 | 0 | 0 | 0 | 0 | 0 |
| New.CleanUp.ReferenceOTU32683 | 0 | 1 | 0 | 0 | 0 | 0 | 0 |
| New.CleanUp.ReferenceOTU19903 | 0 | 0 | 0 | 0 | 0 | 1 | 0 |
| New.CleanUp.ReferenceOTU25392 | 0 | 1 | 0 | 0 | 0 | 0 | 0 |
| HQ912594.1.1747 | 0 | 0 | 1 | 0 | 0 | 0 | 0 |
| New.CleanUp.ReferenceOTU8511 | 0 | 1 | 0 | 1 | 0 | 0 | 0 |
| New.CleanUp.ReferenceOTU11325 | 0 | 0 | 1 | 0 | 0 | 0 | 0 |
| New.CleanUp.ReferenceOTU14207 | 0 | 1 | 0 | 1 | 0 | 0 | 0 |
| New.CleanUp.ReferenceOTU15515 | 0 | 1 | 0 | 1 | 0 | 0 | 0 |
| New.CleanUp.ReferenceOTU40533 | 0 | 0 | 1 | 0 | 0 | 0 | 0 |
| New.CleanUp.ReferenceOTU40784 | 0 | 1 | 0 | 0 | 0 | 1 | 0 |
| New.CleanUp.ReferenceOTU41165 | 0 | 1 | 0 | 1 | 0 | 1 | 0 |
| New.CleanUp.ReferenceOTU47908 | 0 | 0 | 0 | 1 | 0 | 1 | 0 |
| New.CleanUp.ReferenceOTU65999 | 0 | 1 | 1 | 1 | 0 | 1 | 1 |
| New.CleanUp.ReferenceOTU70688 | 0 | 1 | 0 | 1 | 0 | 0 | 0 |
| New.CleanUp.ReferenceOTU71039 | 0 | 1 | 0 | 0 | 0 | 1 | 0 |
| New.CleanUp.ReferenceOTU75916 | 0 | 1 | 0 | 1 | 0 | 1 | 1 |
| New.CleanUp.ReferenceOTU78101 | 1 | 1 | 1 | 1 | 0 | 1 | 1 |
| New.CleanUp.ReferenceOTU79441 | 0 | 1 | 0 | 1 | 0 | 0 | 0 |
| New.CleanUp.ReferenceOTU80153 | 0 | 0 | 0 | 1 | 0 | 0 | 0 |
| New.CleanUp.ReferenceOTU81097 | 0 | 1 | 1 | 1 | 0 | 1 | 1 |
| New.CleanUp.ReferenceOTU58276 | 0 | 1 | 0 | 1 | 0 | 0 | 0 |
| GU385598.1.1783 | 0 | 1 | 0 | 1 | 0 | 0 | 0 |
| AY485505.1.1904 | 0 | 1 | 1 | 1 | 0 | 1 | 1 |
| AF372748.1.1744 | 0 | 0 | 0 | 0 | 0 | 1 | 1 |
| New.CleanUp.ReferenceOTU12037 | 0 | 1 | 1 | 1 | 0 | 1 | 1 |
| New.CleanUp.ReferenceOTU62769 | 0 | 1 | 0 | 0 | 0 | 1 | 1 |
| AJ535142.1.1773 | 0 | 1 | 0 | 1 | 0 | 0 | 0 |
| AB430600.1.1779 | 0 | 1 | 0 | 0 | 0 | 0 | 0 |
| New.ReferenceOTU99 | 1 | 1 | 1 | 1 | 1 | 1 | 1 |
| New.ReferenceOTU312 | 1 | 1 | 1 | 1 | 1 | 1 | 1 |
| New.CleanUp.ReferenceOTU1927 | 0 | 1 | 0 | 1 | 1 | 0 | 0 |
| New.CleanUp.ReferenceOTU4225 | 1 | 1 | 1 | 1 | 0 | 0 | 0 |
| New.CleanUp.ReferenceOTU8596 | 0 | 1 | 0 | 0 | 0 | 0 | 0 |
| New.CleanUp.ReferenceOTU10798 | 0 | 1 | 0 | 0 | 0 | 1 | 0 |
| New.CleanUp.ReferenceOTU18513 | 1 | 1 | 0 | 1 | 0 | 0 | 0 |
| New.CleanUp.ReferenceOTU26573 | 0 | 1 | 1 | 1 | 0 | 1 | 0 |
| New.CleanUp.ReferenceOTU26905 | 0 | 1 | 0 | 0 | 0 | 1 | 0 |
| New.CleanUp.ReferenceOTU33054 | 1 | 1 | 1 | 1 | 1 | 0 | 1 |
| New.CleanUp.ReferenceOTU34071 | 1 | 1 | 1 | 0 | 0 | 1 | 0 |
| New.CleanUp.ReferenceOTU43239 | 0 | 1 | 0 | 1 | 0 | 0 | 0 |
| New.CleanUp.ReferenceOTU57853 | 0 | 1 | 0 | 1 | 0 | 0 | 0 |
| New.CleanUp.ReferenceOTU81124 | 0 | 1 | 0 | 1 | 0 | 1 | 0 |
| AM497725.1.1812 | 0 | 1 | 1 | 1 | 0 | 1 | 0 |
| AB430612.1.1756 | 0 | 1 | 0 | 1 | 0 | 0 | 0 |
| EF465473.1.1795 | 0 | 0 | 0 | 0 | 0 | 1 | 1 |
| HQ912637.1.1736 | 0 | 1 | 0 | 1 | 0 | 0 | 0 |
| New.CleanUp.ReferenceOTU15585 | 0 | 0 | 0 | 1 | 0 | 0 | 0 |
| New.CleanUp.ReferenceOTU27539 | 0 | 0 | 0 | 1 | 0 | 0 | 0 |
| New.CleanUp.ReferenceOTU58213 | 0 | 1 | 0 | 0 | 0 | 0 | 1 |
| New.CleanUp.ReferenceOTU59749 | 0 | 1 | 0 | 0 | 0 | 0 | 1 |
| AY256225.1.1310 | 0 | 1 | 0 | 1 | 0 | 1 | 1 |
| KP981383.1.1786 | 1 | 1 | 0 | 1 | 0 | 0 | 1 |
| New.ReferenceOTU117 | 1 | 1 | 1 | 1 | 1 | 1 | 1 |
| New.CleanUp.ReferenceOTU2459 | 0 | 1 | 0 | 0 | 0 | 0 | 0 |
| New.CleanUp.ReferenceOTU13731 | 0 | 0 | 1 | 1 | 0 | 1 | 1 |
| New.CleanUp.ReferenceOTU14340 | 0 | 1 | 0 | 1 | 0 | 1 | 0 |
| New.CleanUp.ReferenceOTU15652 | 0 | 1 | 0 | 1 | 0 | 0 | 0 |
| New.CleanUp.ReferenceOTU16226 | 0 | 1 | 0 | 1 | 0 | 0 | 0 |
| New.CleanUp.ReferenceOTU21365 | 0 | 0 | 1 | 1 | 0 | 1 | 1 |
| New.CleanUp.ReferenceOTU37752 | 0 | 0 | 0 | 1 | 0 | 0 | 0 |
| New.CleanUp.ReferenceOTU39591 | 0 | 1 | 1 | 1 | 0 | 0 | 0 |
| New.CleanUp.ReferenceOTU49095 | 0 | 1 | 1 | 0 | 0 | 1 | 0 |
| New.CleanUp.ReferenceOTU56483 | 0 | 0 | 1 | 0 | 0 | 0 | 0 |
| New.CleanUp.ReferenceOTU57355 | 0 | 0 | 1 | 1 | 0 | 0 | 0 |
| New.CleanUp.ReferenceOTU66685 | 0 | 1 | 0 | 1 | 0 | 0 | 0 |
| New.CleanUp.ReferenceOTU69296 | 0 | 1 | 0 | 1 | 0 | 0 | 0 |
| New.CleanUp.ReferenceOTU69768 | 0 | 1 | 0 | 0 | 0 | 0 | 0 |
| New.CleanUp.ReferenceOTU73133 | 0 | 1 | 0 | 0 | 0 | 0 | 1 |
| New.CleanUp.ReferenceOTU3798 | 0 | 1 | 0 | 1 | 0 | 0 | 0 |
| New.CleanUp.ReferenceOTU31094 | 0 | 1 | 0 | 1 | 0 | 0 | 0 |
| New.CleanUp.ReferenceOTU55691 | 1 | 1 | 0 | 0 | 0 | 0 | 0 |
| KY322505.1.1818 | 0 | 1 | 1 | 1 | 0 | 1 | 0 |
| New.ReferenceOTU344 | 1 | 1 | 1 | 1 | 0 | 1 | 0 |
| New.CleanUp.ReferenceOTU11332 | 0 | 1 | 0 | 1 | 0 | 0 | 0 |
| New.CleanUp.ReferenceOTU42576 | 0 | 1 | 0 | 0 | 0 | 0 | 0 |
| New.CleanUp.ReferenceOTU42719 | 0 | 1 | 0 | 0 | 0 | 0 | 0 |
| New.CleanUp.ReferenceOTU49573 | 0 | 1 | 0 | 1 | 0 | 0 | 0 |
| KU561172.1.1705 | 0 | 1 | 0 | 1 | 0 | 0 | 0 |
| New.ReferenceOTU280 | 1 | 1 | 0 | 0 | 0 | 1 | 0 |
| New.ReferenceOTU270 | 0 | 1 | 1 | 1 | 1 | 1 | 1 |
| New.ReferenceOTU10 | 0 | 1 | 1 | 1 | 1 | 1 | 1 |
| New.CleanUp.ReferenceOTU7953 | 0 | 1 | 1 | 1 | 1 | 1 | 1 |
| New.CleanUp.ReferenceOTU16100 | 0 | 1 | 1 | 1 | 1 | 1 | 1 |
| New.CleanUp.ReferenceOTU27995 | 0 | 0 | 0 | 1 | 0 | 0 | 0 |
| New.CleanUp.ReferenceOTU31141 | 0 | 1 | 1 | 0 | 0 | 1 | 0 |
| New.CleanUp.ReferenceOTU48746 | 0 | 1 | 0 | 0 | 0 | 1 | 0 |
| New.CleanUp.ReferenceOTU52976 | 0 | 1 | 1 | 1 | 1 | 1 | 1 |
| New.CleanUp.ReferenceOTU53110 | 0 | 1 | 0 | 1 | 0 | 0 | 0 |
| New.CleanUp.ReferenceOTU55353 | 0 | 1 | 0 | 1 | 1 | 1 | 1 |
| New.CleanUp.ReferenceOTU59182 | 0 | 1 | 0 | 0 | 0 | 1 | 0 |
| New.CleanUp.ReferenceOTU61251 | 0 | 1 | 0 | 1 | 0 | 0 | 0 |
| New.CleanUp.ReferenceOTU69609 | 0 | 1 | 0 | 1 | 0 | 0 | 0 |
| New.CleanUp.ReferenceOTU80280 | 0 | 1 | 0 | 0 | 0 | 1 | 0 |
| New.CleanUp.ReferenceOTU81172 | 0 | 1 | 1 | 0 | 0 | 0 | 0 |
| GU385676.1.1744 | 0 | 1 | 1 | 1 | 1 | 1 | 1 |
| New.CleanUp.ReferenceOTU6946 | 0 | 1 | 0 | 0 | 0 | 0 | 0 |
| New.CleanUp.ReferenceOTU16970 | 0 | 1 | 0 | 0 | 0 | 1 | 0 |
| New.CleanUp.ReferenceOTU26393 | 0 | 1 | 0 | 0 | 0 | 0 | 0 |
| New.CleanUp.ReferenceOTU28641 | 0 | 1 | 0 | 0 | 1 | 1 | 0 |
| New.CleanUp.ReferenceOTU46797 | 0 | 1 | 0 | 1 | 0 | 0 | 0 |
| New.CleanUp.ReferenceOTU71156 | 0 | 1 | 0 | 0 | 0 | 0 | 0 |
| GU385609.1.1711 | 1 | 1 | 1 | 1 | 0 | 1 | 1 |
| KU561138.1.1694 | 1 | 1 | 1 | 1 | 1 | 1 | 1 |
| AJ535190.1.1789 | 1 | 1 | 0 | 1 | 0 | 0 | 1 |
| New.ReferenceOTU242 | 0 | 1 | 1 | 1 | 1 | 1 | 1 |
| New.ReferenceOTU301 | 0 | 1 | 1 | 1 | 1 | 1 | 1 |
| New.ReferenceOTU66 | 1 | 1 | 1 | 1 | 1 | 1 | 1 |
| New.ReferenceOTU83 | 1 | 1 | 1 | 1 | 1 | 1 | 1 |
| New.ReferenceOTU361 | 0 | 1 | 0 | 0 | 0 | 0 | 0 |
| New.ReferenceOTU212 | 1 | 1 | 1 | 1 | 0 | 1 | 1 |
| New.ReferenceOTU214 | 0 | 1 | 0 | 1 | 0 | 0 | 0 |
| New.CleanUp.ReferenceOTU52 | 0 | 1 | 0 | 0 | 0 | 0 | 0 |
| New.CleanUp.ReferenceOTU141 | 0 | 1 | 0 | 1 | 0 | 0 | 0 |
| New.CleanUp.ReferenceOTU177 | 0 | 1 | 0 | 1 | 0 | 0 | 0 |
| New.CleanUp.ReferenceOTU1170 | 0 | 1 | 0 | 0 | 0 | 1 | 0 |
| New.CleanUp.ReferenceOTU1306 | 0 | 1 | 0 | 1 | 0 | 1 | 0 |
| New.CleanUp.ReferenceOTU1395 | 0 | 1 | 0 | 0 | 0 | 0 | 0 |
| New.CleanUp.ReferenceOTU1701 | 0 | 0 | 0 | 1 | 0 | 0 | 0 |
| New.CleanUp.ReferenceOTU2168 | 0 | 0 | 0 | 1 | 0 | 0 | 0 |
| New.CleanUp.ReferenceOTU2178 | 0 | 1 | 0 | 0 | 0 | 0 | 0 |
| New.CleanUp.ReferenceOTU3842 | 0 | 1 | 1 | 1 | 0 | 0 | 0 |
| New.CleanUp.ReferenceOTU5063 | 0 | 1 | 0 | 0 | 0 | 0 | 0 |
| New.CleanUp.ReferenceOTU5804 | 0 | 1 | 0 | 0 | 0 | 0 | 0 |
| New.CleanUp.ReferenceOTU6113 | 0 | 1 | 1 | 1 | 0 | 0 | 0 |
| New.CleanUp.ReferenceOTU7007 | 1 | 0 | 0 | 0 | 0 | 0 | 0 |
| New.CleanUp.ReferenceOTU7180 | 0 | 1 | 0 | 0 | 0 | 0 | 0 |
| New.CleanUp.ReferenceOTU7287 | 0 | 1 | 0 | 0 | 0 | 0 | 0 |
| New.CleanUp.ReferenceOTU7983 | 0 | 1 | 0 | 0 | 0 | 0 | 0 |
| New.CleanUp.ReferenceOTU8010 | 0 | 1 | 0 | 0 | 0 | 0 | 0 |
| New.CleanUp.ReferenceOTU8326 | 0 | 1 | 1 | 0 | 0 | 0 | 0 |
| New.CleanUp.ReferenceOTU8413 | 0 | 1 | 1 | 0 | 0 | 0 | 0 |
| New.CleanUp.ReferenceOTU8827 | 0 | 1 | 0 | 1 | 0 | 0 | 0 |
| New.CleanUp.ReferenceOTU9103 | 0 | 0 | 0 | 1 | 0 | 0 | 1 |
| New.CleanUp.ReferenceOTU9562 | 0 | 0 | 0 | 1 | 0 | 0 | 0 |
| New.CleanUp.ReferenceOTU10215 | 0 | 1 | 0 | 0 | 0 | 1 | 1 |
| New.CleanUp.ReferenceOTU11301 | 0 | 1 | 0 | 0 | 0 | 0 | 0 |
| New.CleanUp.ReferenceOTU11376 | 0 | 1 | 0 | 1 | 0 | 0 | 0 |
| New.CleanUp.ReferenceOTU11715 | 0 | 1 | 0 | 0 | 0 | 0 | 0 |
| New.CleanUp.ReferenceOTU11925 | 0 | 1 | 0 | 1 | 0 | 0 | 0 |
| New.CleanUp.ReferenceOTU11999 | 0 | 1 | 0 | 0 | 0 | 0 | 0 |
| New.CleanUp.ReferenceOTU14529 | 0 | 1 | 0 | 0 | 0 | 0 | 0 |
| New.CleanUp.ReferenceOTU14759 | 1 | 1 | 0 | 1 | 0 | 0 | 0 |
| New.CleanUp.ReferenceOTU14958 | 0 | 1 | 1 | 1 | 0 | 1 | 1 |
| New.CleanUp.ReferenceOTU15318 | 1 | 1 | 0 | 1 | 0 | 0 | 0 |
| New.CleanUp.ReferenceOTU15628 | 0 | 0 | 0 | 0 | 0 | 1 | 0 |
| New.CleanUp.ReferenceOTU16470 | 0 | 1 | 0 | 1 | 0 | 1 | 0 |
| New.CleanUp.ReferenceOTU16608 | 0 | 1 | 0 | 0 | 0 | 0 | 0 |
| New.CleanUp.ReferenceOTU17197 | 0 | 1 | 0 | 0 | 0 | 1 | 0 |
| New.CleanUp.ReferenceOTU17422 | 0 | 1 | 0 | 1 | 0 | 0 | 0 |
| New.CleanUp.ReferenceOTU17750 | 1 | 1 | 1 | 1 | 0 | 0 | 0 |
| New.CleanUp.ReferenceOTU18371 | 0 | 1 | 0 | 0 | 0 | 0 | 0 |
| New.CleanUp.ReferenceOTU19468 | 0 | 1 | 0 | 0 | 1 | 0 | 1 |
| New.CleanUp.ReferenceOTU19662 | 0 | 1 | 1 | 0 | 0 | 0 | 0 |
| New.CleanUp.ReferenceOTU20146 | 1 | 0 | 0 | 0 | 0 | 1 | 0 |
| New.CleanUp.ReferenceOTU20780 | 1 | 1 | 0 | 0 | 0 | 0 | 0 |
| New.CleanUp.ReferenceOTU21015 | 0 | 0 | 0 | 0 | 0 | 1 | 0 |
| New.CleanUp.ReferenceOTU21150 | 0 | 1 | 0 | 0 | 0 | 0 | 0 |
| New.CleanUp.ReferenceOTU21292 | 0 | 1 | 0 | 1 | 0 | 0 | 0 |
| New.CleanUp.ReferenceOTU21357 | 0 | 1 | 0 | 1 | 0 | 0 | 0 |
| New.CleanUp.ReferenceOTU21825 | 0 | 1 | 0 | 1 | 0 | 0 | 0 |
| New.CleanUp.ReferenceOTU21981 | 1 | 0 | 0 | 0 | 0 | 0 | 0 |
| New.CleanUp.ReferenceOTU22102 | 0 | 1 | 0 | 0 | 0 | 0 | 0 |
| New.CleanUp.ReferenceOTU22976 | 0 | 1 | 0 | 1 | 0 | 1 | 0 |
| New.CleanUp.ReferenceOTU23341 | 0 | 1 | 0 | 1 | 0 | 1 | 0 |
| New.CleanUp.ReferenceOTU23559 | 0 | 1 | 0 | 1 | 0 | 0 | 0 |
| New.CleanUp.ReferenceOTU24406 | 0 | 0 | 0 | 1 | 0 | 0 | 0 |
| New.CleanUp.ReferenceOTU25191 | 0 | 1 | 1 | 0 | 0 | 0 | 0 |
| New.CleanUp.ReferenceOTU25398 | 0 | 0 | 0 | 0 | 0 | 0 | 1 |
| New.CleanUp.ReferenceOTU26142 | 1 | 0 | 0 | 0 | 0 | 0 | 0 |
| New.CleanUp.ReferenceOTU28145 | 0 | 1 | 0 | 0 | 0 | 0 | 0 |
| New.CleanUp.ReferenceOTU28824 | 0 | 1 | 0 | 1 | 0 | 0 | 0 |
| New.CleanUp.ReferenceOTU28872 | 0 | 1 | 0 | 1 | 0 | 0 | 0 |
| New.CleanUp.ReferenceOTU29021 | 0 | 1 | 0 | 1 | 0 | 1 | 0 |
| New.CleanUp.ReferenceOTU29147 | 0 | 1 | 0 | 0 | 0 | 0 | 0 |
| New.CleanUp.ReferenceOTU29493 | 0 | 1 | 1 | 0 | 0 | 0 | 0 |
| New.CleanUp.ReferenceOTU29805 | 0 | 1 | 1 | 1 | 0 | 0 | 0 |
| New.CleanUp.ReferenceOTU30191 | 1 | 1 | 0 | 1 | 0 | 0 | 0 |
| New.CleanUp.ReferenceOTU30346 | 0 | 1 | 1 | 1 | 0 | 1 | 0 |
| New.CleanUp.ReferenceOTU30676 | 0 | 1 | 0 | 0 | 0 | 0 | 0 |
| New.CleanUp.ReferenceOTU32382 | 0 | 0 | 0 | 1 | 0 | 1 | 0 |
| New.CleanUp.ReferenceOTU32405 | 0 | 1 | 0 | 1 | 0 | 0 | 1 |
| New.CleanUp.ReferenceOTU32421 | 0 | 0 | 0 | 0 | 0 | 1 | 0 |
| New.CleanUp.ReferenceOTU33058 | 0 | 1 | 1 | 1 | 0 | 0 | 0 |
| New.CleanUp.ReferenceOTU33363 | 0 | 1 | 0 | 0 | 1 | 1 | 0 |
| New.CleanUp.ReferenceOTU34070 | 0 | 0 | 0 | 0 | 0 | 1 | 1 |
| New.CleanUp.ReferenceOTU34946 | 0 | 0 | 0 | 0 | 0 | 1 | 0 |
| New.CleanUp.ReferenceOTU35006 | 0 | 1 | 0 | 1 | 0 | 0 | 0 |
| New.CleanUp.ReferenceOTU35429 | 0 | 0 | 0 | 0 | 0 | 0 | 1 |
| New.CleanUp.ReferenceOTU35639 | 0 | 1 | 0 | 1 | 0 | 1 | 0 |
| New.CleanUp.ReferenceOTU35733 | 0 | 1 | 0 | 0 | 0 | 1 | 0 |
| New.CleanUp.ReferenceOTU36137 | 0 | 1 | 1 | 1 | 1 | 1 | 1 |
| New.CleanUp.ReferenceOTU36614 | 0 | 0 | 0 | 1 | 0 | 0 | 0 |
| New.CleanUp.ReferenceOTU36673 | 0 | 1 | 0 | 0 | 0 | 0 | 0 |
| New.CleanUp.ReferenceOTU36881 | 1 | 1 | 0 | 0 | 0 | 1 | 0 |
| New.CleanUp.ReferenceOTU37461 | 0 | 0 | 0 | 0 | 0 | 0 | 1 |
| New.CleanUp.ReferenceOTU37605 | 0 | 0 | 0 | 1 | 0 | 1 | 0 |
| New.CleanUp.ReferenceOTU37916 | 0 | 1 | 0 | 1 | 0 | 0 | 0 |
| New.CleanUp.ReferenceOTU38129 | 0 | 1 | 0 | 0 | 0 | 0 | 0 |
| New.CleanUp.ReferenceOTU38298 | 0 | 1 | 0 | 0 | 0 | 0 | 0 |
| New.CleanUp.ReferenceOTU38898 | 0 | 1 | 0 | 1 | 0 | 0 | 0 |
| New.CleanUp.ReferenceOTU39602 | 0 | 1 | 0 | 0 | 0 | 0 | 0 |
| New.CleanUp.ReferenceOTU39666 | 0 | 1 | 0 | 1 | 0 | 1 | 0 |
| New.CleanUp.ReferenceOTU39864 | 0 | 1 | 1 | 1 | 1 | 1 | 0 |
| New.CleanUp.ReferenceOTU40048 | 0 | 1 | 0 | 1 | 0 | 0 | 0 |
| New.CleanUp.ReferenceOTU40184 | 0 | 1 | 1 | 0 | 0 | 0 | 0 |
| New.CleanUp.ReferenceOTU40632 | 0 | 0 | 0 | 1 | 0 | 1 | 1 |
| New.CleanUp.ReferenceOTU40655 | 0 | 1 | 0 | 1 | 0 | 1 | 0 |
| New.CleanUp.ReferenceOTU40705 | 0 | 1 | 0 | 0 | 0 | 0 | 0 |
| New.CleanUp.ReferenceOTU40715 | 0 | 0 | 0 | 1 | 0 | 0 | 0 |
| New.CleanUp.ReferenceOTU40807 | 0 | 1 | 0 | 0 | 0 | 0 | 0 |
| New.CleanUp.ReferenceOTU41217 | 0 | 1 | 1 | 1 | 0 | 1 | 1 |
| New.CleanUp.ReferenceOTU42176 | 1 | 1 | 0 | 0 | 0 | 1 | 0 |
| New.CleanUp.ReferenceOTU42912 | 0 | 1 | 0 | 1 | 0 | 0 | 0 |
| New.CleanUp.ReferenceOTU43069 | 0 | 0 | 0 | 1 | 0 | 1 | 0 |
| New.CleanUp.ReferenceOTU43106 | 0 | 1 | 0 | 0 | 0 | 1 | 0 |
| New.CleanUp.ReferenceOTU43242 | 0 | 1 | 0 | 0 | 0 | 0 | 1 |
| New.CleanUp.ReferenceOTU43613 | 0 | 0 | 0 | 0 | 0 | 1 | 0 |
| New.CleanUp.ReferenceOTU43784 | 0 | 1 | 0 | 0 | 0 | 0 | 0 |
| New.CleanUp.ReferenceOTU44020 | 0 | 1 | 0 | 1 | 0 | 0 | 0 |
| New.CleanUp.ReferenceOTU44049 | 0 | 0 | 0 | 0 | 0 | 1 | 1 |
| New.CleanUp.ReferenceOTU44608 | 0 | 1 | 0 | 1 | 0 | 0 | 0 |
| New.CleanUp.ReferenceOTU45263 | 0 | 1 | 0 | 1 | 0 | 1 | 0 |
| New.CleanUp.ReferenceOTU45270 | 0 | 0 | 0 | 1 | 0 | 1 | 0 |
| New.CleanUp.ReferenceOTU45549 | 0 | 0 | 0 | 0 | 0 | 1 | 1 |
| New.CleanUp.ReferenceOTU45672 | 0 | 0 | 0 | 0 | 0 | 1 | 0 |
| New.CleanUp.ReferenceOTU45692 | 0 | 0 | 0 | 1 | 0 | 0 | 1 |
| New.CleanUp.ReferenceOTU46416 | 1 | 0 | 0 | 0 | 0 | 0 | 0 |
| New.CleanUp.ReferenceOTU47407 | 0 | 1 | 0 | 0 | 0 | 0 | 0 |
| New.CleanUp.ReferenceOTU47480 | 0 | 1 | 0 | 0 | 0 | 1 | 0 |
| New.CleanUp.ReferenceOTU47603 | 0 | 1 | 0 | 0 | 0 | 0 | 0 |
| New.CleanUp.ReferenceOTU48195 | 0 | 1 | 0 | 0 | 0 | 1 | 0 |
| New.CleanUp.ReferenceOTU48271 | 0 | 1 | 0 | 1 | 0 | 1 | 0 |
| New.CleanUp.ReferenceOTU48576 | 1 | 1 | 1 | 1 | 1 | 1 | 1 |
| New.CleanUp.ReferenceOTU48680 | 0 | 1 | 0 | 1 | 0 | 0 | 0 |
| New.CleanUp.ReferenceOTU48989 | 0 | 1 | 0 | 1 | 0 | 0 | 0 |
| New.CleanUp.ReferenceOTU50174 | 0 | 0 | 0 | 0 | 0 | 1 | 0 |
| New.CleanUp.ReferenceOTU50319 | 0 | 1 | 0 | 0 | 0 | 1 | 1 |
| New.CleanUp.ReferenceOTU50567 | 0 | 1 | 0 | 0 | 0 | 0 | 1 |
| New.CleanUp.ReferenceOTU50701 | 0 | 1 | 0 | 1 | 0 | 0 | 0 |
| New.CleanUp.ReferenceOTU51636 | 0 | 1 | 0 | 1 | 0 | 0 | 0 |
| New.CleanUp.ReferenceOTU51797 | 0 | 1 | 0 | 0 | 0 | 1 | 0 |
| New.CleanUp.ReferenceOTU52179 | 1 | 1 | 1 | 1 | 0 | 1 | 1 |
| New.CleanUp.ReferenceOTU53751 | 0 | 0 | 0 | 0 | 0 | 1 | 1 |
| New.CleanUp.ReferenceOTU53949 | 0 | 1 | 0 | 1 | 0 | 0 | 0 |
| New.CleanUp.ReferenceOTU54181 | 0 | 1 | 0 | 0 | 0 | 0 | 0 |
| New.CleanUp.ReferenceOTU54322 | 0 | 0 | 1 | 0 | 0 | 1 | 0 |
| New.CleanUp.ReferenceOTU55463 | 0 | 1 | 0 | 1 | 0 | 0 | 0 |
| New.CleanUp.ReferenceOTU55564 | 1 | 1 | 1 | 1 | 1 | 1 | 1 |
| New.CleanUp.ReferenceOTU55600 | 0 | 1 | 0 | 0 | 0 | 1 | 0 |
| New.CleanUp.ReferenceOTU56435 | 0 | 1 | 0 | 0 | 0 | 0 | 0 |
| New.CleanUp.ReferenceOTU56515 | 0 | 1 | 0 | 0 | 0 | 1 | 1 |
| New.CleanUp.ReferenceOTU56701 | 0 | 1 | 0 | 0 | 0 | 1 | 0 |
| New.CleanUp.ReferenceOTU56976 | 0 | 1 | 0 | 1 | 0 | 0 | 0 |
| New.CleanUp.ReferenceOTU56997 | 0 | 0 | 0 | 1 | 0 | 1 | 0 |
| New.CleanUp.ReferenceOTU57409 | 0 | 0 | 0 | 0 | 0 | 1 | 0 |
| New.CleanUp.ReferenceOTU57545 | 0 | 1 | 0 | 0 | 0 | 0 | 0 |
| New.CleanUp.ReferenceOTU57620 | 0 | 1 | 0 | 0 | 0 | 0 | 1 |
| New.CleanUp.ReferenceOTU57825 | 0 | 0 | 0 | 1 | 0 | 0 | 0 |
| New.CleanUp.ReferenceOTU58339 | 0 | 1 | 0 | 0 | 0 | 0 | 0 |
| New.CleanUp.ReferenceOTU58540 | 0 | 0 | 1 | 1 | 0 | 0 | 0 |
| New.CleanUp.ReferenceOTU58652 | 0 | 1 | 0 | 0 | 0 | 1 | 0 |
| New.CleanUp.ReferenceOTU58805 | 0 | 1 | 1 | 1 | 0 | 0 | 0 |
| New.CleanUp.ReferenceOTU59354 | 0 | 0 | 1 | 0 | 0 | 1 | 1 |
| New.CleanUp.ReferenceOTU59631 | 0 | 1 | 0 | 0 | 0 | 1 | 0 |
| New.CleanUp.ReferenceOTU59898 | 1 | 1 | 0 | 0 | 0 | 0 | 0 |
| New.CleanUp.ReferenceOTU59940 | 0 | 0 | 0 | 1 | 0 | 1 | 0 |
| New.CleanUp.ReferenceOTU60467 | 0 | 1 | 0 | 0 | 0 | 1 | 1 |
| New.CleanUp.ReferenceOTU60624 | 1 | 0 | 0 | 0 | 0 | 0 | 0 |
| New.CleanUp.ReferenceOTU60686 | 0 | 1 | 0 | 1 | 0 | 0 | 0 |
| New.CleanUp.ReferenceOTU61110 | 0 | 1 | 0 | 0 | 0 | 1 | 1 |
| New.CleanUp.ReferenceOTU61117 | 1 | 1 | 0 | 0 | 0 | 0 | 0 |
| New.CleanUp.ReferenceOTU63148 | 0 | 1 | 0 | 1 | 0 | 1 | 0 |
| New.CleanUp.ReferenceOTU63222 | 0 | 1 | 0 | 0 | 0 | 0 | 0 |
| New.CleanUp.ReferenceOTU63638 | 0 | 1 | 0 | 0 | 0 | 1 | 1 |
| New.CleanUp.ReferenceOTU63694 | 0 | 1 | 0 | 0 | 0 | 0 | 0 |
| New.CleanUp.ReferenceOTU64120 | 0 | 1 | 0 | 1 | 0 | 0 | 0 |
| New.CleanUp.ReferenceOTU64314 | 0 | 1 | 0 | 1 | 0 | 1 | 1 |
| New.CleanUp.ReferenceOTU64915 | 0 | 1 | 0 | 1 | 0 | 0 | 0 |
| New.CleanUp.ReferenceOTU65446 | 0 | 0 | 0 | 1 | 0 | 1 | 0 |
| New.CleanUp.ReferenceOTU65748 | 0 | 0 | 1 | 1 | 0 | 0 | 0 |
| New.CleanUp.ReferenceOTU66142 | 0 | 1 | 0 | 1 | 0 | 1 | 0 |
| New.CleanUp.ReferenceOTU66487 | 0 | 1 | 1 | 1 | 0 | 0 | 0 |
| New.CleanUp.ReferenceOTU66528 | 0 | 1 | 0 | 0 | 1 | 1 | 0 |
| New.CleanUp.ReferenceOTU67445 | 0 | 1 | 0 | 1 | 0 | 0 | 0 |
| New.CleanUp.ReferenceOTU67516 | 0 | 1 | 0 | 0 | 0 | 1 | 0 |
| New.CleanUp.ReferenceOTU67604 | 0 | 1 | 1 | 1 | 0 | 0 | 0 |
| New.CleanUp.ReferenceOTU68024 | 0 | 1 | 0 | 0 | 1 | 1 | 0 |
| New.CleanUp.ReferenceOTU68218 | 0 | 1 | 1 | 1 | 0 | 1 | 0 |
| New.CleanUp.ReferenceOTU68538 | 0 | 1 | 0 | 0 | 0 | 0 | 0 |
| New.CleanUp.ReferenceOTU69530 | 0 | 1 | 0 | 0 | 0 | 0 | 0 |
| New.CleanUp.ReferenceOTU70247 | 0 | 1 | 0 | 1 | 0 | 0 | 0 |
| New.CleanUp.ReferenceOTU70977 | 0 | 1 | 0 | 1 | 0 | 0 | 0 |
| New.CleanUp.ReferenceOTU71057 | 0 | 1 | 0 | 0 | 0 | 0 | 0 |
| New.CleanUp.ReferenceOTU71337 | 0 | 1 | 0 | 0 | 0 | 0 | 0 |
| New.CleanUp.ReferenceOTU71508 | 0 | 0 | 1 | 0 | 0 | 0 | 0 |
| New.CleanUp.ReferenceOTU72368 | 0 | 1 | 0 | 1 | 0 | 0 | 0 |
| New.CleanUp.ReferenceOTU72579 | 1 | 1 | 0 | 0 | 0 | 0 | 1 |
| New.CleanUp.ReferenceOTU72759 | 1 | 1 | 0 | 0 | 0 | 1 | 0 |
| New.CleanUp.ReferenceOTU72930 | 0 | 1 | 0 | 0 | 0 | 0 | 0 |
| New.CleanUp.ReferenceOTU72960 | 0 | 1 | 0 | 1 | 0 | 0 | 0 |
| New.CleanUp.ReferenceOTU73110 | 0 | 1 | 0 | 0 | 0 | 0 | 0 |
| New.CleanUp.ReferenceOTU73303 | 0 | 0 | 0 | 0 | 0 | 1 | 0 |
| New.CleanUp.ReferenceOTU73347 | 0 | 0 | 0 | 1 | 0 | 0 | 0 |
| New.CleanUp.ReferenceOTU74455 | 0 | 0 | 0 | 1 | 0 | 0 | 0 |
| New.CleanUp.ReferenceOTU74567 | 0 | 1 | 0 | 1 | 0 | 0 | 0 |
| New.CleanUp.ReferenceOTU75607 | 0 | 1 | 0 | 1 | 0 | 1 | 1 |
| New.CleanUp.ReferenceOTU75902 | 0 | 0 | 0 | 0 | 0 | 1 | 0 |
| New.CleanUp.ReferenceOTU76454 | 0 | 1 | 0 | 1 | 0 | 1 | 0 |
| New.CleanUp.ReferenceOTU76483 | 0 | 1 | 0 | 1 | 0 | 0 | 0 |
| New.CleanUp.ReferenceOTU76555 | 0 | 1 | 0 | 1 | 0 | 1 | 1 |
| New.CleanUp.ReferenceOTU77088 | 0 | 1 | 0 | 0 | 0 | 0 | 1 |
| New.CleanUp.ReferenceOTU77223 | 0 | 0 | 0 | 0 | 0 | 1 | 0 |
| New.CleanUp.ReferenceOTU77554 | 0 | 1 | 0 | 0 | 0 | 0 | 0 |
| New.CleanUp.ReferenceOTU78060 | 1 | 1 | 0 | 0 | 0 | 1 | 0 |
| New.CleanUp.ReferenceOTU78925 | 0 | 1 | 0 | 0 | 0 | 0 | 0 |
| New.CleanUp.ReferenceOTU78988 | 0 | 1 | 0 | 0 | 0 | 0 | 0 |
| New.CleanUp.ReferenceOTU79123 | 1 | 0 | 0 | 0 | 0 | 0 | 0 |
| New.CleanUp.ReferenceOTU79211 | 0 | 1 | 0 | 0 | 0 | 1 | 0 |
| New.CleanUp.ReferenceOTU79435 | 0 | 1 | 0 | 0 | 0 | 1 | 0 |
| New.CleanUp.ReferenceOTU79629 | 0 | 1 | 0 | 1 | 0 | 1 | 0 |
| New.CleanUp.ReferenceOTU80529 | 0 | 1 | 0 | 1 | 0 | 0 | 0 |
| KC771187.1.1789 | 0 | 1 | 1 | 1 | 1 | 1 | 1 |
| GQ246179.1.1760 | 0 | 1 | 1 | 1 | 1 | 1 | 1 |
| New.CleanUp.ReferenceOTU58954 | 1 | 1 | 1 | 1 | 1 | 1 | 0 |
| CEOE01007823.4128.5910 | 1 | 1 | 1 | 1 | 1 | 1 | 0 |
| New.CleanUp.ReferenceOTU18312 | 0 | 1 | 0 | 1 | 0 | 0 | 0 |
| New.CleanUp.ReferenceOTU23572 | 0 | 0 | 0 | 0 | 1 | 0 | 0 |
| KC583027.1.1771 | 0 | 1 | 1 | 1 | 1 | 1 | 1 |
| DQ647519.1.1771 | 0 | 0 | 1 | 0 | 0 | 1 | 1 |
| KC582999.1.2114 | 1 | 1 | 1 | 1 | 1 | 1 | 1 |
| New.ReferenceOTU254 | 0 | 1 | 1 | 1 | 1 | 1 | 1 |
| New.ReferenceOTU106 | 1 | 1 | 1 | 1 | 1 | 1 | 1 |
| New.ReferenceOTU230 | 1 | 1 | 1 | 1 | 1 | 1 | 1 |
| New.ReferenceOTU311 | 1 | 1 | 1 | 1 | 1 | 1 | 1 |
| New.CleanUp.ReferenceOTU2513 | 0 | 1 | 0 | 1 | 1 | 1 | 1 |
| New.CleanUp.ReferenceOTU2788 | 0 | 1 | 0 | 1 | 0 | 1 | 1 |
| New.CleanUp.ReferenceOTU4142 | 0 | 0 | 0 | 0 | 0 | 1 | 1 |
| New.CleanUp.ReferenceOTU4372 | 0 | 1 | 0 | 1 | 0 | 1 | 1 |
| New.CleanUp.ReferenceOTU4434 | 0 | 1 | 0 | 1 | 0 | 1 | 1 |
| New.CleanUp.ReferenceOTU6093 | 0 | 0 | 0 | 1 | 1 | 0 | 1 |
| New.CleanUp.ReferenceOTU7791 | 0 | 0 | 0 | 1 | 0 | 1 | 1 |
| New.CleanUp.ReferenceOTU7850 | 0 | 1 | 0 | 1 | 1 | 1 | 1 |
| New.CleanUp.ReferenceOTU8155 | 0 | 1 | 0 | 1 | 0 | 0 | 0 |
| New.CleanUp.ReferenceOTU8600 | 0 | 0 | 0 | 0 | 0 | 1 | 1 |
| New.CleanUp.ReferenceOTU10243 | 0 | 1 | 0 | 1 | 1 | 1 | 1 |
| New.CleanUp.ReferenceOTU10296 | 0 | 0 | 0 | 0 | 0 | 1 | 1 |
| New.CleanUp.ReferenceOTU10431 | 0 | 1 | 1 | 1 | 1 | 1 | 1 |
| New.CleanUp.ReferenceOTU11117 | 0 | 0 | 0 | 0 | 1 | 0 | 1 |
| New.CleanUp.ReferenceOTU11354 | 0 | 1 | 1 | 1 | 0 | 1 | 0 |
| New.CleanUp.ReferenceOTU11395 | 0 | 0 | 1 | 0 | 0 | 0 | 1 |
| New.CleanUp.ReferenceOTU12184 | 0 | 0 | 0 | 1 | 0 | 1 | 1 |
| New.CleanUp.ReferenceOTU12946 | 0 | 1 | 0 | 0 | 0 | 1 | 1 |
| New.CleanUp.ReferenceOTU13239 | 0 | 1 | 0 | 0 | 0 | 1 | 1 |
| New.CleanUp.ReferenceOTU14118 | 0 | 0 | 0 | 1 | 0 | 0 | 1 |
| New.CleanUp.ReferenceOTU15467 | 0 | 0 | 0 | 1 | 0 | 1 | 1 |
| New.CleanUp.ReferenceOTU16114 | 0 | 0 | 0 | 1 | 1 | 0 | 1 |
| New.CleanUp.ReferenceOTU16575 | 0 | 0 | 0 | 1 | 0 | 1 | 0 |
| New.CleanUp.ReferenceOTU17074 | 0 | 1 | 0 | 1 | 0 | 0 | 0 |
| New.CleanUp.ReferenceOTU22772 | 0 | 0 | 1 | 0 | 0 | 1 | 1 |
| New.CleanUp.ReferenceOTU25117 | 0 | 0 | 1 | 0 | 0 | 0 | 1 |
| New.CleanUp.ReferenceOTU25200 | 0 | 1 | 0 | 0 | 0 | 1 | 1 |
| New.CleanUp.ReferenceOTU25484 | 0 | 1 | 1 | 0 | 1 | 0 | 0 |
| New.CleanUp.ReferenceOTU25697 | 0 | 1 | 0 | 1 | 0 | 1 | 1 |
| New.CleanUp.ReferenceOTU26085 | 0 | 0 | 0 | 1 | 1 | 0 | 1 |
| New.CleanUp.ReferenceOTU26815 | 0 | 1 | 0 | 0 | 0 | 1 | 0 |
| New.CleanUp.ReferenceOTU28324 | 0 | 0 | 0 | 0 | 0 | 0 | 1 |
| New.CleanUp.ReferenceOTU29711 | 0 | 1 | 0 | 0 | 0 | 1 | 1 |
| New.CleanUp.ReferenceOTU30510 | 0 | 1 | 0 | 1 | 1 | 1 | 1 |
| New.CleanUp.ReferenceOTU31027 | 0 | 1 | 0 | 0 | 0 | 1 | 1 |
| New.CleanUp.ReferenceOTU31485 | 0 | 1 | 1 | 1 | 1 | 1 | 1 |
| New.CleanUp.ReferenceOTU32842 | 0 | 0 | 1 | 0 | 1 | 0 | 0 |
| New.CleanUp.ReferenceOTU33278 | 0 | 1 | 0 | 1 | 1 | 1 | 1 |
| New.CleanUp.ReferenceOTU33537 | 0 | 1 | 0 | 1 | 0 | 0 | 1 |
| New.CleanUp.ReferenceOTU33615 | 0 | 0 | 1 | 0 | 0 | 1 | 1 |
| New.CleanUp.ReferenceOTU34625 | 0 | 1 | 0 | 0 | 0 | 1 | 1 |
| New.CleanUp.ReferenceOTU35297 | 0 | 0 | 0 | 1 | 1 | 1 | 1 |
| New.CleanUp.ReferenceOTU35305 | 0 | 0 | 1 | 0 | 0 | 1 | 1 |
| New.CleanUp.ReferenceOTU35681 | 0 | 0 | 0 | 0 | 0 | 1 | 1 |
| New.CleanUp.ReferenceOTU36594 | 0 | 1 | 0 | 0 | 0 | 1 | 1 |
| New.CleanUp.ReferenceOTU37216 | 0 | 1 | 0 | 0 | 0 | 1 | 1 |
| New.CleanUp.ReferenceOTU37581 | 0 | 0 | 0 | 1 | 0 | 1 | 1 |
| New.CleanUp.ReferenceOTU39706 | 0 | 1 | 0 | 1 | 1 | 1 | 1 |
| New.CleanUp.ReferenceOTU41258 | 0 | 1 | 0 | 1 | 0 | 1 | 1 |
| New.CleanUp.ReferenceOTU41445 | 0 | 0 | 1 | 1 | 1 | 1 | 0 |
| New.CleanUp.ReferenceOTU42351 | 0 | 0 | 0 | 1 | 0 | 1 | 1 |
| New.CleanUp.ReferenceOTU43953 | 0 | 0 | 0 | 1 | 0 | 1 | 1 |
| New.CleanUp.ReferenceOTU44394 | 0 | 1 | 0 | 1 | 0 | 0 | 1 |
| New.CleanUp.ReferenceOTU46420 | 0 | 1 | 0 | 1 | 1 | 1 | 1 |
| New.CleanUp.ReferenceOTU46859 | 0 | 0 | 0 | 1 | 0 | 0 | 1 |
| New.CleanUp.ReferenceOTU47471 | 0 | 1 | 0 | 0 | 0 | 1 | 0 |
| New.CleanUp.ReferenceOTU47855 | 0 | 0 | 1 | 1 | 1 | 1 | 0 |
| New.CleanUp.ReferenceOTU48473 | 0 | 1 | 0 | 1 | 0 | 1 | 1 |
| New.CleanUp.ReferenceOTU48640 | 0 | 1 | 1 | 1 | 1 | 1 | 1 |
| New.CleanUp.ReferenceOTU48707 | 0 | 1 | 0 | 1 | 0 | 0 | 1 |
| New.CleanUp.ReferenceOTU50246 | 0 | 1 | 1 | 1 | 0 | 1 | 1 |
| New.CleanUp.ReferenceOTU51555 | 0 | 1 | 0 | 0 | 0 | 1 | 1 |
| New.CleanUp.ReferenceOTU51766 | 0 | 0 | 1 | 0 | 0 | 1 | 1 |
| New.CleanUp.ReferenceOTU54768 | 0 | 0 | 0 | 1 | 0 | 1 | 1 |
| New.CleanUp.ReferenceOTU54821 | 0 | 0 | 0 | 1 | 0 | 0 | 1 |
| New.CleanUp.ReferenceOTU57166 | 0 | 1 | 0 | 1 | 0 | 1 | 1 |
| New.CleanUp.ReferenceOTU57389 | 0 | 1 | 1 | 1 | 0 | 0 | 1 |
| New.CleanUp.ReferenceOTU57719 | 0 | 0 | 1 | 1 | 1 | 1 | 1 |
| New.CleanUp.ReferenceOTU59966 | 0 | 0 | 0 | 1 | 0 | 1 | 0 |
| New.CleanUp.ReferenceOTU60543 | 0 | 1 | 0 | 1 | 0 | 1 | 1 |
| New.CleanUp.ReferenceOTU62004 | 0 | 0 | 0 | 1 | 0 | 0 | 1 |
| New.CleanUp.ReferenceOTU65190 | 0 | 0 | 1 | 0 | 1 | 1 | 0 |
| New.CleanUp.ReferenceOTU65266 | 0 | 1 | 0 | 1 | 0 | 0 | 0 |
| New.CleanUp.ReferenceOTU68195 | 0 | 0 | 1 | 1 | 1 | 0 | 1 |
| New.CleanUp.ReferenceOTU69694 | 0 | 1 | 0 | 0 | 1 | 0 | 0 |
| New.CleanUp.ReferenceOTU71085 | 0 | 1 | 0 | 0 | 0 | 0 | 1 |
| New.CleanUp.ReferenceOTU76006 | 0 | 0 | 0 | 0 | 0 | 1 | 1 |
| New.CleanUp.ReferenceOTU76833 | 0 | 0 | 0 | 1 | 0 | 1 | 1 |
| New.CleanUp.ReferenceOTU78227 | 0 | 0 | 0 | 1 | 0 | 0 | 0 |
| New.CleanUp.ReferenceOTU79321 | 0 | 1 | 0 | 1 | 0 | 1 | 0 |
| New.CleanUp.ReferenceOTU79389 | 0 | 1 | 0 | 1 | 1 | 1 | 1 |
| New.CleanUp.ReferenceOTU79880 | 0 | 0 | 0 | 0 | 0 | 0 | 1 |
| New.CleanUp.ReferenceOTU80050 | 0 | 1 | 0 | 0 | 0 | 1 | 0 |
| New.CleanUp.ReferenceOTU81175 | 0 | 1 | 0 | 0 | 0 | 1 | 1 |
| New.ReferenceOTU352 | 1 | 1 | 1 | 1 | 1 | 1 | 1 |
| New.CleanUp.ReferenceOTU906 | 0 | 1 | 1 | 0 | 0 | 0 | 0 |
| New.CleanUp.ReferenceOTU3274 | 0 | 0 | 0 | 0 | 0 | 1 | 1 |
| New.CleanUp.ReferenceOTU8976 | 0 | 0 | 0 | 1 | 0 | 0 | 0 |
| New.CleanUp.ReferenceOTU9908 | 0 | 1 | 0 | 1 | 0 | 1 | 0 |
| New.CleanUp.ReferenceOTU14747 | 0 | 1 | 1 | 1 | 0 | 1 | 0 |
| New.CleanUp.ReferenceOTU22316 | 1 | 1 | 1 | 1 | 1 | 1 | 1 |
| New.CleanUp.ReferenceOTU40036 | 0 | 1 | 0 | 1 | 0 | 1 | 1 |
| New.CleanUp.ReferenceOTU41084 | 0 | 1 | 0 | 1 | 0 | 1 | 1 |
| New.CleanUp.ReferenceOTU41941 | 0 | 0 | 0 | 0 | 0 | 1 | 0 |
| New.CleanUp.ReferenceOTU42682 | 0 | 1 | 0 | 0 | 0 | 1 | 0 |
| New.CleanUp.ReferenceOTU58312 | 0 | 1 | 0 | 1 | 0 | 1 | 0 |
| New.CleanUp.ReferenceOTU61011 | 0 | 1 | 1 | 1 | 0 | 1 | 1 |
| New.CleanUp.ReferenceOTU62854 | 0 | 1 | 0 | 1 | 1 | 0 | 0 |
| New.CleanUp.ReferenceOTU80735 | 0 | 1 | 0 | 0 | 0 | 0 | 0 |
| KP404840.1.1682 | 0 | 0 | 1 | 1 | 0 | 1 | 1 |
| New.CleanUp.ReferenceOTU9611 | 1 | 1 | 1 | 1 | 0 | 1 | 1 |
| New.CleanUp.ReferenceOTU25538 | 0 | 0 | 1 | 0 | 0 | 1 | 1 |
| New.CleanUp.ReferenceOTU45902 | 0 | 0 | 0 | 1 | 1 | 0 | 1 |
| New.CleanUp.ReferenceOTU50940 | 0 | 0 | 0 | 0 | 0 | 1 | 1 |
| New.CleanUp.ReferenceOTU76985 | 1 | 1 | 1 | 1 | 0 | 1 | 1 |
| KC583023.1.1782 | 0 | 1 | 1 | 0 | 1 | 1 | 0 |
| KJ763708.1.1799 | 0 | 0 | 0 | 1 | 1 | 1 | 1 |
| New.CleanUp.ReferenceOTU1598 | 0 | 0 | 0 | 0 | 0 | 1 | 1 |
| New.CleanUp.ReferenceOTU2771 | 0 | 0 | 0 | 1 | 1 | 0 | 1 |
| New.CleanUp.ReferenceOTU3105 | 0 | 1 | 0 | 1 | 0 | 0 | 1 |
| New.CleanUp.ReferenceOTU3766 | 0 | 0 | 0 | 0 | 0 | 1 | 1 |
| New.CleanUp.ReferenceOTU5900 | 0 | 0 | 0 | 1 | 0 | 1 | 1 |
| New.CleanUp.ReferenceOTU8662 | 0 | 0 | 1 | 0 | 0 | 1 | 1 |
| New.CleanUp.ReferenceOTU11564 | 0 | 0 | 1 | 0 | 0 | 0 | 0 |
| New.CleanUp.ReferenceOTU11609 | 0 | 0 | 1 | 0 | 0 | 1 | 0 |
| New.CleanUp.ReferenceOTU15546 | 0 | 0 | 0 | 0 | 1 | 0 | 0 |
| New.CleanUp.ReferenceOTU16327 | 0 | 0 | 1 | 0 | 0 | 0 | 1 |
| New.CleanUp.ReferenceOTU19380 | 0 | 0 | 1 | 0 | 1 | 1 | 1 |
| New.CleanUp.ReferenceOTU20194 | 0 | 0 | 0 | 1 | 1 | 1 | 0 |
| New.CleanUp.ReferenceOTU21438 | 0 | 0 | 0 | 1 | 0 | 0 | 1 |
| New.CleanUp.ReferenceOTU23938 | 0 | 1 | 0 | 0 | 1 | 1 | 1 |
| New.CleanUp.ReferenceOTU25102 | 0 | 0 | 1 | 0 | 0 | 1 | 1 |
| New.CleanUp.ReferenceOTU26263 | 0 | 1 | 0 | 0 | 0 | 1 | 1 |
| New.CleanUp.ReferenceOTU28800 | 0 | 0 | 0 | 0 | 0 | 1 | 1 |
| New.CleanUp.ReferenceOTU29080 | 0 | 0 | 0 | 0 | 1 | 1 | 1 |
| New.CleanUp.ReferenceOTU31950 | 0 | 1 | 0 | 0 | 1 | 1 | 1 |
| New.CleanUp.ReferenceOTU32926 | 0 | 0 | 0 | 0 | 0 | 0 | 1 |
| New.CleanUp.ReferenceOTU33553 | 0 | 1 | 0 | 0 | 0 | 0 | 0 |
| New.CleanUp.ReferenceOTU41250 | 0 | 0 | 0 | 0 | 1 | 1 | 1 |
| New.CleanUp.ReferenceOTU46423 | 0 | 1 | 1 | 1 | 1 | 1 | 1 |
| New.CleanUp.ReferenceOTU46824 | 0 | 1 | 0 | 1 | 0 | 1 | 1 |
| New.CleanUp.ReferenceOTU47158 | 0 | 0 | 1 | 1 | 0 | 1 | 0 |
| New.CleanUp.ReferenceOTU48717 | 0 | 0 | 1 | 0 | 0 | 1 | 1 |
| New.CleanUp.ReferenceOTU52068 | 0 | 1 | 0 | 0 | 1 | 0 | 1 |
| New.CleanUp.ReferenceOTU52891 | 0 | 1 | 0 | 1 | 0 | 1 | 1 |
| New.CleanUp.ReferenceOTU56344 | 0 | 1 | 1 | 0 | 1 | 0 | 0 |
| New.CleanUp.ReferenceOTU57037 | 0 | 1 | 0 | 1 | 0 | 1 | 1 |
| New.CleanUp.ReferenceOTU61720 | 0 | 0 | 0 | 1 | 1 | 1 | 1 |
| New.CleanUp.ReferenceOTU62176 | 0 | 0 | 1 | 1 | 0 | 0 | 0 |
| New.CleanUp.ReferenceOTU65955 | 0 | 0 | 1 | 0 | 1 | 0 | 1 |
| New.CleanUp.ReferenceOTU66126 | 0 | 0 | 0 | 1 | 0 | 1 | 0 |
| New.CleanUp.ReferenceOTU66617 | 0 | 0 | 1 | 0 | 1 | 1 | 1 |
| New.CleanUp.ReferenceOTU69254 | 0 | 0 | 0 | 0 | 0 | 1 | 0 |
| New.CleanUp.ReferenceOTU70337 | 0 | 1 | 0 | 0 | 0 | 1 | 1 |
| New.CleanUp.ReferenceOTU75155 | 0 | 0 | 0 | 0 | 0 | 0 | 1 |
| New.CleanUp.ReferenceOTU75176 | 0 | 1 | 1 | 0 | 0 | 0 | 1 |
| New.CleanUp.ReferenceOTU75445 | 0 | 1 | 1 | 1 | 0 | 0 | 1 |
| New.CleanUp.ReferenceOTU76733 | 0 | 0 | 1 | 0 | 0 | 1 | 1 |
| New.CleanUp.ReferenceOTU78079 | 0 | 0 | 0 | 1 | 0 | 0 | 0 |
| New.CleanUp.ReferenceOTU78897 | 0 | 0 | 1 | 0 | 0 | 1 | 1 |
| New.CleanUp.ReferenceOTU79183 | 0 | 0 | 0 | 1 | 1 | 1 | 0 |
| New.CleanUp.ReferenceOTU80506 | 0 | 0 | 1 | 0 | 1 | 1 | 0 |
| FR874389.1.1786 | 0 | 1 | 1 | 1 | 1 | 1 | 1 |
| KJ762940.1.1778 | 0 | 0 | 1 | 0 | 0 | 0 | 0 |
| KF130338.1.1751 | 1 | 1 | 1 | 1 | 1 | 1 | 1 |
| FR874767.1.1785 | 1 | 1 | 1 | 1 | 1 | 1 | 1 |
| FR874359.1.1783 | 1 | 1 | 1 | 1 | 1 | 1 | 1 |
| New.ReferenceOTU323 | 1 | 1 | 1 | 1 | 1 | 1 | 1 |
| New.ReferenceOTU112 | 0 | 1 | 1 | 1 | 0 | 1 | 1 |
| New.ReferenceOTU266 | 1 | 1 | 1 | 1 | 1 | 1 | 1 |
| New.CleanUp.ReferenceOTU205 | 1 | 1 | 1 | 1 | 1 | 1 | 1 |
| New.CleanUp.ReferenceOTU457 | 0 | 1 | 0 | 0 | 1 | 1 | 0 |
| New.CleanUp.ReferenceOTU508 | 0 | 0 | 1 | 0 | 0 | 1 | 0 |
| New.CleanUp.ReferenceOTU676 | 0 | 0 | 0 | 1 | 0 | 0 | 1 |
| New.CleanUp.ReferenceOTU835 | 0 | 0 | 0 | 1 | 0 | 1 | 1 |
| New.CleanUp.ReferenceOTU1045 | 0 | 1 | 1 | 1 | 1 | 1 | 1 |
| New.CleanUp.ReferenceOTU1313 | 1 | 1 | 1 | 1 | 1 | 1 | 1 |
| New.CleanUp.ReferenceOTU1788 | 0 | 1 | 1 | 1 | 0 | 1 | 1 |
| New.CleanUp.ReferenceOTU2766 | 0 | 0 | 1 | 1 | 1 | 1 | 1 |
| New.CleanUp.ReferenceOTU5484 | 0 | 1 | 1 | 1 | 1 | 1 | 1 |
| New.CleanUp.ReferenceOTU5509 | 1 | 1 | 1 | 0 | 0 | 0 | 0 |
| New.CleanUp.ReferenceOTU6686 | 0 | 0 | 0 | 0 | 0 | 0 | 1 |
| New.CleanUp.ReferenceOTU6981 | 1 | 1 | 0 | 1 | 1 | 1 | 1 |
| New.CleanUp.ReferenceOTU7233 | 0 | 1 | 0 | 1 | 1 | 0 | 0 |
| New.CleanUp.ReferenceOTU7659 | 0 | 0 | 1 | 1 | 1 | 1 | 1 |
| New.CleanUp.ReferenceOTU9357 | 0 | 1 | 1 | 1 | 1 | 1 | 1 |
| New.CleanUp.ReferenceOTU10486 | 0 | 1 | 0 | 1 | 0 | 0 | 0 |
| New.CleanUp.ReferenceOTU11371 | 1 | 1 | 0 | 0 | 0 | 1 | 0 |
| New.CleanUp.ReferenceOTU12178 | 1 | 1 | 1 | 1 | 1 | 1 | 1 |
| New.CleanUp.ReferenceOTU13144 | 0 | 0 | 0 | 0 | 1 | 1 | 1 |
| New.CleanUp.ReferenceOTU13902 | 0 | 0 | 1 | 1 | 0 | 1 | 0 |
| New.CleanUp.ReferenceOTU14604 | 0 | 0 | 1 | 0 | 1 | 1 | 1 |
| New.CleanUp.ReferenceOTU15669 | 1 | 1 | 0 | 0 | 1 | 1 | 1 |
| New.CleanUp.ReferenceOTU15774 | 0 | 0 | 0 | 0 | 0 | 0 | 1 |
| New.CleanUp.ReferenceOTU15914 | 0 | 1 | 1 | 1 | 1 | 1 | 1 |
| New.CleanUp.ReferenceOTU17040 | 0 | 0 | 0 | 0 | 1 | 1 | 1 |
| New.CleanUp.ReferenceOTU17690 | 0 | 0 | 0 | 0 | 0 | 1 | 1 |
| New.CleanUp.ReferenceOTU17997 | 0 | 0 | 1 | 0 | 1 | 1 | 1 |
| New.CleanUp.ReferenceOTU19002 | 1 | 0 | 0 | 0 | 0 | 1 | 1 |
| New.CleanUp.ReferenceOTU20384 | 0 | 0 | 0 | 0 | 1 | 0 | 1 |
| New.CleanUp.ReferenceOTU21128 | 0 | 1 | 1 | 0 | 0 | 1 | 0 |
| New.CleanUp.ReferenceOTU21535 | 0 | 1 | 1 | 1 | 1 | 1 | 1 |
| New.CleanUp.ReferenceOTU21777 | 0 | 0 | 0 | 1 | 0 | 1 | 1 |
| New.CleanUp.ReferenceOTU22380 | 0 | 1 | 1 | 1 | 0 | 0 | 1 |
| New.CleanUp.ReferenceOTU22794 | 0 | 1 | 1 | 1 | 0 | 1 | 1 |
| New.CleanUp.ReferenceOTU22874 | 0 | 0 | 1 | 0 | 0 | 0 | 1 |
| New.CleanUp.ReferenceOTU23081 | 1 | 0 | 1 | 1 | 1 | 1 | 1 |
| New.CleanUp.ReferenceOTU23432 | 0 | 1 | 0 | 0 | 0 | 0 | 1 |
| New.CleanUp.ReferenceOTU25164 | 0 | 0 | 0 | 1 | 0 | 1 | 0 |
| New.CleanUp.ReferenceOTU25360 | 0 | 1 | 1 | 0 | 0 | 1 | 0 |
| New.CleanUp.ReferenceOTU26556 | 1 | 0 | 0 | 1 | 1 | 1 | 1 |
| New.CleanUp.ReferenceOTU26943 | 0 | 1 | 1 | 0 | 1 | 1 | 0 |
| New.CleanUp.ReferenceOTU27057 | 0 | 0 | 1 | 0 | 0 | 1 | 1 |
| New.CleanUp.ReferenceOTU28322 | 1 | 0 | 0 | 1 | 0 | 1 | 1 |
| New.CleanUp.ReferenceOTU29997 | 0 | 0 | 1 | 0 | 0 | 1 | 1 |
| New.CleanUp.ReferenceOTU30016 | 0 | 1 | 1 | 1 | 1 | 1 | 1 |
| New.CleanUp.ReferenceOTU32174 | 0 | 0 | 0 | 0 | 0 | 1 | 1 |
| New.CleanUp.ReferenceOTU32935 | 0 | 0 | 1 | 0 | 0 | 0 | 0 |
| New.CleanUp.ReferenceOTU33421 | 0 | 1 | 1 | 0 | 1 | 1 | 1 |
| New.CleanUp.ReferenceOTU34335 | 0 | 1 | 1 | 1 | 0 | 0 | 0 |
| New.CleanUp.ReferenceOTU35171 | 0 | 0 | 0 | 1 | 1 | 1 | 0 |
| New.CleanUp.ReferenceOTU36822 | 0 | 0 | 0 | 1 | 0 | 1 | 0 |
| New.CleanUp.ReferenceOTU37269 | 0 | 0 | 1 | 1 | 0 | 1 | 1 |
| New.CleanUp.ReferenceOTU38653 | 0 | 0 | 1 | 1 | 0 | 0 | 1 |
| New.CleanUp.ReferenceOTU38822 | 0 | 0 | 0 | 1 | 0 | 1 | 1 |
| New.CleanUp.ReferenceOTU38834 | 0 | 1 | 0 | 0 | 1 | 0 | 1 |
| New.CleanUp.ReferenceOTU39863 | 1 | 0 | 0 | 0 | 0 | 0 | 0 |
| New.CleanUp.ReferenceOTU40447 | 0 | 1 | 0 | 1 | 1 | 1 | 1 |
| New.CleanUp.ReferenceOTU40876 | 0 | 0 | 1 | 1 | 1 | 1 | 1 |
| New.CleanUp.ReferenceOTU42357 | 0 | 1 | 0 | 1 | 1 | 0 | 1 |
| New.CleanUp.ReferenceOTU42940 | 0 | 1 | 1 | 1 | 1 | 1 | 1 |
| New.CleanUp.ReferenceOTU46996 | 0 | 1 | 0 | 0 | 0 | 0 | 1 |
| New.CleanUp.ReferenceOTU48964 | 0 | 1 | 0 | 1 | 0 | 0 | 0 |
| New.CleanUp.ReferenceOTU51334 | 0 | 0 | 1 | 1 | 1 | 1 | 1 |
| New.CleanUp.ReferenceOTU51910 | 1 | 1 | 0 | 0 | 0 | 1 | 0 |
| New.CleanUp.ReferenceOTU52041 | 1 | 0 | 1 | 0 | 0 | 1 | 0 |
| New.CleanUp.ReferenceOTU53008 | 0 | 0 | 0 | 0 | 0 | 1 | 1 |
| New.CleanUp.ReferenceOTU53333 | 0 | 0 | 1 | 0 | 1 | 0 | 0 |
| New.CleanUp.ReferenceOTU53638 | 0 | 0 | 0 | 1 | 0 | 1 | 1 |
| New.CleanUp.ReferenceOTU53882 | 0 | 0 | 0 | 0 | 0 | 0 | 1 |
| New.CleanUp.ReferenceOTU54517 | 1 | 0 | 0 | 0 | 1 | 1 | 0 |
| New.CleanUp.ReferenceOTU56107 | 0 | 1 | 1 | 1 | 0 | 0 | 0 |
| New.CleanUp.ReferenceOTU56145 | 0 | 1 | 0 | 0 | 0 | 0 | 1 |
| New.CleanUp.ReferenceOTU56521 | 0 | 0 | 1 | 1 | 1 | 0 | 0 |
| New.CleanUp.ReferenceOTU56736 | 0 | 0 | 0 | 1 | 0 | 0 | 1 |
| New.CleanUp.ReferenceOTU56983 | 0 | 1 | 0 | 0 | 0 | 1 | 0 |
| New.CleanUp.ReferenceOTU57128 | 0 | 1 | 1 | 1 | 1 | 1 | 1 |
| New.CleanUp.ReferenceOTU57498 | 0 | 1 | 1 | 1 | 0 | 1 | 1 |
| New.CleanUp.ReferenceOTU58238 | 0 | 0 | 1 | 1 | 0 | 1 | 0 |
| New.CleanUp.ReferenceOTU58550 | 0 | 1 | 0 | 1 | 1 | 1 | 1 |
| New.CleanUp.ReferenceOTU58972 | 0 | 1 | 0 | 1 | 0 | 1 | 0 |
| New.CleanUp.ReferenceOTU59534 | 0 | 1 | 1 | 1 | 1 | 1 | 1 |
| New.CleanUp.ReferenceOTU59995 | 0 | 1 | 0 | 1 | 0 | 0 | 0 |
| New.CleanUp.ReferenceOTU60924 | 0 | 0 | 0 | 1 | 0 | 1 | 1 |
| New.CleanUp.ReferenceOTU61492 | 0 | 1 | 1 | 1 | 1 | 1 | 1 |
| New.CleanUp.ReferenceOTU62147 | 0 | 0 | 0 | 1 | 0 | 1 | 0 |
| New.CleanUp.ReferenceOTU62194 | 0 | 1 | 1 | 1 | 1 | 1 | 0 |
| New.CleanUp.ReferenceOTU62201 | 0 | 0 | 1 | 0 | 1 | 0 | 1 |
| New.CleanUp.ReferenceOTU62219 | 0 | 0 | 1 | 0 | 0 | 0 | 1 |
| New.CleanUp.ReferenceOTU62643 | 0 | 0 | 0 | 0 | 0 | 1 | 1 |
| New.CleanUp.ReferenceOTU63535 | 0 | 0 | 0 | 0 | 0 | 0 | 1 |
| New.CleanUp.ReferenceOTU63700 | 0 | 0 | 1 | 1 | 1 | 0 | 0 |
| New.CleanUp.ReferenceOTU65198 | 0 | 0 | 1 | 0 | 1 | 1 | 1 |
| New.CleanUp.ReferenceOTU65380 | 0 | 0 | 0 | 1 | 1 | 1 | 1 |
| New.CleanUp.ReferenceOTU65980 | 0 | 1 | 0 | 0 | 0 | 1 | 1 |
| New.CleanUp.ReferenceOTU66481 | 0 | 0 | 0 | 0 | 0 | 1 | 1 |
| New.CleanUp.ReferenceOTU67325 | 0 | 0 | 0 | 0 | 0 | 0 | 1 |
| New.CleanUp.ReferenceOTU67463 | 1 | 0 | 1 | 0 | 0 | 1 | 1 |
| New.CleanUp.ReferenceOTU67999 | 0 | 1 | 1 | 1 | 1 | 1 | 1 |
| New.CleanUp.ReferenceOTU68375 | 0 | 0 | 0 | 1 | 0 | 1 | 1 |
| New.CleanUp.ReferenceOTU69083 | 0 | 1 | 0 | 1 | 0 | 1 | 1 |
| New.CleanUp.ReferenceOTU69125 | 1 | 1 | 1 | 1 | 0 | 1 | 1 |
| New.CleanUp.ReferenceOTU70415 | 0 | 1 | 1 | 0 | 1 | 0 | 0 |
| New.CleanUp.ReferenceOTU71564 | 0 | 1 | 0 | 0 | 1 | 0 | 1 |
| New.CleanUp.ReferenceOTU71641 | 0 | 1 | 1 | 1 | 1 | 1 | 1 |
| New.CleanUp.ReferenceOTU72073 | 0 | 0 | 0 | 0 | 0 | 1 | 1 |
| New.CleanUp.ReferenceOTU72096 | 1 | 1 | 0 | 0 | 0 | 0 | 1 |
| New.CleanUp.ReferenceOTU75242 | 1 | 1 | 1 | 1 | 0 | 1 | 1 |
| New.CleanUp.ReferenceOTU76694 | 0 | 1 | 0 | 0 | 0 | 1 | 1 |
| New.CleanUp.ReferenceOTU76715 | 1 | 1 | 1 | 1 | 1 | 1 | 1 |
| New.CleanUp.ReferenceOTU77919 | 0 | 1 | 0 | 0 | 1 | 1 | 1 |
| New.CleanUp.ReferenceOTU79810 | 0 | 0 | 1 | 1 | 1 | 1 | 1 |
| New.CleanUp.ReferenceOTU80787 | 1 | 1 | 0 | 0 | 1 | 1 | 1 |
| New.CleanUp.ReferenceOTU80973 | 0 | 1 | 0 | 0 | 0 | 1 | 1 |
| KJ763237.1.1781 | 0 | 1 | 1 | 1 | 1 | 0 | 0 |
| KF129987.1.1743 | 1 | 1 | 1 | 1 | 1 | 1 | 1 |
| New.ReferenceOTU274 | 1 | 1 | 1 | 1 | 1 | 1 | 1 |
| New.ReferenceOTU277 | 0 | 1 | 1 | 1 | 1 | 1 | 1 |
| New.ReferenceOTU359 | 1 | 1 | 1 | 1 | 1 | 1 | 1 |
| New.ReferenceOTU84 | 1 | 1 | 1 | 1 | 1 | 1 | 1 |
| New.CleanUp.ReferenceOTU534 | 0 | 0 | 0 | 0 | 1 | 0 | 1 |
| New.CleanUp.ReferenceOTU1157 | 0 | 0 | 0 | 0 | 1 | 1 | 1 |
| New.CleanUp.ReferenceOTU1469 | 0 | 0 | 0 | 1 | 0 | 1 | 1 |
| New.CleanUp.ReferenceOTU1903 | 0 | 1 | 1 | 1 | 1 | 1 | 1 |
| New.CleanUp.ReferenceOTU2305 | 0 | 0 | 0 | 0 | 0 | 1 | 0 |
| New.CleanUp.ReferenceOTU2650 | 1 | 0 | 1 | 1 | 1 | 1 | 1 |
| New.CleanUp.ReferenceOTU3338 | 0 | 1 | 1 | 1 | 0 | 0 | 0 |
| New.CleanUp.ReferenceOTU3790 | 0 | 0 | 0 | 1 | 0 | 0 | 0 |
| New.CleanUp.ReferenceOTU4193 | 0 | 0 | 0 | 0 | 1 | 0 | 0 |
| New.CleanUp.ReferenceOTU4397 | 0 | 0 | 1 | 0 | 0 | 0 | 0 |
| New.CleanUp.ReferenceOTU4736 | 0 | 1 | 1 | 1 | 0 | 0 | 0 |
| New.CleanUp.ReferenceOTU5325 | 0 | 1 | 0 | 1 | 0 | 1 | 1 |
| New.CleanUp.ReferenceOTU5720 | 0 | 1 | 0 | 1 | 0 | 0 | 1 |
| New.CleanUp.ReferenceOTU5724 | 1 | 0 | 0 | 0 | 0 | 0 | 0 |
| New.CleanUp.ReferenceOTU7686 | 0 | 0 | 0 | 0 | 1 | 1 | 0 |
| New.CleanUp.ReferenceOTU7871 | 0 | 1 | 1 | 1 | 0 | 1 | 1 |
| New.CleanUp.ReferenceOTU9370 | 0 | 1 | 0 | 1 | 0 | 1 | 0 |
| New.CleanUp.ReferenceOTU9440 | 0 | 1 | 0 | 0 | 1 | 1 | 1 |
| New.CleanUp.ReferenceOTU10515 | 0 | 1 | 0 | 1 | 0 | 0 | 0 |
| New.CleanUp.ReferenceOTU11531 | 1 | 1 | 0 | 1 | 0 | 0 | 0 |
| New.CleanUp.ReferenceOTU12159 | 0 | 0 | 0 | 1 | 0 | 0 | 1 |
| New.CleanUp.ReferenceOTU13772 | 0 | 0 | 0 | 1 | 0 | 1 | 1 |
| New.CleanUp.ReferenceOTU14302 | 0 | 0 | 1 | 1 | 1 | 0 | 0 |
| New.CleanUp.ReferenceOTU15097 | 0 | 0 | 0 | 0 | 1 | 1 | 1 |
| New.CleanUp.ReferenceOTU15518 | 0 | 1 | 0 | 0 | 0 | 1 | 1 |
| New.CleanUp.ReferenceOTU16143 | 0 | 1 | 0 | 0 | 1 | 0 | 0 |
| New.CleanUp.ReferenceOTU16771 | 0 | 0 | 1 | 1 | 0 | 1 | 1 |
| New.CleanUp.ReferenceOTU18396 | 0 | 0 | 0 | 0 | 1 | 0 | 1 |
| New.CleanUp.ReferenceOTU20926 | 0 | 0 | 1 | 0 | 1 | 1 | 0 |
| New.CleanUp.ReferenceOTU21373 | 0 | 1 | 1 | 1 | 1 | 1 | 0 |
| New.CleanUp.ReferenceOTU21778 | 0 | 0 | 1 | 0 | 0 | 1 | 1 |
| New.CleanUp.ReferenceOTU22238 | 0 | 1 | 0 | 0 | 1 | 1 | 0 |
| New.CleanUp.ReferenceOTU22336 | 1 | 0 | 0 | 0 | 0 | 0 | 0 |
| New.CleanUp.ReferenceOTU22424 | 0 | 1 | 0 | 1 | 1 | 1 | 1 |
| New.CleanUp.ReferenceOTU22731 | 0 | 0 | 0 | 1 | 0 | 1 | 1 |
| New.CleanUp.ReferenceOTU22936 | 0 | 0 | 0 | 0 | 0 | 1 | 0 |
| New.CleanUp.ReferenceOTU23737 | 0 | 0 | 0 | 0 | 1 | 1 | 1 |
| New.CleanUp.ReferenceOTU23753 | 0 | 0 | 1 | 0 | 1 | 0 | 0 |
| New.CleanUp.ReferenceOTU25086 | 0 | 0 | 1 | 0 | 0 | 0 | 0 |
| New.CleanUp.ReferenceOTU25687 | 0 | 1 | 1 | 0 | 1 | 1 | 1 |
| New.CleanUp.ReferenceOTU27718 | 0 | 1 | 1 | 1 | 0 | 1 | 1 |
| New.CleanUp.ReferenceOTU28690 | 0 | 0 | 0 | 0 | 1 | 0 | 1 |
| New.CleanUp.ReferenceOTU29972 | 0 | 0 | 0 | 0 | 0 | 1 | 0 |
| New.CleanUp.ReferenceOTU31386 | 0 | 0 | 0 | 1 | 1 | 0 | 0 |
| New.CleanUp.ReferenceOTU31410 | 0 | 0 | 1 | 0 | 0 | 0 | 1 |
| New.CleanUp.ReferenceOTU32526 | 0 | 1 | 0 | 1 | 0 | 1 | 1 |
| New.CleanUp.ReferenceOTU32735 | 0 | 1 | 0 | 0 | 0 | 1 | 1 |
| New.CleanUp.ReferenceOTU34687 | 0 | 0 | 0 | 1 | 0 | 1 | 1 |
| New.CleanUp.ReferenceOTU34928 | 0 | 1 | 1 | 0 | 0 | 0 | 0 |
| New.CleanUp.ReferenceOTU35995 | 1 | 1 | 1 | 1 | 1 | 1 | 1 |
| New.CleanUp.ReferenceOTU36168 | 0 | 1 | 0 | 0 | 0 | 1 | 0 |
| New.CleanUp.ReferenceOTU36547 | 0 | 1 | 1 | 0 | 0 | 0 | 1 |
| New.CleanUp.ReferenceOTU36768 | 0 | 0 | 0 | 1 | 0 | 1 | 1 |
| New.CleanUp.ReferenceOTU37846 | 0 | 1 | 1 | 1 | 1 | 1 | 1 |
| New.CleanUp.ReferenceOTU38025 | 0 | 0 | 0 | 0 | 0 | 0 | 1 |
| New.CleanUp.ReferenceOTU39539 | 0 | 1 | 0 | 0 | 0 | 1 | 1 |
| New.CleanUp.ReferenceOTU40084 | 0 | 0 | 1 | 0 | 0 | 0 | 0 |
| New.CleanUp.ReferenceOTU42804 | 0 | 0 | 1 | 1 | 0 | 0 | 0 |
| New.CleanUp.ReferenceOTU43637 | 0 | 1 | 0 | 1 | 0 | 1 | 0 |
| New.CleanUp.ReferenceOTU44847 | 0 | 1 | 0 | 1 | 0 | 1 | 1 |
| New.CleanUp.ReferenceOTU45521 | 0 | 1 | 0 | 1 | 1 | 1 | 1 |
| New.CleanUp.ReferenceOTU45822 | 0 | 0 | 1 | 1 | 0 | 1 | 0 |
| New.CleanUp.ReferenceOTU46381 | 0 | 1 | 0 | 1 | 0 | 0 | 0 |
| New.CleanUp.ReferenceOTU46896 | 1 | 1 | 1 | 1 | 1 | 1 | 0 |
| New.CleanUp.ReferenceOTU47070 | 0 | 0 | 1 | 0 | 0 | 0 | 1 |
| New.CleanUp.ReferenceOTU47556 | 0 | 0 | 0 | 1 | 0 | 0 | 1 |
| New.CleanUp.ReferenceOTU48587 | 0 | 0 | 1 | 0 | 0 | 0 | 0 |
| New.CleanUp.ReferenceOTU48817 | 0 | 0 | 0 | 0 | 0 | 1 | 1 |
| New.CleanUp.ReferenceOTU48993 | 0 | 0 | 1 | 1 | 1 | 1 | 1 |
| New.CleanUp.ReferenceOTU49246 | 0 | 0 | 0 | 0 | 0 | 1 | 1 |
| New.CleanUp.ReferenceOTU49342 | 0 | 0 | 0 | 1 | 0 | 0 | 1 |
| New.CleanUp.ReferenceOTU49678 | 0 | 1 | 1 | 0 | 0 | 1 | 0 |
| New.CleanUp.ReferenceOTU52693 | 0 | 1 | 0 | 1 | 0 | 0 | 1 |
| New.CleanUp.ReferenceOTU55913 | 1 | 1 | 1 | 1 | 0 | 1 | 1 |
| New.CleanUp.ReferenceOTU56092 | 0 | 1 | 0 | 0 | 1 | 1 | 1 |
| New.CleanUp.ReferenceOTU56126 | 0 | 1 | 0 | 1 | 0 | 1 | 1 |
| New.CleanUp.ReferenceOTU56172 | 0 | 1 | 0 | 0 | 0 | 0 | 1 |
| New.CleanUp.ReferenceOTU56216 | 0 | 0 | 1 | 0 | 0 | 0 | 0 |
| New.CleanUp.ReferenceOTU56539 | 0 | 0 | 0 | 0 | 0 | 1 | 1 |
| New.CleanUp.ReferenceOTU56719 | 1 | 1 | 0 | 0 | 1 | 0 | 0 |
| New.CleanUp.ReferenceOTU58741 | 0 | 0 | 1 | 0 | 0 | 1 | 1 |
| New.CleanUp.ReferenceOTU58869 | 0 | 0 | 0 | 0 | 0 | 1 | 1 |
| New.CleanUp.ReferenceOTU58997 | 0 | 0 | 0 | 1 | 1 | 0 | 1 |
| New.CleanUp.ReferenceOTU59651 | 0 | 1 | 0 | 0 | 0 | 0 | 1 |
| New.CleanUp.ReferenceOTU59986 | 0 | 0 | 0 | 1 | 0 | 0 | 1 |
| New.CleanUp.ReferenceOTU60338 | 0 | 1 | 1 | 0 | 1 | 1 | 1 |
| New.CleanUp.ReferenceOTU60375 | 0 | 0 | 0 | 0 | 1 | 0 | 0 |
| New.CleanUp.ReferenceOTU61012 | 1 | 1 | 1 | 1 | 1 | 1 | 1 |
| New.CleanUp.ReferenceOTU61188 | 0 | 0 | 0 | 0 | 0 | 1 | 1 |
| New.CleanUp.ReferenceOTU61590 | 0 | 0 | 1 | 0 | 0 | 1 | 1 |
| New.CleanUp.ReferenceOTU61617 | 1 | 0 | 0 | 0 | 1 | 1 | 0 |
| New.CleanUp.ReferenceOTU63309 | 0 | 1 | 0 | 1 | 0 | 0 | 1 |
| New.CleanUp.ReferenceOTU63722 | 0 | 1 | 0 | 1 | 0 | 0 | 1 |
| New.CleanUp.ReferenceOTU64319 | 0 | 1 | 0 | 0 | 0 | 1 | 1 |
| New.CleanUp.ReferenceOTU64361 | 0 | 0 | 0 | 0 | 0 | 1 | 0 |
| New.CleanUp.ReferenceOTU64389 | 0 | 1 | 1 | 0 | 0 | 1 | 1 |
| New.CleanUp.ReferenceOTU64807 | 0 | 1 | 1 | 0 | 1 | 1 | 1 |
| New.CleanUp.ReferenceOTU65059 | 0 | 0 | 1 | 0 | 0 | 1 | 1 |
| New.CleanUp.ReferenceOTU65099 | 0 | 1 | 0 | 0 | 0 | 1 | 1 |
| New.CleanUp.ReferenceOTU68243 | 1 | 0 | 0 | 0 | 1 | 1 | 0 |
| New.CleanUp.ReferenceOTU68559 | 0 | 1 | 0 | 1 | 1 | 1 | 1 |
| New.CleanUp.ReferenceOTU72620 | 1 | 1 | 1 | 0 | 0 | 0 | 0 |
| New.CleanUp.ReferenceOTU72810 | 0 | 1 | 1 | 0 | 1 | 0 | 1 |
| New.CleanUp.ReferenceOTU75204 | 0 | 0 | 0 | 1 | 0 | 0 | 0 |
| New.CleanUp.ReferenceOTU76022 | 0 | 0 | 1 | 1 | 0 | 0 | 1 |
| New.CleanUp.ReferenceOTU76055 | 0 | 1 | 0 | 0 | 1 | 1 | 0 |
| New.CleanUp.ReferenceOTU77145 | 0 | 1 | 0 | 1 | 0 | 0 | 0 |
| New.CleanUp.ReferenceOTU79107 | 0 | 1 | 1 | 1 | 0 | 1 | 1 |
| New.CleanUp.ReferenceOTU79323 | 0 | 1 | 0 | 1 | 0 | 0 | 1 |
| New.CleanUp.ReferenceOTU79746 | 0 | 1 | 1 | 1 | 1 | 1 | 1 |
| New.CleanUp.ReferenceOTU79749 | 0 | 0 | 0 | 0 | 0 | 1 | 1 |
| New.CleanUp.ReferenceOTU81323 | 0 | 1 | 0 | 1 | 0 | 1 | 1 |
| New.ReferenceOTU217 | 0 | 1 | 1 | 1 | 1 | 1 | 1 |
| New.CleanUp.ReferenceOTU3143 | 1 | 1 | 1 | 1 | 1 | 1 | 1 |
| New.CleanUp.ReferenceOTU5469 | 0 | 1 | 0 | 1 | 1 | 0 | 1 |
| New.CleanUp.ReferenceOTU5916 | 0 | 0 | 0 | 1 | 0 | 1 | 1 |
| New.CleanUp.ReferenceOTU9188 | 0 | 0 | 1 | 0 | 1 | 1 | 1 |
| New.CleanUp.ReferenceOTU14248 | 0 | 0 | 1 | 0 | 1 | 1 | 1 |
| New.CleanUp.ReferenceOTU58302 | 0 | 1 | 0 | 1 | 1 | 0 | 0 |
| New.CleanUp.ReferenceOTU71061 | 0 | 0 | 0 | 1 | 1 | 1 | 1 |
| FR874720.1.1807 | 1 | 1 | 1 | 1 | 1 | 1 | 1 |
| New.ReferenceOTU27 | 1 | 1 | 1 | 1 | 1 | 1 | 1 |
| New.CleanUp.ReferenceOTU135 | 0 | 1 | 0 | 0 | 0 | 1 | 1 |
| New.CleanUp.ReferenceOTU2517 | 0 | 1 | 0 | 0 | 0 | 0 | 1 |
| New.CleanUp.ReferenceOTU4326 | 0 | 0 | 1 | 1 | 0 | 0 | 1 |
| New.CleanUp.ReferenceOTU6927 | 0 | 0 | 1 | 1 | 0 | 1 | 0 |
| New.CleanUp.ReferenceOTU11904 | 0 | 0 | 0 | 0 | 0 | 1 | 1 |
| New.CleanUp.ReferenceOTU13195 | 0 | 0 | 0 | 0 | 1 | 1 | 1 |
| New.CleanUp.ReferenceOTU15226 | 0 | 1 | 0 | 0 | 1 | 1 | 1 |
| New.CleanUp.ReferenceOTU16090 | 0 | 0 | 0 | 1 | 1 | 0 | 0 |
| New.CleanUp.ReferenceOTU19420 | 0 | 1 | 0 | 1 | 0 | 1 | 1 |
| New.CleanUp.ReferenceOTU19598 | 0 | 0 | 1 | 0 | 0 | 1 | 1 |
| New.CleanUp.ReferenceOTU20106 | 0 | 0 | 0 | 1 | 0 | 0 | 1 |
| New.CleanUp.ReferenceOTU25859 | 0 | 1 | 0 | 0 | 1 | 0 | 0 |
| New.CleanUp.ReferenceOTU27438 | 0 | 0 | 0 | 1 | 0 | 0 | 0 |
| New.CleanUp.ReferenceOTU27837 | 0 | 1 | 0 | 0 | 0 | 0 | 0 |
| New.CleanUp.ReferenceOTU28332 | 0 | 0 | 1 | 1 | 1 | 1 | 0 |
| New.CleanUp.ReferenceOTU30995 | 0 | 1 | 0 | 1 | 0 | 1 | 0 |
| New.CleanUp.ReferenceOTU34439 | 0 | 1 | 0 | 1 | 0 | 1 | 0 |
| New.CleanUp.ReferenceOTU34664 | 0 | 1 | 1 | 1 | 0 | 0 | 1 |
| New.CleanUp.ReferenceOTU46887 | 0 | 1 | 0 | 1 | 0 | 1 | 1 |
| New.CleanUp.ReferenceOTU47519 | 0 | 0 | 0 | 1 | 1 | 1 | 0 |
| New.CleanUp.ReferenceOTU48787 | 0 | 1 | 0 | 1 | 0 | 0 | 1 |
| New.CleanUp.ReferenceOTU49879 | 0 | 0 | 0 | 1 | 0 | 0 | 1 |
| New.CleanUp.ReferenceOTU50275 | 0 | 1 | 1 | 1 | 0 | 0 | 1 |
| New.CleanUp.ReferenceOTU50587 | 0 | 1 | 0 | 0 | 0 | 1 | 0 |
| New.CleanUp.ReferenceOTU52175 | 0 | 0 | 0 | 0 | 1 | 0 | 1 |
| New.CleanUp.ReferenceOTU53389 | 0 | 1 | 0 | 1 | 1 | 0 | 0 |
| New.CleanUp.ReferenceOTU53718 | 0 | 1 | 0 | 1 | 0 | 0 | 0 |
| New.CleanUp.ReferenceOTU55483 | 0 | 0 | 0 | 1 | 1 | 1 | 1 |
| New.CleanUp.ReferenceOTU56182 | 0 | 1 | 1 | 1 | 0 | 1 | 1 |
| New.CleanUp.ReferenceOTU58329 | 0 | 1 | 0 | 1 | 1 | 1 | 1 |
| New.CleanUp.ReferenceOTU58417 | 0 | 0 | 0 | 1 | 1 | 0 | 1 |
| New.CleanUp.ReferenceOTU59084 | 0 | 1 | 0 | 0 | 0 | 0 | 0 |
| New.CleanUp.ReferenceOTU59297 | 0 | 1 | 0 | 0 | 1 | 1 | 1 |
| New.CleanUp.ReferenceOTU61006 | 0 | 1 | 0 | 0 | 0 | 0 | 1 |
| New.CleanUp.ReferenceOTU62623 | 0 | 1 | 0 | 0 | 1 | 1 | 1 |
| New.CleanUp.ReferenceOTU64253 | 0 | 1 | 0 | 1 | 0 | 1 | 1 |
| New.CleanUp.ReferenceOTU68067 | 0 | 0 | 1 | 1 | 0 | 1 | 1 |
| New.CleanUp.ReferenceOTU69092 | 0 | 0 | 1 | 1 | 0 | 0 | 0 |
| New.CleanUp.ReferenceOTU69718 | 0 | 0 | 0 | 0 | 0 | 1 | 0 |
| New.CleanUp.ReferenceOTU70900 | 0 | 1 | 0 | 0 | 0 | 1 | 1 |
| New.CleanUp.ReferenceOTU72900 | 0 | 1 | 0 | 0 | 0 | 0 | 1 |
| New.CleanUp.ReferenceOTU76509 | 0 | 1 | 1 | 0 | 0 | 0 | 1 |
| New.CleanUp.ReferenceOTU78757 | 0 | 1 | 1 | 1 | 0 | 1 | 0 |
| New.CleanUp.ReferenceOTU79101 | 0 | 0 | 0 | 0 | 0 | 1 | 1 |
| New.CleanUp.ReferenceOTU9591 | 0 | 0 | 1 | 1 | 1 | 0 | 1 |
| New.CleanUp.ReferenceOTU27075 | 0 | 0 | 1 | 0 | 0 | 0 | 1 |
| New.CleanUp.ReferenceOTU37275 | 0 | 1 | 0 | 1 | 1 | 0 | 1 |
| New.CleanUp.ReferenceOTU47814 | 0 | 1 | 0 | 1 | 0 | 0 | 0 |
| New.CleanUp.ReferenceOTU54871 | 0 | 1 | 0 | 1 | 1 | 0 | 1 |
| New.CleanUp.ReferenceOTU55187 | 0 | 0 | 1 | 0 | 0 | 1 | 1 |
| New.CleanUp.ReferenceOTU59603 | 0 | 0 | 1 | 1 | 1 | 0 | 0 |
| New.CleanUp.ReferenceOTU62498 | 0 | 0 | 1 | 1 | 0 | 1 | 1 |
| New.CleanUp.ReferenceOTU79885 | 0 | 1 | 0 | 0 | 0 | 1 | 0 |
| New.CleanUp.ReferenceOTU549 | 0 | 0 | 0 | 0 | 0 | 1 | 1 |
| New.CleanUp.ReferenceOTU14024 | 1 | 0 | 0 | 0 | 1 | 0 | 0 |
| New.CleanUp.ReferenceOTU36240 | 1 | 0 | 1 | 1 | 0 | 1 | 1 |
| New.CleanUp.ReferenceOTU50910 | 0 | 0 | 1 | 1 | 1 | 0 | 0 |
| AF438324.1.1768 | 0 | 1 | 0 | 0 | 0 | 0 | 0 |
| New.ReferenceOTU241 | 0 | 1 | 0 | 0 | 0 | 0 | 0 |
| New.CleanUp.ReferenceOTU3205 | 0 | 1 | 0 | 0 | 0 | 0 | 0 |
| New.CleanUp.ReferenceOTU11875 | 0 | 1 | 0 | 0 | 0 | 0 | 0 |
| New.CleanUp.ReferenceOTU12599 | 0 | 1 | 0 | 0 | 0 | 0 | 0 |
| New.CleanUp.ReferenceOTU14536 | 0 | 1 | 0 | 0 | 0 | 0 | 0 |
| New.CleanUp.ReferenceOTU16997 | 0 | 1 | 0 | 0 | 0 | 0 | 0 |
| New.CleanUp.ReferenceOTU17869 | 0 | 1 | 0 | 0 | 0 | 0 | 0 |
| New.CleanUp.ReferenceOTU30628 | 0 | 1 | 0 | 0 | 0 | 0 | 0 |
| New.CleanUp.ReferenceOTU32403 | 0 | 1 | 0 | 0 | 0 | 0 | 0 |
| New.CleanUp.ReferenceOTU38518 | 0 | 1 | 0 | 0 | 0 | 0 | 0 |
| New.CleanUp.ReferenceOTU41737 | 0 | 1 | 0 | 0 | 0 | 0 | 0 |
| New.CleanUp.ReferenceOTU44302 | 0 | 1 | 0 | 0 | 0 | 0 | 0 |
| New.CleanUp.ReferenceOTU47411 | 0 | 1 | 0 | 0 | 0 | 0 | 0 |
| New.CleanUp.ReferenceOTU52999 | 0 | 1 | 0 | 0 | 0 | 0 | 0 |
| New.CleanUp.ReferenceOTU65843 | 0 | 1 | 0 | 0 | 0 | 0 | 0 |
| New.CleanUp.ReferenceOTU75932 | 0 | 1 | 0 | 0 | 0 | 0 | 0 |
| New.CleanUp.ReferenceOTU22031 | 1 | 1 | 0 | 0 | 0 | 0 | 0 |
| New.CleanUp.ReferenceOTU76591 | 1 | 1 | 0 | 0 | 0 | 0 | 0 |
| New.ReferenceOTU9 | 1 | 1 | 0 | 0 | 0 | 0 | 0 |
| New.ReferenceOTU109 | 1 | 1 | 0 | 1 | 1 | 1 | 1 |
| New.ReferenceOTU248 | 1 | 1 | 0 | 0 | 0 | 0 | 0 |
| New.ReferenceOTU77 | 1 | 1 | 0 | 1 | 1 | 1 | 1 |
| New.CleanUp.ReferenceOTU16303 | 0 | 1 | 0 | 1 | 1 | 1 | 0 |
| New.CleanUp.ReferenceOTU27507 | 1 | 0 | 0 | 0 | 0 | 0 | 0 |
| New.CleanUp.ReferenceOTU28592 | 1 | 0 | 0 | 0 | 0 | 0 | 0 |
| New.CleanUp.ReferenceOTU41095 | 1 | 0 | 0 | 0 | 0 | 0 | 0 |
| New.CleanUp.ReferenceOTU41188 | 1 | 0 | 0 | 0 | 0 | 0 | 0 |
| New.CleanUp.ReferenceOTU48123 | 1 | 0 | 0 | 0 | 0 | 0 | 0 |
| New.CleanUp.ReferenceOTU48962 | 1 | 0 | 0 | 0 | 0 | 0 | 0 |
| New.CleanUp.ReferenceOTU66362 | 1 | 0 | 0 | 0 | 0 | 0 | 0 |
| New.CleanUp.ReferenceOTU66499 | 1 | 0 | 0 | 0 | 0 | 0 | 0 |
| New.CleanUp.ReferenceOTU73619 | 1 | 0 | 0 | 0 | 0 | 0 | 0 |
