# Supplementary material for: Environmental DNA detects biodiversity and ecological features of phytoplankton communities in Mediterranean transitional waters
Source: Sci Rep. 2023 Sep 14;13:15192. doi: 10.1038/s41598-023-42389-3 (PMC10502138; doi:10.1038/s41598-023-42389-3)
Supplement: Supplementary file 5 — Supplementary Table S5. [file 41598_2023_42389_MOESM5_ESM.docx]

**Table S5.** Presence-Absence matrix focused on the OTUs annotated as *Tetraselmis* genus in the seven sampling sites. Green stands for presence, while red stands for absence.

| OTU reference number | Name | AF | A | B | C | D | E | F |
| --- | --- | --- | --- | --- | --- | --- | --- | --- |
| EF527133.1.1840 | Tetraselmis 1 | 1 | 1 | 1 | 1 | 1 | 1 | 0 |
| AF166380.1.1793 | Tetraselmis 2 | 1 | 1 | 1 | 1 | 1 | 1 | 1 |
| U05039.1.1805 | Tetraselmis 3 | 1 | 1 | 1 | 1 | 1 | 1 | 0 |
| KU561107.1.1719 | Tetraselmis 4 | 1 | 0 | 1 | 0 | 0 | 0 | 0 |
| New.ReferenceOTU149 | Tetraselmis 5 | 1 | 1 | 1 | 1 | 1 | 1 | 0 |
| New.CleanUp.ReferenceOTU23592 | Tetraselmis 6 | 1 | 0 | 0 | 0 | 0 | 0 | 0 |
| New.CleanUp.ReferenceOTU26017 | Tetraselmis 7 | 1 | 0 | 0 | 0 | 1 | 0 | 0 |
| New.CleanUp.ReferenceOTU78860 | Tetraselmis 8 | 1 | 0 | 0 | 0 | 0 | 0 | 0 |
